# Supplementary material for: Healthcare providers’ knowledge on sickle cell disease and its management: A pre- and post-training test evaluation outcome
Source: PLoS One. 2025 Sep 8;20(9):e0332069. doi: 10.1371/journal.pone.0332069 (PMC12416636; doi:10.1371/journal.pone.0332069)
Supplement: S1 Appendix — (DOCX) [file pone.0332069.s001.docx]

**Appendix**

**Test Questionnaire (Answers in bold italic font)**

1.       Which of these is NOT a recognized clinical presentation of sickle cell disease?

1. Pain
2. Jaundice
3. Stroke
4. Bedwetting
5. ***Psychosis***

2.       The following are complications of SCD EXCEPT

1. Delayed puberty
2. Erectile dysfunction
3. ***Acute leukaemia***
4. Gall stones
5. Depression

3.       Sickle cell retinopathy is commonest in which of the following Hb genotypes?

1. Hb AA
2. Hb AS
3. Hb SS
4. ***Hb SC***
5. Hb SB thal

4.       Which of the following vaccines are indicated after a splenectomy?

a.       Meningococal

b.       B.Haemophilus influenzae

c.       Pneumococal

d.       Hepatitis B

***e.       A, B and C***

5.       Hydroxyurea is used to manage acute anemia in Sickle cell anaemia

a.       True

***b.        False***

6.       Aplastic Crisis affects more adults than children

a.       True

***b.       False***

7.       Patients who are allergic to penicillin V can substitute it with;

***a.       Clarithromycin***

b.       Cefuroxime

c.       Orelox

d.       Septrin

e.       Amoxiclav

8.       Which tests can be used to confirm a diagnosis of SCD?

a.       Sickling test

b.       Hb electrophoresis

c.       HPLC

d.       A, B and C

***e.       B and C***

9.       Which of these testing methods is appropriate in the diagnosis of SCD in infants less than 6 months of age?

a.       Hb electrophoresis

b.       Hb S Solubility test

***c.       Isoelectric focusing***

d.       Full blood count

e.       Sickling test

10.   The least useful of tests in the diagnosis of heterozygous S/B thalassaemia is;

a.       FBC

***b.       HBA1c***

c.       Hb electrophoresis

d.       HPLC

e.       DNA test

11.   Which of these measures is not required to maintain steady state in SCD?

a.       Folic acid supplementation

***b.       Routine iron supplementatio***n

c.       Penicillin prophylaxis

d.       Adequate hydration

e.       Hydroxyurea therapy

12.   Mental health assessment and psychosocial support is not essential in the management of SCD

a.       True

***b.***       ***False***

c.       Don’t know

13.   Non-pharmacological measures to prevent SCD crises include all the following except.

a.       Adequate hydration

b.       Massage of the limbs

***c.       Intake of pain medications***

d.       Avoidance of extremes of temperature

e.       Stress reduction

14.   Principles in the management of acute chest syndrome include all **EXCEPT**;

***a.       Hyperhydration***

b.       Antibiotics

c.       Oxygen supplementation

d.       Exchange /top up blood transfusion

e.       Incentive spirometry

15.   The potential for cure for sickle cell disease is which of the following?

a.       Increased folate and iron in the diet

b.       Repeated blood transfusions

c.       Pneumococcal immunizations

***d.       Bone marrow transplant***

e.       Hyperhydration

16.   A seven-year old with SCD is admitted for IV fluids and pain management. During the night, the child wakes up complaining of chest pain and difficulty in breathing. Which of the following will possibly be occurring?

a.       The child doesn’t seem to be well controlled with the medications given an hour ago

***b.       The child is describing symptoms associated with acute chest syndrome***

c.       The child is describing symptoms associated with splenic sequestration

d.       The child wasn’t put on supplemental oxygen early enough

e.       The child didn’t receive adequate hydration for the pain management

17.   A 14-year-old boy has just been prescribed hydroxyurea. Which of the following explanation best describes the purpose for the hydroxyurea in managing sickle cell anaemia.

a.       Hydroxyurea is a pain medication that will help relieve your pain

b.       Hydroxyurea is a medication that works by increasing the RBC ability to absorb fluid to prevent sickling

***c.       Hydroxyurea helps your sickle cell by increasing fetal haemoglobin , which can’t sickle***

d.       Hydroxyurea is a new medication that works within the red blood cell to change its structure and make it less prone to sickling.

e.       Hydroxyurea is a haematinic and increases the haemoglobin level

18.   The following can be described as Sickle Cell Anaemia

a.       SCD- SC

***b.       SCD-SS***

c.       SCD-AS

d.       SCD-AC

e.       SCD-SD

19.   Screening for stroke in SCD involve the following;

a.       Skull Xray

b.       Fundoscopy

***c.       Transcranial doppler ultrasound scanning***

d.       Complete blood count

e.       Biochemistry (LFT, RFT)

20.   Which of the following is the most important principle of management of SCD?

a.       Prophylaxis against bacterial infections and malaria

b.       Prompt treatment of acute bacterial and malarial infection

c.       Pain control

d.       Limiting blood transfusion to severe, life-threatening anemia

***e.       All of the above***
