# Supplementary material for: Healthcare providers’ knowledge on sickle cell disease and its management: A pre- and post-training test evaluation outcome
Source: PLoS One. 2025 Sep 8;20(9):e0332069. doi: 10.1371/journal.pone.0332069 (PMC12416636; doi:10.1371/journal.pone.0332069)
Supplement: S3 Appendix — (PDF) [file pone.0332069.s003.pdf]

Kwame Nkrumah
University of Science
and Technology, Kumasi

College of Health Sciences
SCHOOL OF MEDICINE AND DENTISTRY

COMMITTEE ON HUMAN RESEARCH, PUBLICATION AND ETHICS

Our Ref: CHRPE/AP/273/24

15th April 2024.

Prof. Fred Stephen Sarfo
Department of Medicine
School of Medicine and Dentistry
KNUST-KUMASI

Dear Sir,

LETTER OF APPROVAL

Protocol Renewal: ***"Ghana Sickle Pan-African Research Consortium Study (GHANA-SPARCO)."***

Proposed Site: ***Komfo Anokye Teaching Hospital, Kumasi and Korle Bu Teaching Hospital, Accra.***

Sponsor: ***National Heart, Lung, and Blood Institute; National Institute of Health.***

Your submission to the Committee on Human Research, Publication and Ethics on renewal to protocol No. CHRPE/AP/ 088/23 dated 3rd February 2023 refers.

The Committee reviewed the following documents:

- A notification letter of 1st June, 2020 from the Komfo Anokye Teaching Hospital (study site) indicating approval for the conduct of the study at the Hospital.
- A notification letter of 21st May, 2020 from the Korle Bu Teaching Hospital (study site) indicating approval for the conduct of the study at the Hospital.
- A Completed CHRPE Application Form.
- Participant Information Leaflet and Consent Form.
- Research Protocol.
- Questionnaire.

The Committee has considered the ethical merit of your proposed renewal and approved it. The approval is for a fixed period of one year, beginning **3rd February 2024** to **2nd February 2025** renewable thereafter. The Committee may however, suspend or withdraw ethical approval at any time if your study is found to contravene the approved protocol.

Data gathered for the study should be used for the approved purposes only. Permission should be sought from the Committee if any amendment to the protocol or use, other than submitted, is made of your research data.

The Committee expects a report on your study annually or at the close of the project, whichever one comes first. It should also be informed of any publication arising from the study.

Thank you, Sir for your application.

Yours faithfully,

Rev. Prof. John Appiah-Poku
Honorary Secretary
FOR: CHAIRMAN

Room 7, Block L, School of Medicine and Dentistry, KNUST, University Post Office, Kumasi, Ghana
Tel: +233 (0) 3220 63248 **Mobile:** +233 (0) 20 5453785 **Email:** chrpe.knust.kath@gmail.com/chrpe@knust.edu.gh
