# Supplementary material for: Healthcare providers’ knowledge on sickle cell disease and its management: A pre- and post-training test evaluation outcome
Source: PLoS One. 2025 Sep 8;20(9):e0332069. doi: 10.1371/journal.pone.0332069 (PMC12416636; doi:10.1371/journal.pone.0332069)
Supplement: S4 Appendix — (PDF) [file pone.0332069.s004.pdf]

1. Overview and pathophysiology_SoC training.pptx

2. SCD DIAGNOSIS-SOC GUIDELINES TRAINING_Dr Ahmed.pptx

3. Health maintenance_SoC training.pptx

4a. Acute Pain in SCD.pptx

4b. SoC training ACS.pptx

5. SoC training acute anaemia-1.pptx

6. Stroke In Scd 1.pptx

7. Chronic complications SOC training.pptx

Disclaimer

- Authors make no claim on the images used in this presentation.
- All images are only being used for teaching purposes.
- No infringement on copyright is intended.

Sickle In Africa

Sickle Cell Disease-Overview and Pathophysiology

Dr Vivian Paintsil

Learning Objectives

- To know what SCD is and the underlying pathophysiology
- Improve our knowledge of the genetics and hereditary patterns of SCD

SCD in Africa

WORLD HEALTH ORGANIZATION

FIFTY-NINTH WORLD HEALTH ASSEMBLY A59/9
Provisional agenda item 11.4 24 April 2006

Sickle-cell anaemia

Report by the Secretariat

When health impact is measured by under-five mortality, sickle-cell anaemia contributes the equivalent of 5% of under five deaths on the African continent, more than 9% of such deaths in West Africa, and up to 16% of under-five deaths in individual West African countries.

Sickle Cell Disease - Introduction

- SCD- generic term used when an individual has 2 abnormal Hb genes, at least one of which is HbS and the resulting symptomatology or pathology is due to the sickling phenomenon.

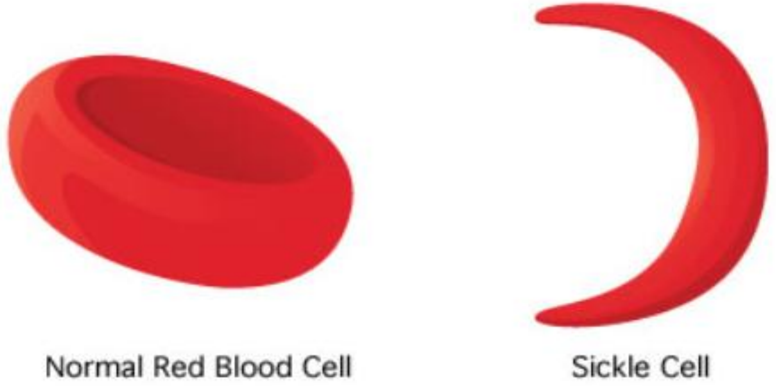

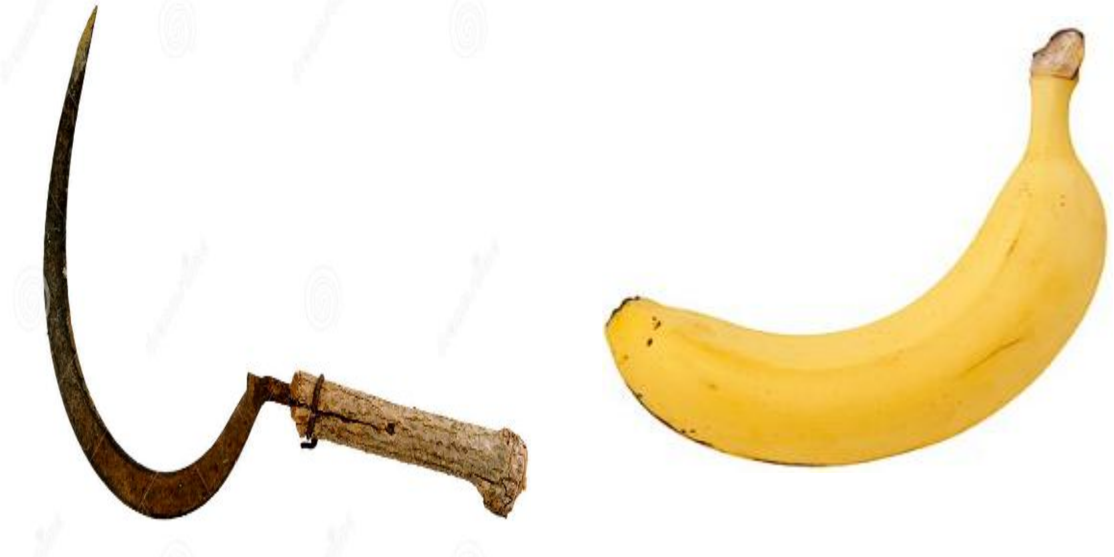

SCD in Local dialects

Nwewee
- Fanti

Ahotutuo
-Twi

Amosani
-Hausa

Nududui
-Ewe

Korba Baalun
-Dagaare

Nakinchensi
-Sisala

Gburigburihi
-Wale

Darimihi
-Dagbani

Tswetswetswe
-Ga

Do you see patients with SCD, What types of SCD do you know of?

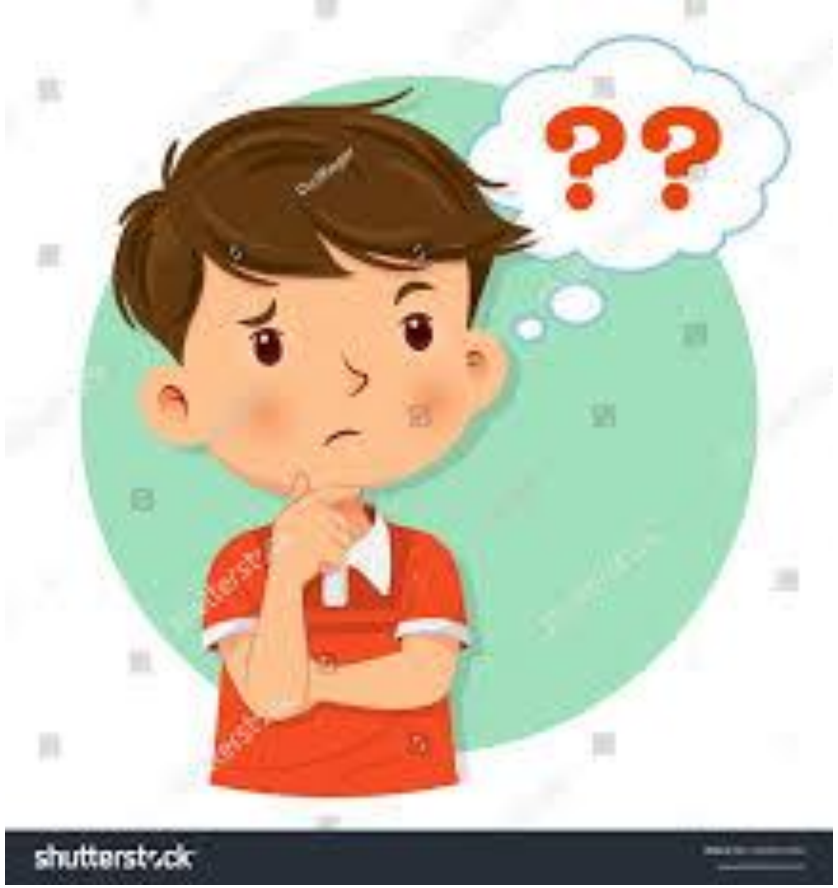

Common Types of SCD

SCD-SS	SCD-SC
SCD-Sβ^0 thalassemia	SCD-Sβ^+ thalassemia

Sickle Cell Disease in Africa
Burden of Disease

Prevalence of Sickle Cell Trait (AS)

North Africa	0.2 - 14.7
West Africa	1.7 - 30.7
Central Africa	0.6 - 27.0
East Africa	0.0 - 40.5
Southern Africa	0.0 - 36.7
US Newborns (all groups)	*1.6

*Estimates based on data from U.S. Census Bureau, 2000

Burden of Sickle Cell Disease

- Nigeria, India and DRC account for >50% of the births with SCD-SS in the world
- Over 300,000 babies born with SCD every year
  - **More than 1,000 babies per day**
- In the absence of national level surveillance, NBS data and registry, this maybe an under estimation

Sickle Cell Disease in Africa
Burden of Disease - Newborn Data

Prevalence of SCD in Newborns

Country	No. tested	FAS (AS) %	FAC (AC) %	FS (SS) %	FSC (SC) %	SCD (ALL)	Authors (Year)
Burkina Faso	2,341	7.14	16.67	0.6	1.15	1:57	Kafando et al, '05
Togo	385	18.7	8.9	1.0	1.3	1:43	North et al, '88
Congo (DRC)	31,204	16.9	-	1.4	-	1:71	Tshilolo, 09
Nigeria	644	20.6	1.1	2.8*	0.2	1:33*	Odunvbun, 09
Nigeria	3603	20.5	0.9	1.4	0.1	1:66	Nnodu, '20
Ghana	255,991	13.35	8.75	0.96	0.81	1:55	Ohene-Frempong MOH, '08
Ghana	528,735	13.25	8.96	0.90	0.81	1:58	2021
* Probable overestimate due to low number tested							

Closer look at what Sickle Cell does to the body

Hb S Polymerization, S-Cell Sickling

In a red blood cell containing mostly Hb S…

When deoxygenated

When oxygenated…

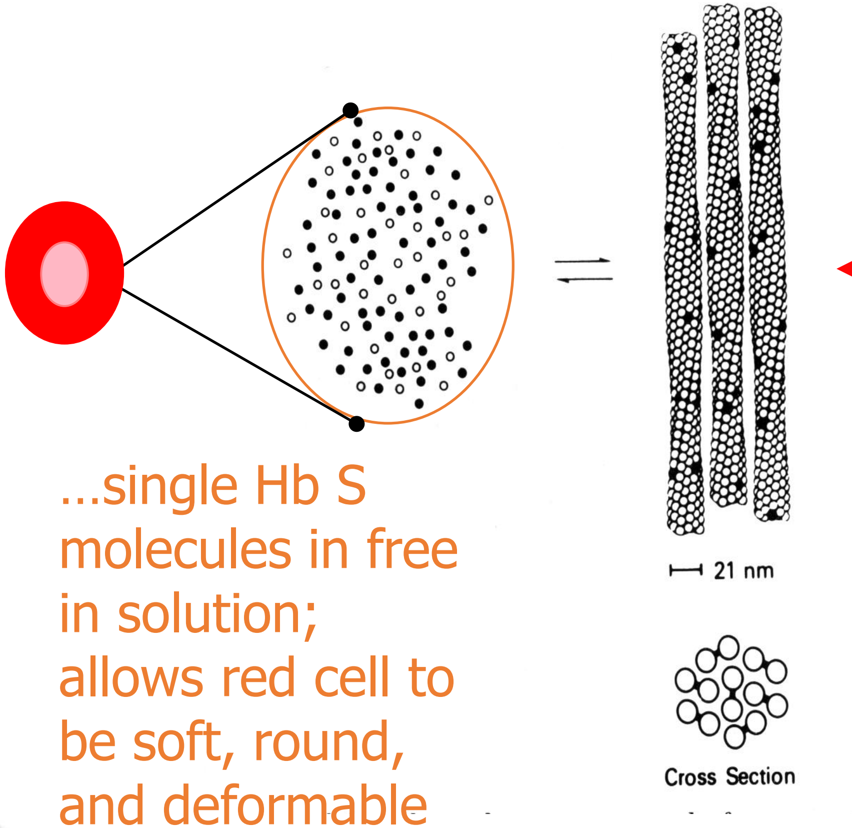

+ O_2

- O_2

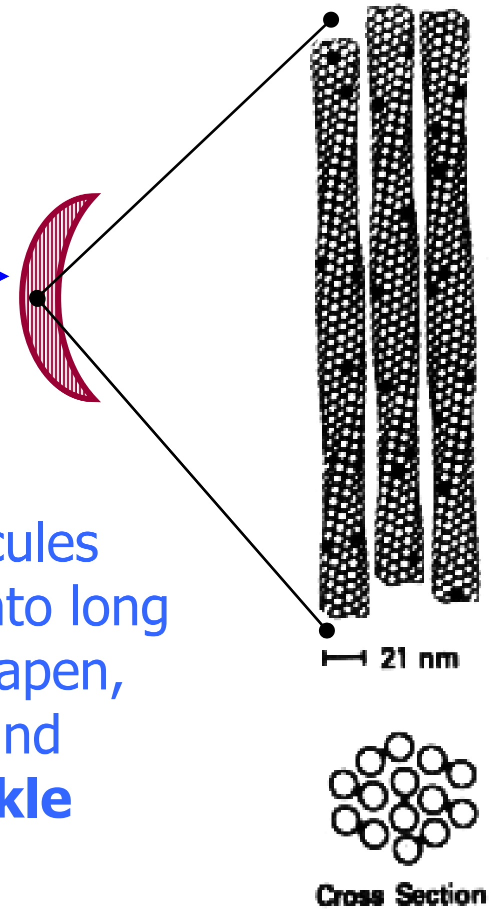

…single Hb S molecules in free in solution; allows red cell to be soft, round, and deformable

…Hb S molecules polymerize into long fibers; misshapen, dehydrated and adherent **sickle cells.**

Pathophysiology

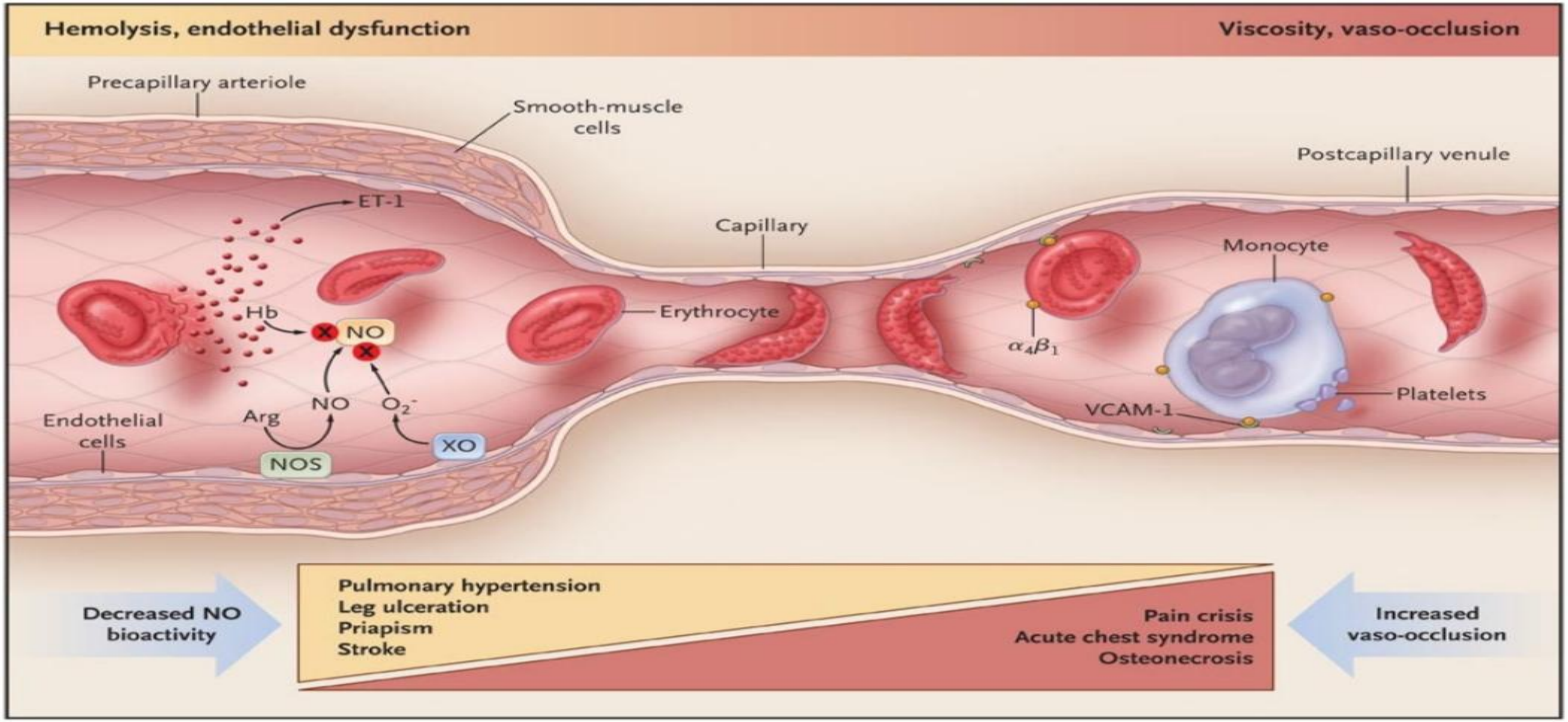

Pathophysiology - Vaso-occlusion

Silent, ongoing blockage of blood vessels (i.e., vaso-occlusion) drives the chronic nature of SCD

- Ongoing vaso-occlusion can culminate in VOCs, the clinical hallmark of SCD
- These VOCs are unpredictable and painful events that can lead to medical intervention
- VOCs are associated with decreased patient quality of life and increased risk of organ damage and death

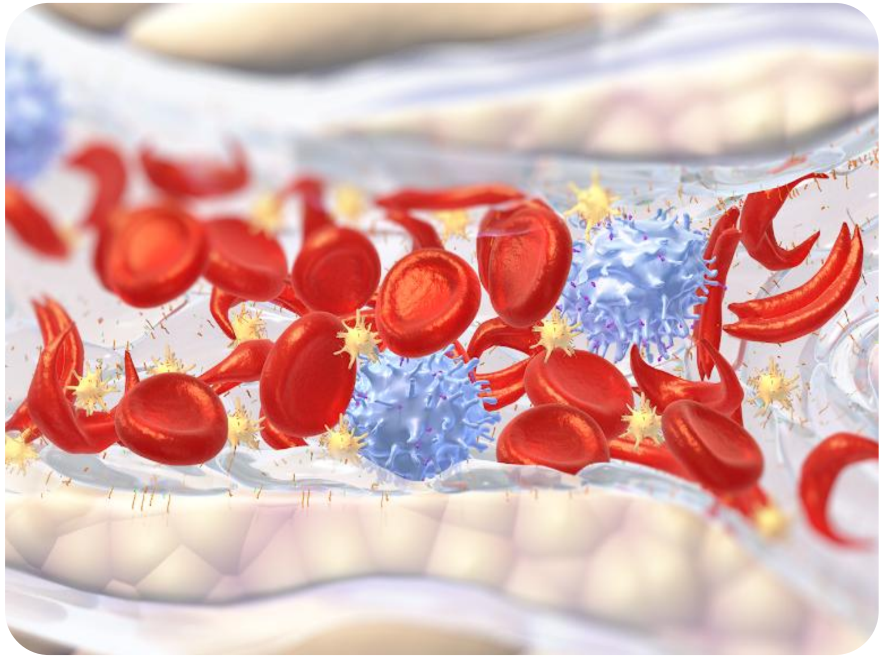

Haemolytic anaemia and Lifespan of sickled Cell

- SCA is considered an uncompensated hemolytic anemia

- Lifespan of normal RBCs → 120 days
- Lifespan of sickled RBCs → 10-20 days

- Typical CBC of child with SCA → low hemoglobin and high reticulocyte count

(Jakubik & Thompson, 2000; Kline, 2008)

Inheritance of Sickle Cell Disease

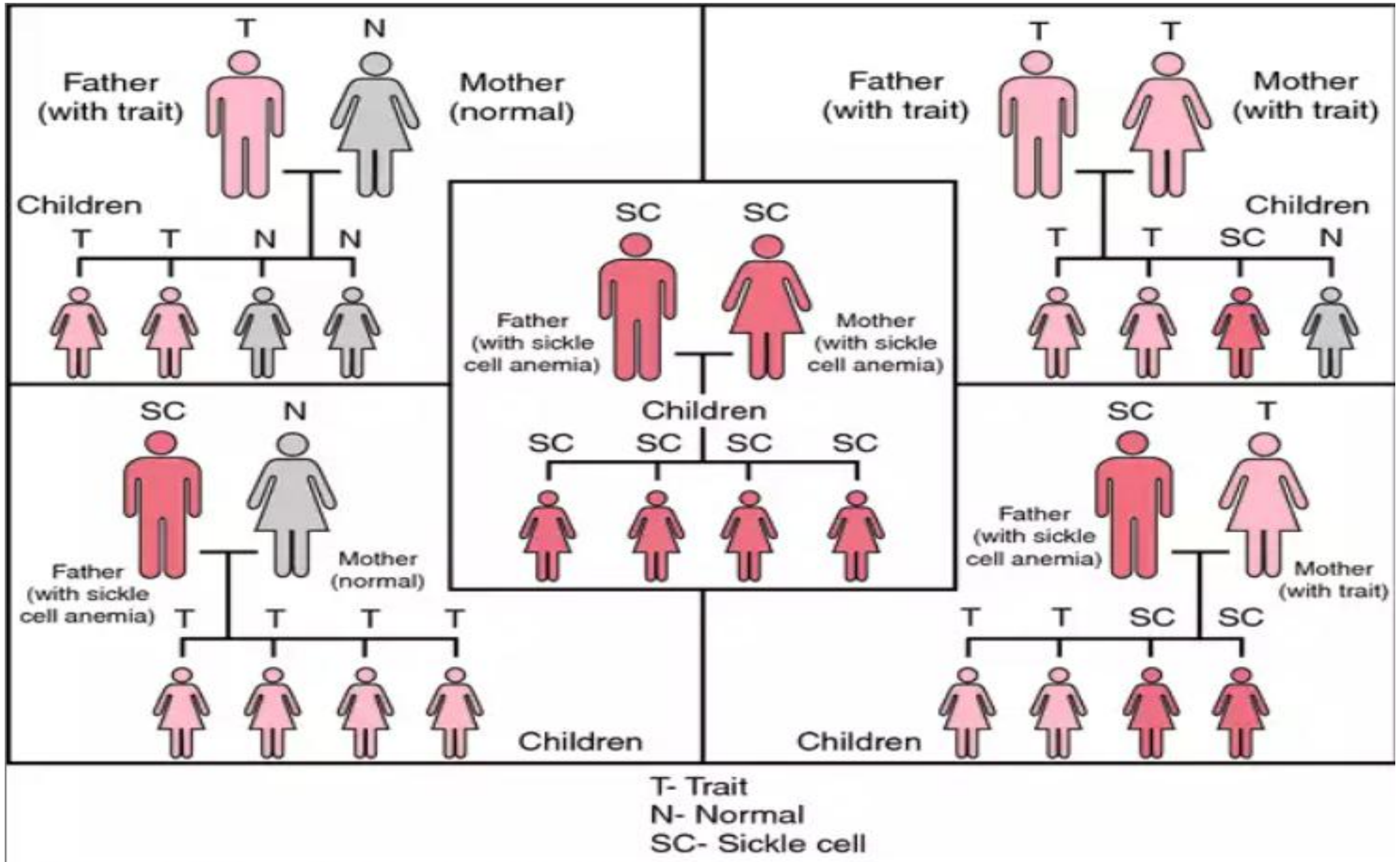

Statistical probabilities of inheriting sickle cell anemia.

Inheritance Box

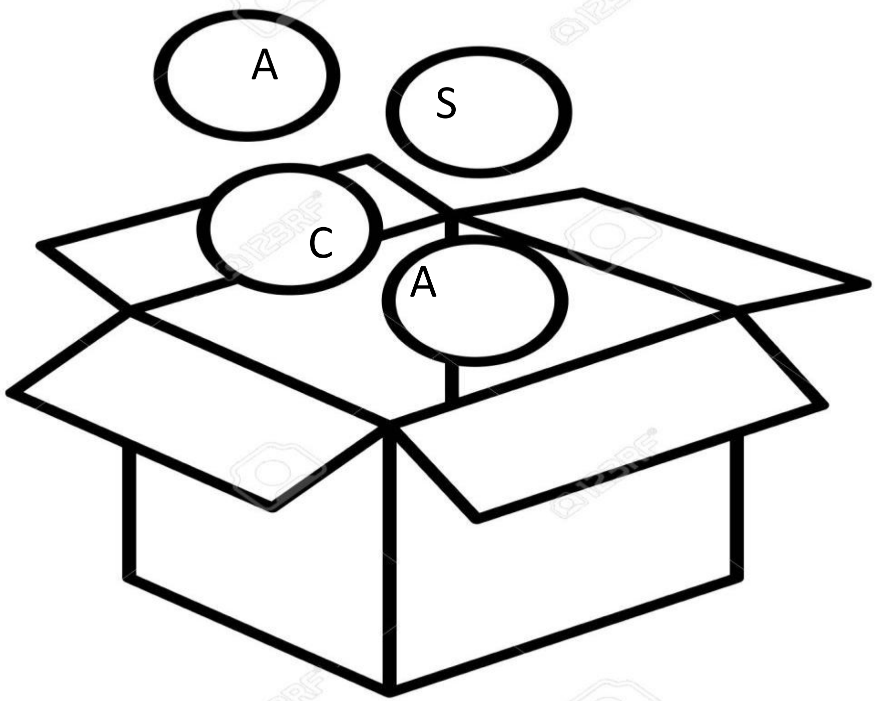

PUNETT SQUARE

	A	S
A	AA	AS
S	AS	SS

AA- 25%: has sickle cell disease

AS- 50%: has sickle cell trait

SS- 25%: does not have sickle cell disease or trait

AS x AS

parents carry trait for sickle cell disease!

Triggers of sickling

- Being at high altitudes (Eg. mountain climbing)
- Changes in temperature, eg. moving from a warm house into the cold weather not covered up or into the rain
- Infections
- Not having enough to drink or dehydration
- Stress

CONCLUSION

- SCD occurs when there is the inheritance of 2 abnormal genes one of which is HbS
- Silent, ongoing blockage of blood vessels (i.e., vaso-occlusion) drives the chronic nature of SCD
- Inheritance is autosomal recessive
- Triggers for sickling include extremes of temperature, dehydration, stress etc

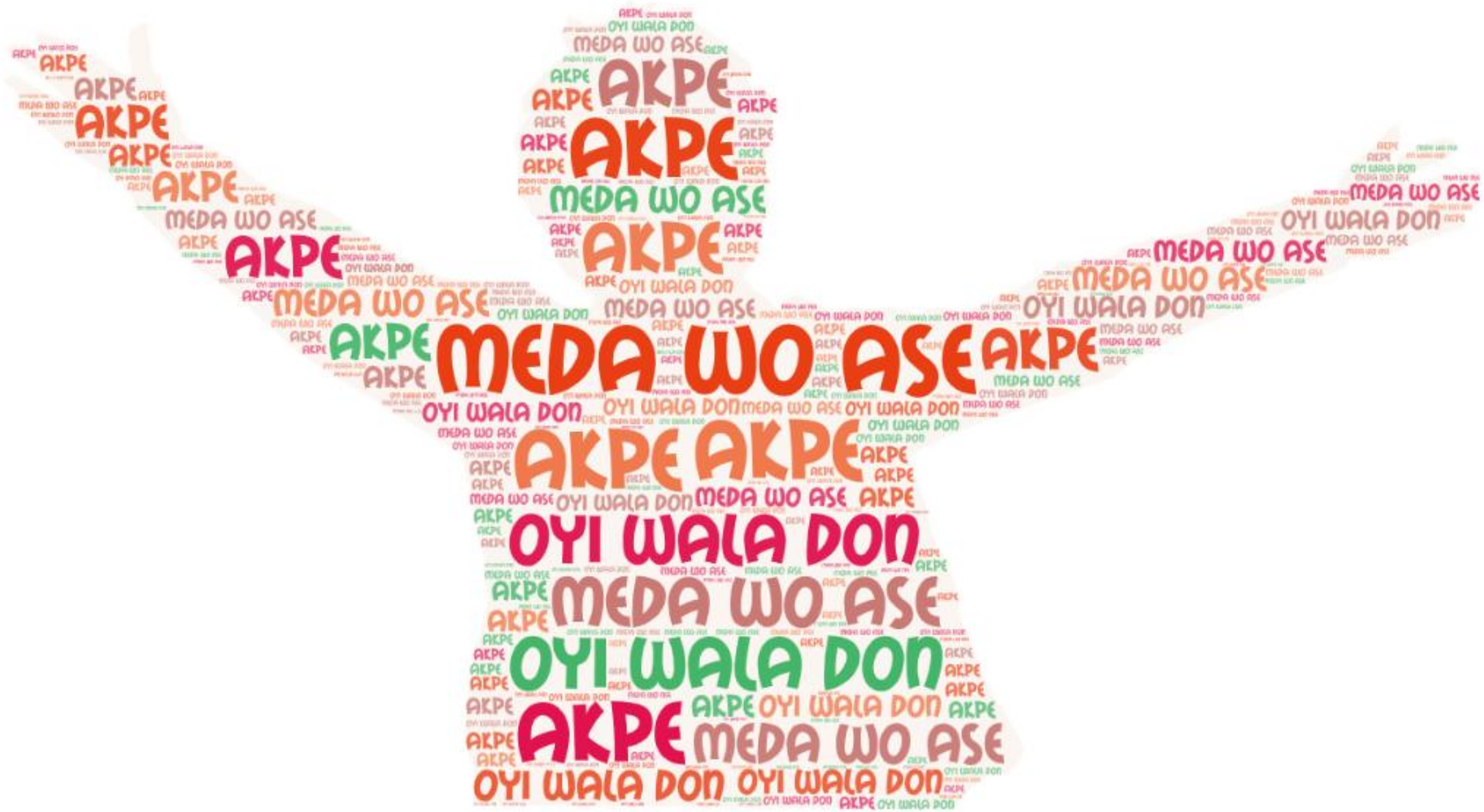
MEDA WO ASE
AKPE
OYI WALA DON

DIAGNOSIS OF SCD

Dr. Eunice A Ahmed

Question 1

1. Which tests can be used to confirm a diagnosis of SCD?
    a) Sickling test
    b) Hb electrophoresis
    c) HPLC
    d) A, B and C
    e) B and C

Question 2

2. Which of these testing methods is not appropriate in the diagnosis of SCD in infants less than 6 months of age?

a) Hb electrophersis
b) HPLC
c) Isoelectric focusing
d) Capillary electrophoresis

Question 3

3. These tests are useful in the diagnosis of heterozygous S/B thalassaemia except?

a) FBC
b) HBA1c
c) Hb electrophoresis
d) HPLC
e) DNA test

OUTLINE

HISTORY

PHYSICAL EXAM

LABORATORY TEST

LABORATORY TEST-METHODS

HAEMOGLOBIN TESTS

TESTS FOR S/THALASSAEMIAS

RAPID TESTS

DNA BASED TESTS

Haemoglobin tests

Hb tests

Used in the initial determination of Hb phenotype.

- Hb electrophoresis (HE)
- Isoelectric focusing (IEF)
- Capillary electrophoresis (CE)
- High-performance liquid chromatography (HLPC).

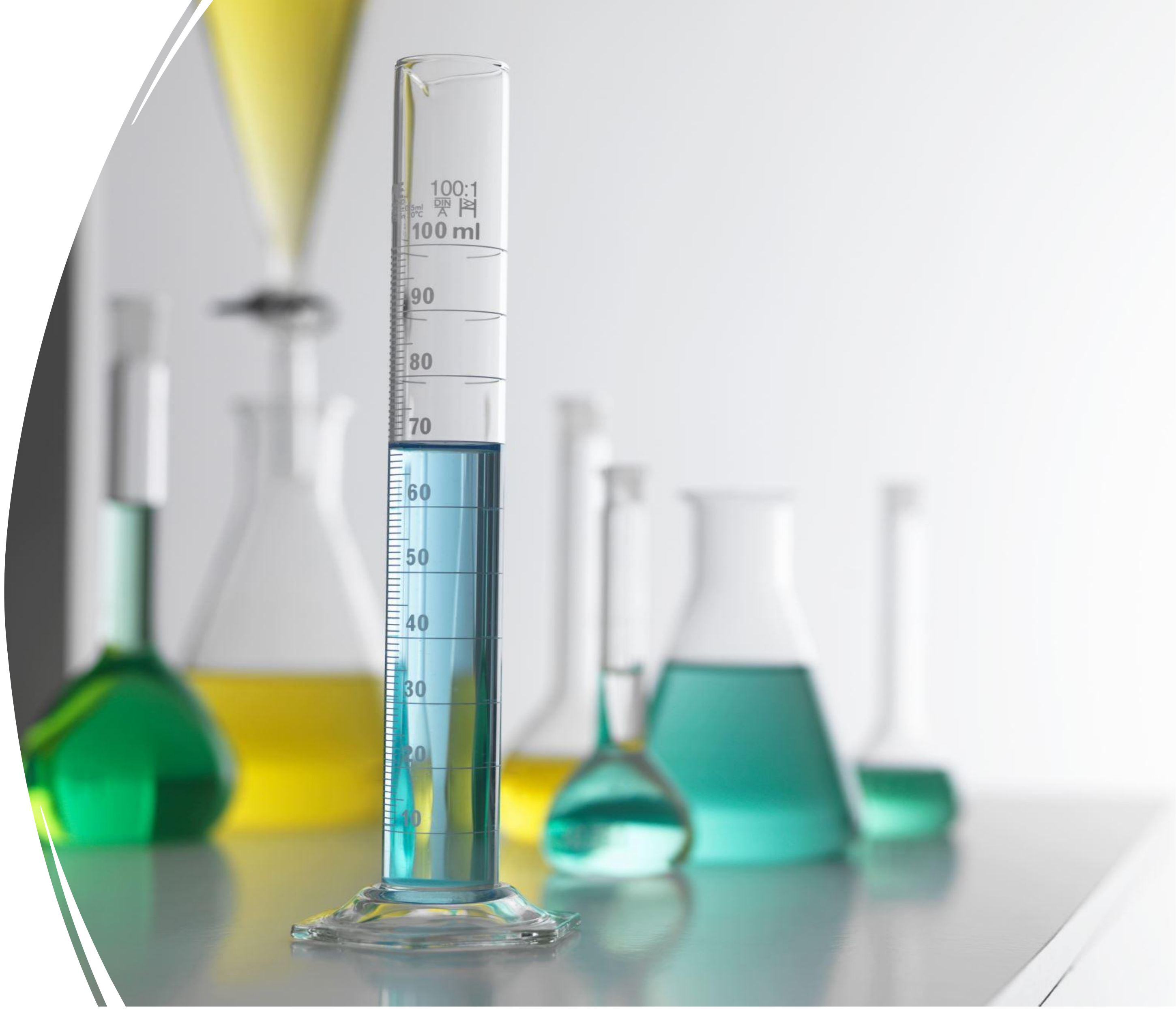

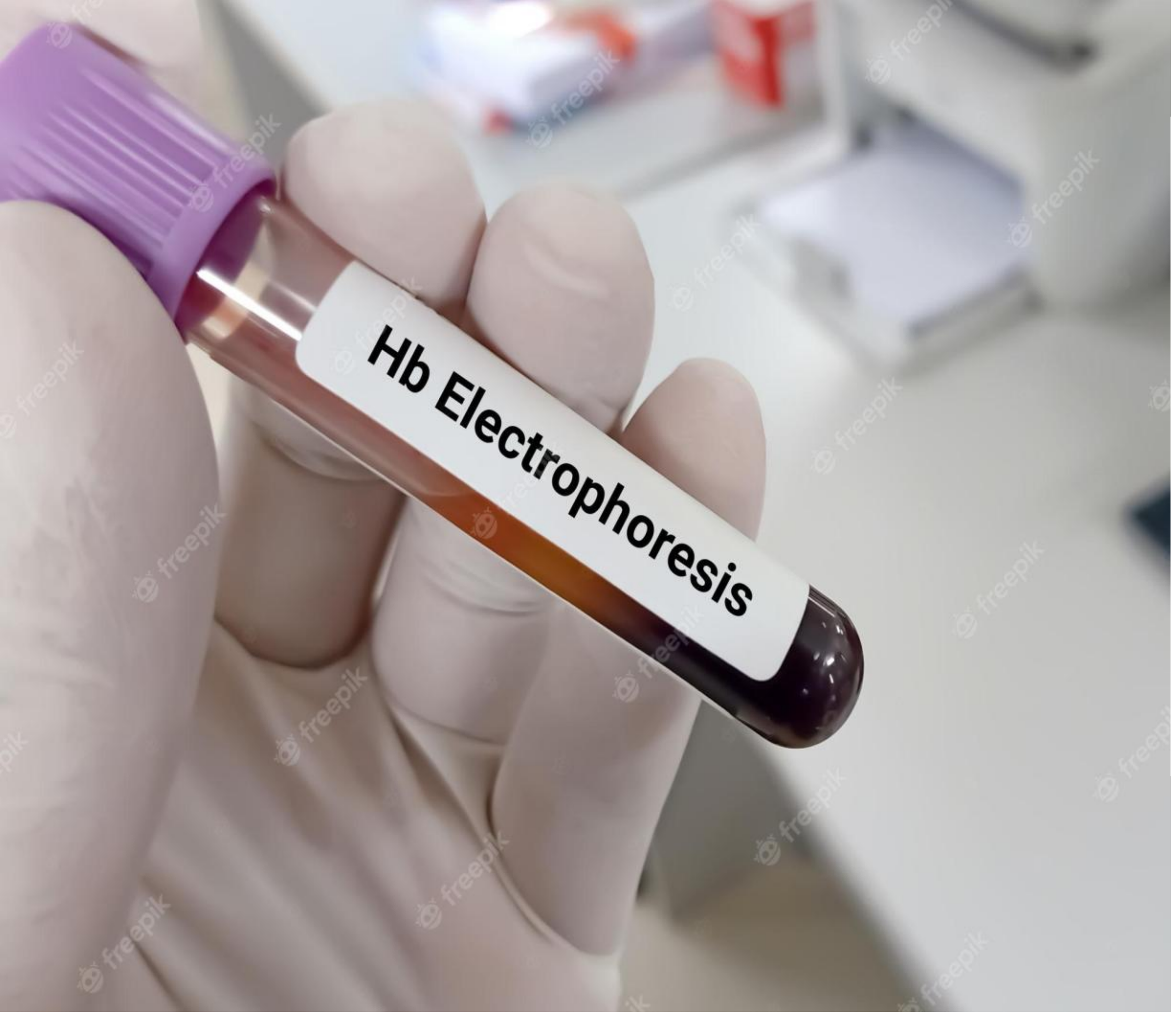

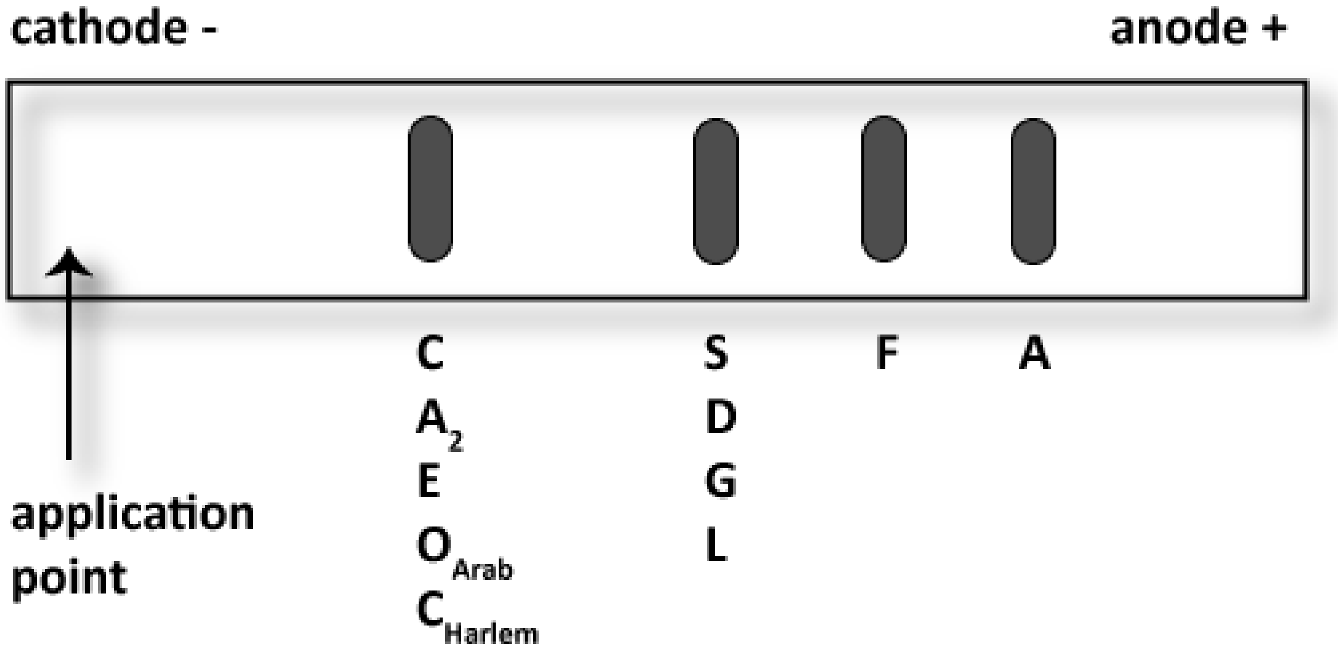

- Cellulose acetate Electrophoresis

Hemoglobin electrophoresis

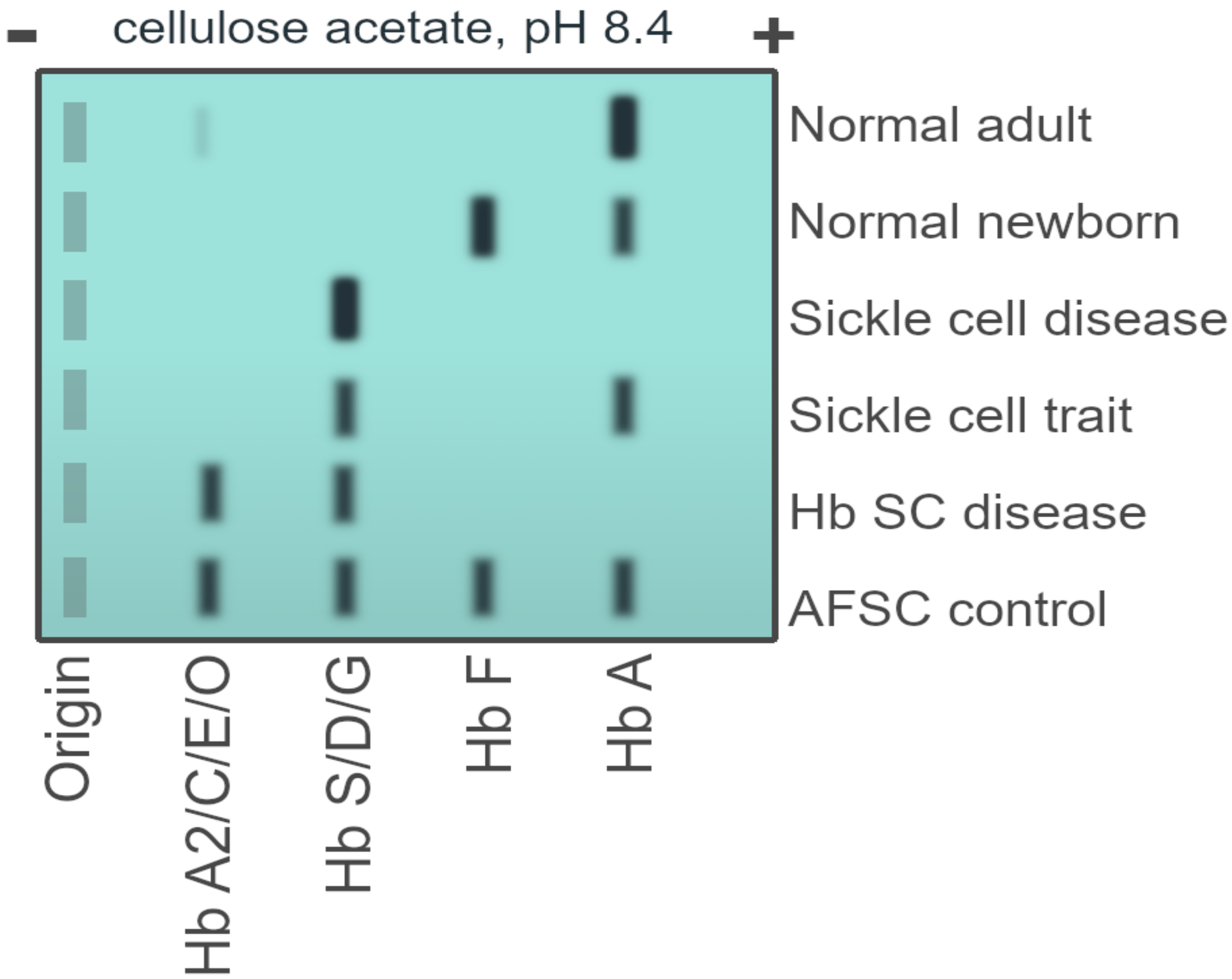

- Evaluate the Alkaline Electrophoresis

1. Which patient(s) has Sickle Cell Disease?
2. Which patient(s) has Sickle Cell Trait?
3. What type of hemoglobinopathy does patient #7 have?

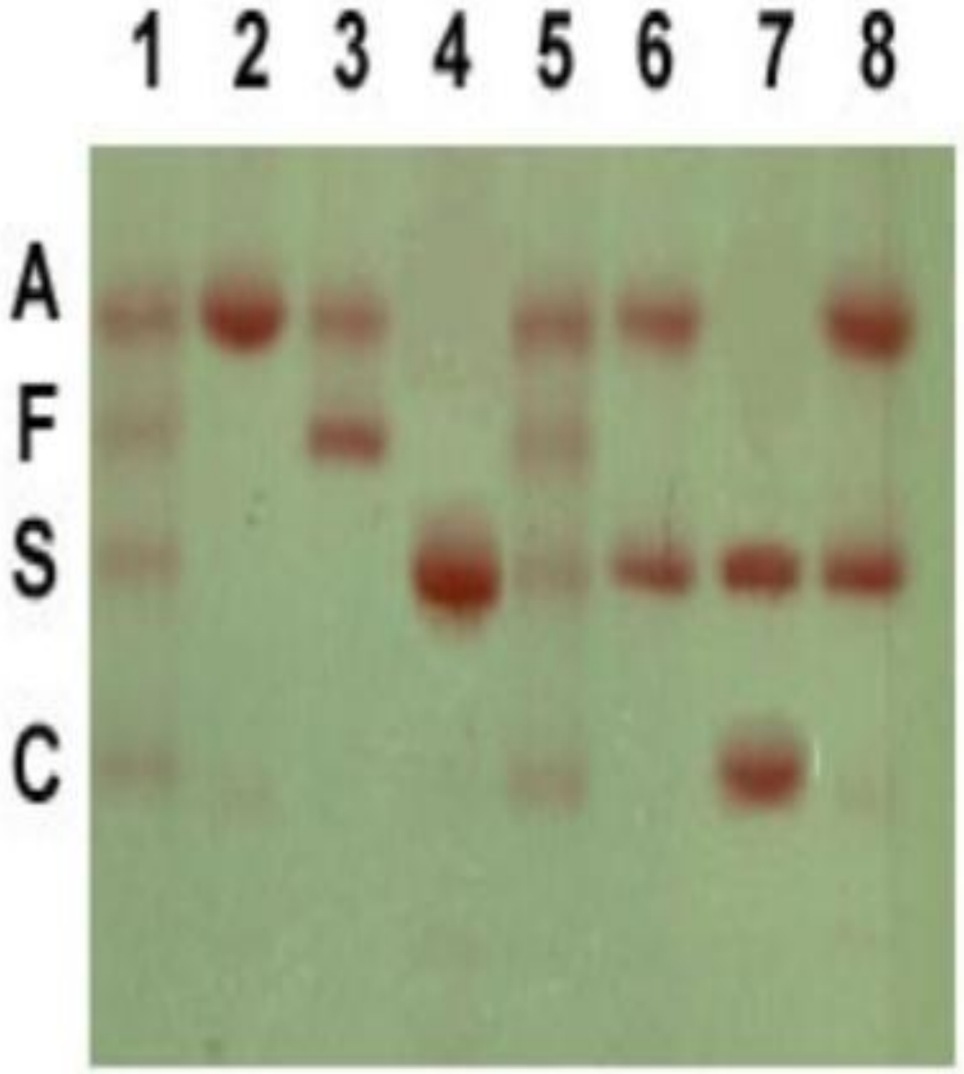

- Confirm results showing Hb variants using a method different from the original test as the Hb separation methods are seldom definitive.
- If CAE is used first, confirm with either HPLC/IEF/CE

Normal HPLC pattern

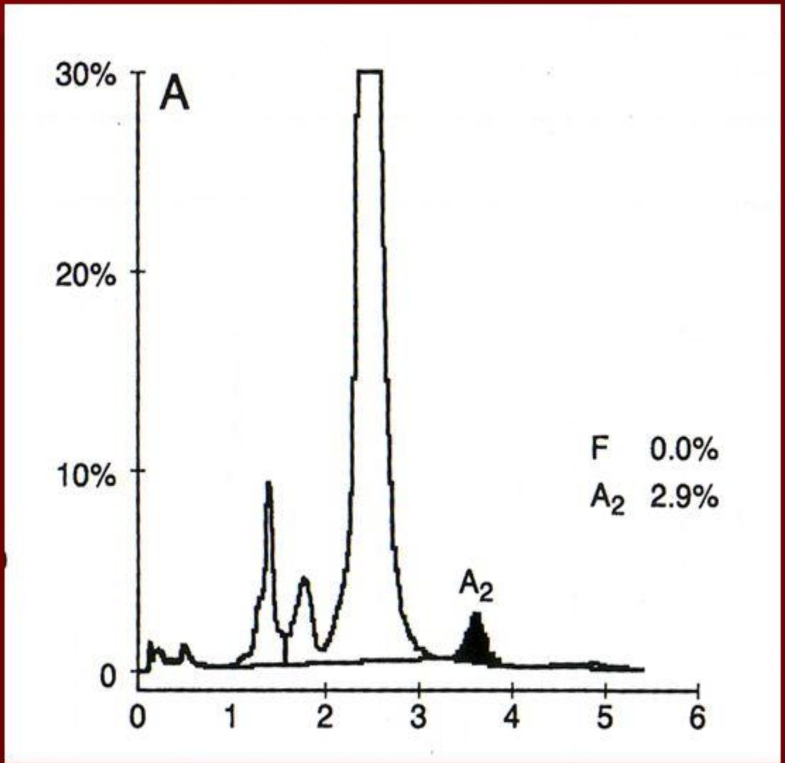

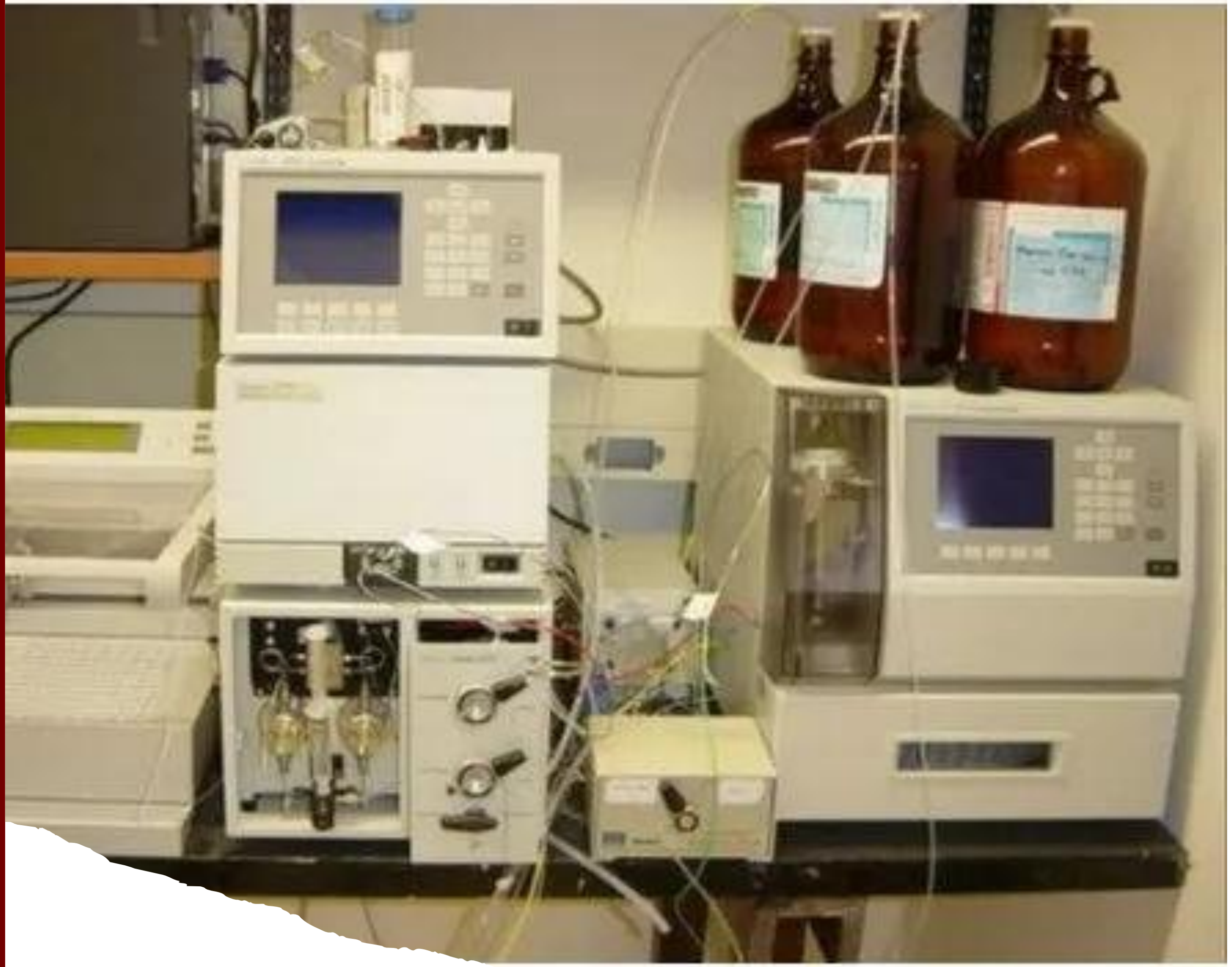

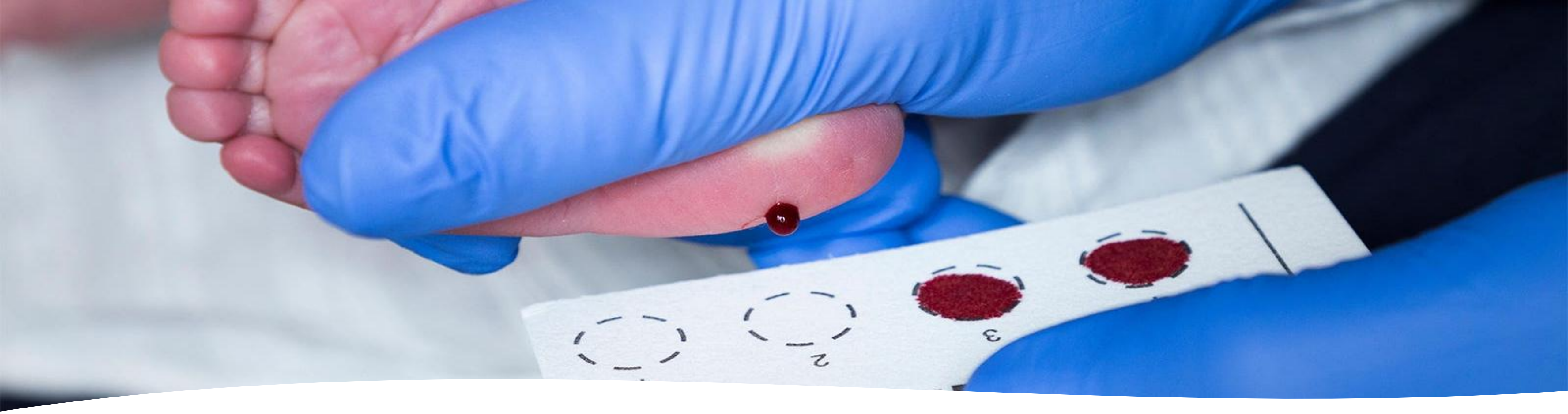

- For children less than 6 months of age: do not use CAE at alkali pH; use IEF, CE, HPLC for such young children.
- Contact National Newborn Screening Programme for referral to the laboratory with an appropriate methodology for newborns

Tests to diagnose heterozygous beta-thalassemia (trait)

Tests to diagnose heterozygous beta-thalassemia (trait)

- Tests required to help determine the presence of beta or alpha thalassemia;
- FBC (low MCV, MCH with elevated RBC count)
  - Rule out iron deficiency as the cause of microcytic hypochromic indices.
- Determine relative amounts (%) of Hb fractions (Hb A2, F, A/S ratio, etc) - using densitometry, chromatography, etc.
  - If such testing is unavailable, refer the person to a laboratory with such capability.

Rapid screening

Solubility or slide sickling test

- Use for rapid screening for the presence of Hb S in a clinically ill person in order to guide treatment decisions.
- Do not use solubility or slide sickling test in children less than 6 months of age.
- You may use these "functional" tests to confirm the identity of Hb fraction suspected of being Hb S.
- You may use solubility or slide sickling test in an emergency to rule in the presence of Hb S, and the possibility of SCD, in order to guide the clinical decision.

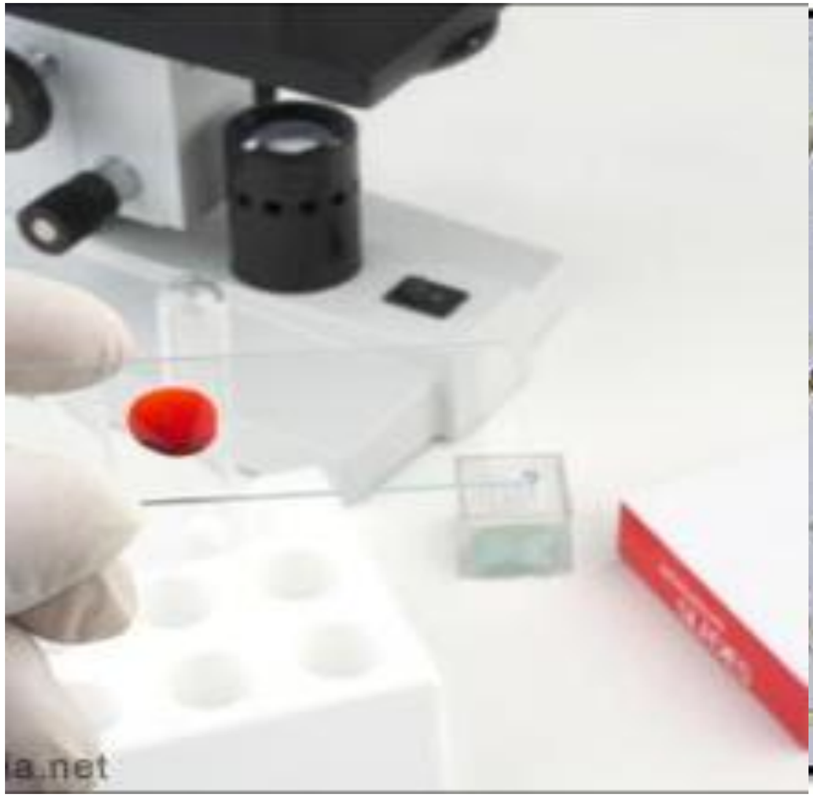
a.net
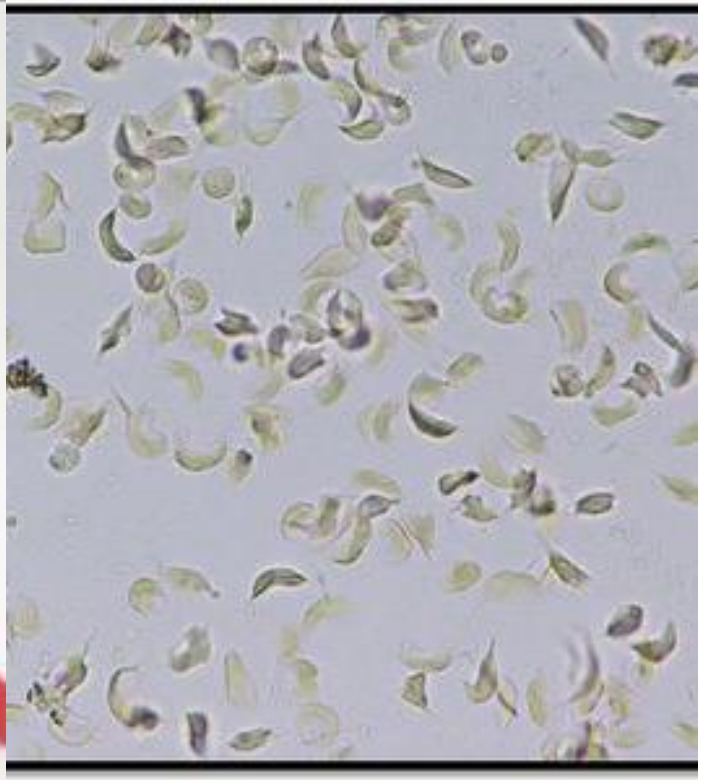
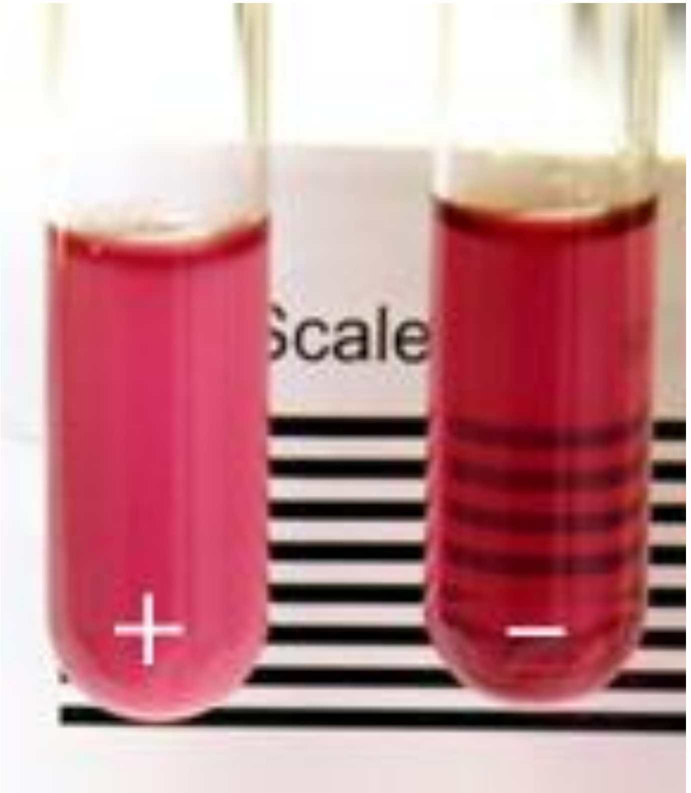
Scale
+
−

Point of Care tests for Hb S, C, A

- POC tests for rapid screening for the presence of Hb A, S, and C, or other haemoglobins, depending on the validated diagnostic ability of the specific POC test.
- You may use POC test in an emergency to rule out SCD in an undiagnosed person in order to guide clinical decisions, or in a person seeking SCD-related Hb identification for genetic counselling purposes.
  - NB: Do not use solubility, sickling, or POC tests alone to diagnose SCD, sickle cell trait, or establish Hb phenotype. Use other methods to help confirm results and true Hb phenotype or genotype

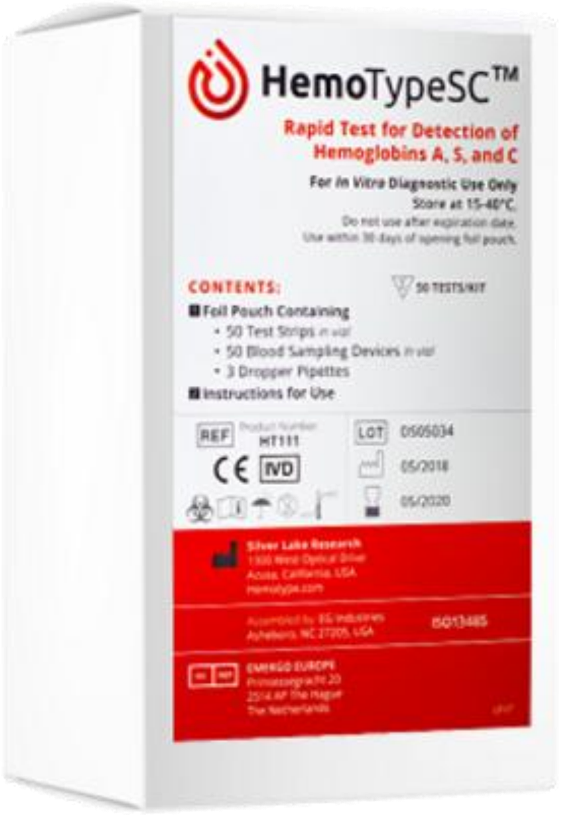
HemoTypeSC™
Rapid Test for Detection of
Hemoglobins A, S, and C
For In Vitro Diagnostic Use Only
Store at 15-40°C.
CONTENTS:
50 TESTS/KIT
Foil Pouch Containing
50 Test Strips in vial
50 Blood Sampling Devices in vial
3 Dropper Pipettes
Instructions for Use
REF
HT111
LOT
IVD
Silver Lake Research

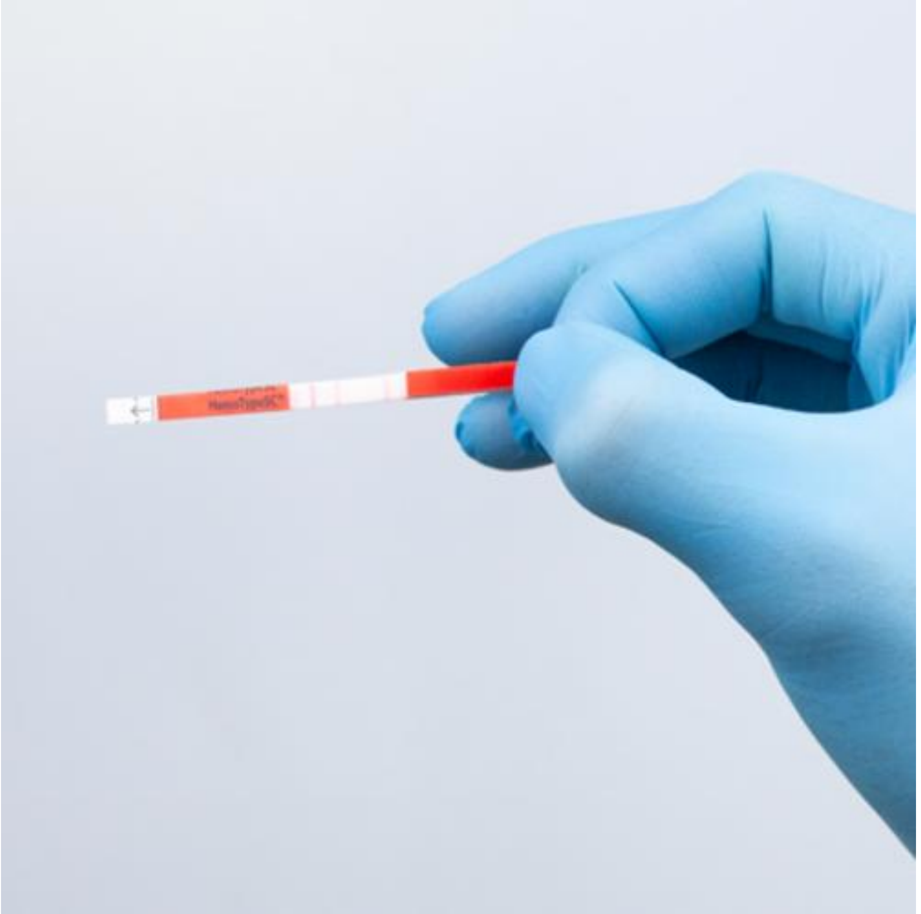

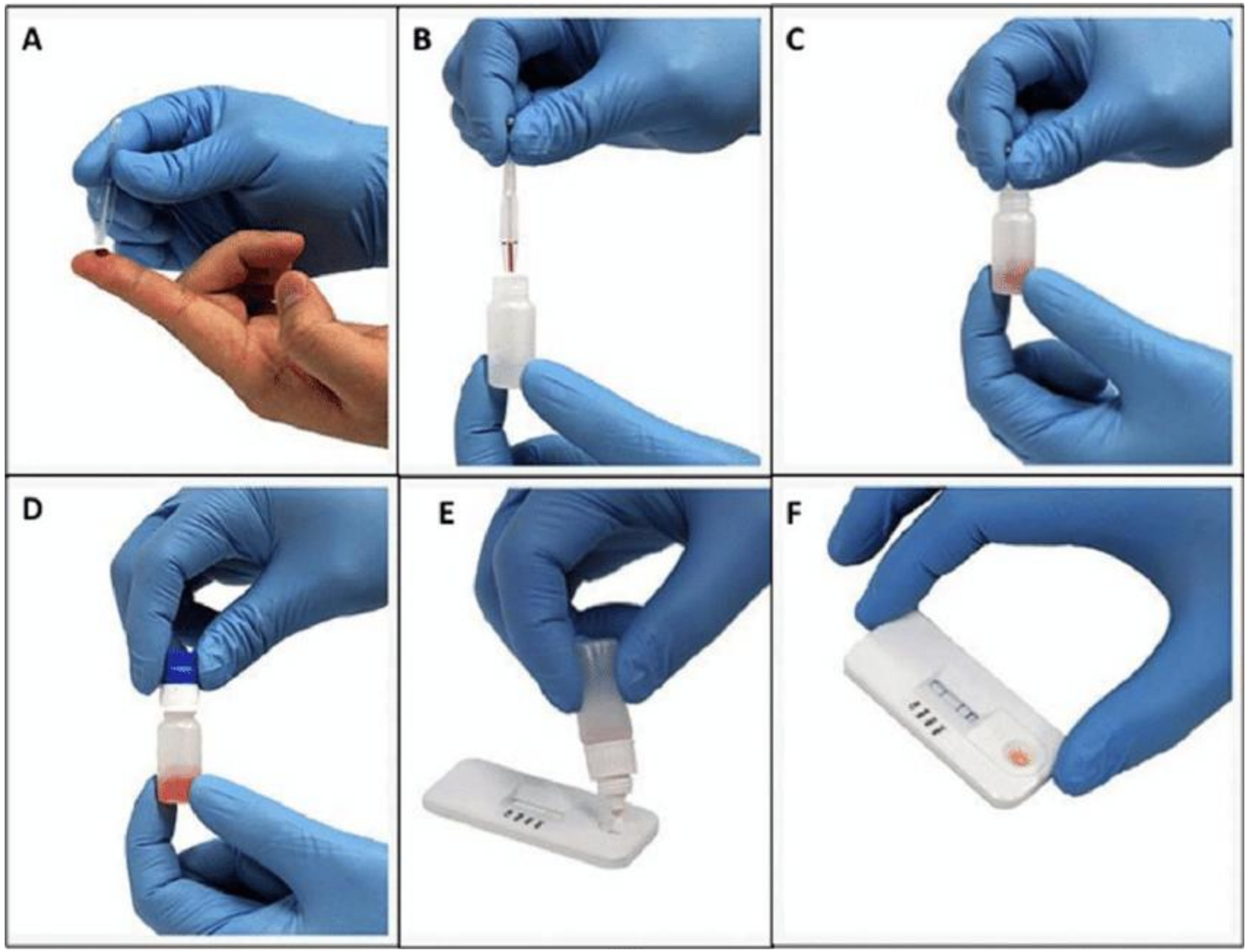
A
B
C
D
E
F

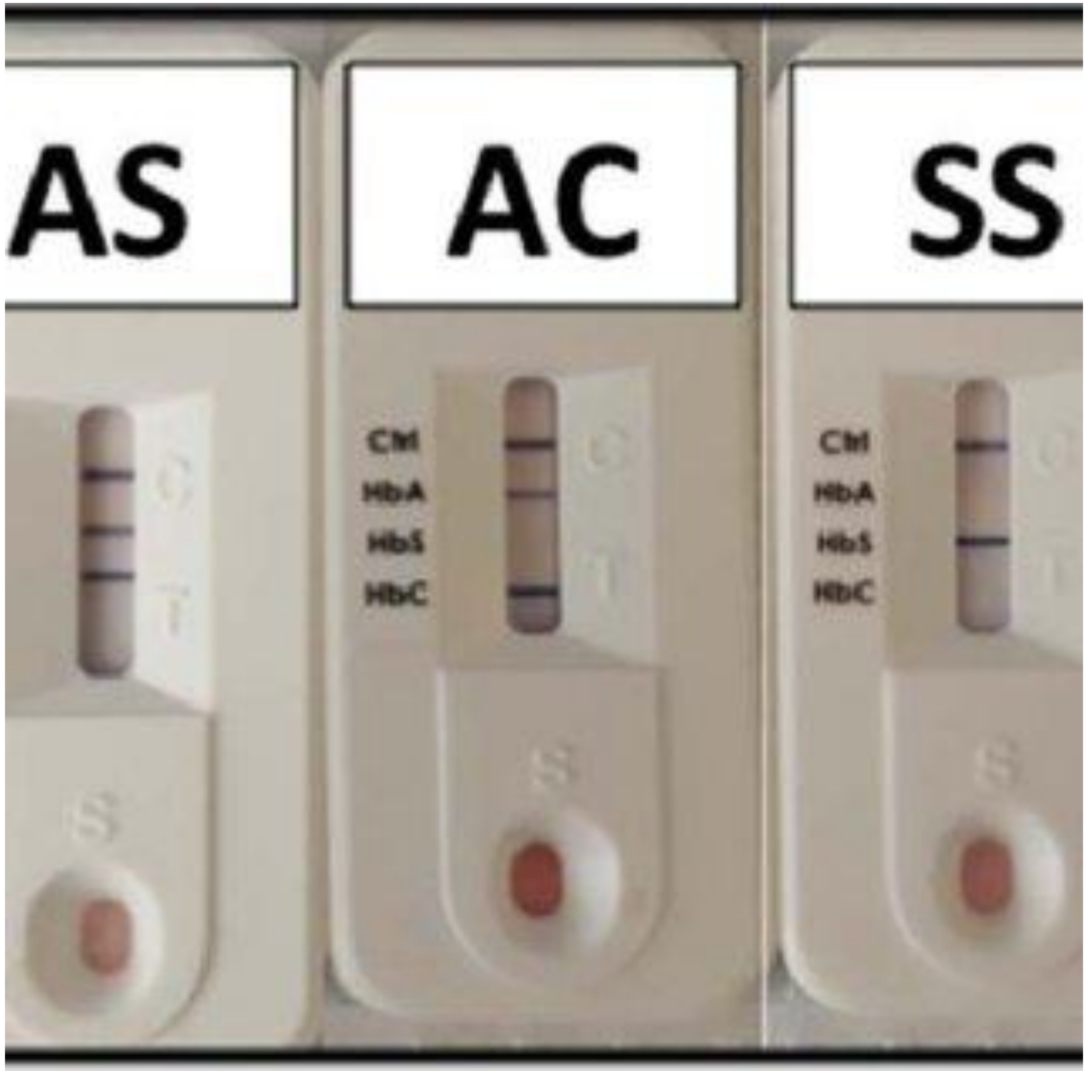
AS
AC
SS
Ctrl
HbA
HbS
HbC

DNA-Based Tests

DNA-Based Tests

- Send samples to laboratories with DNA analysis capability to confirm beta-S and other related mutations, such as
- deletional HPFH genotypes, especially when parental Hb analyses are unavailable.

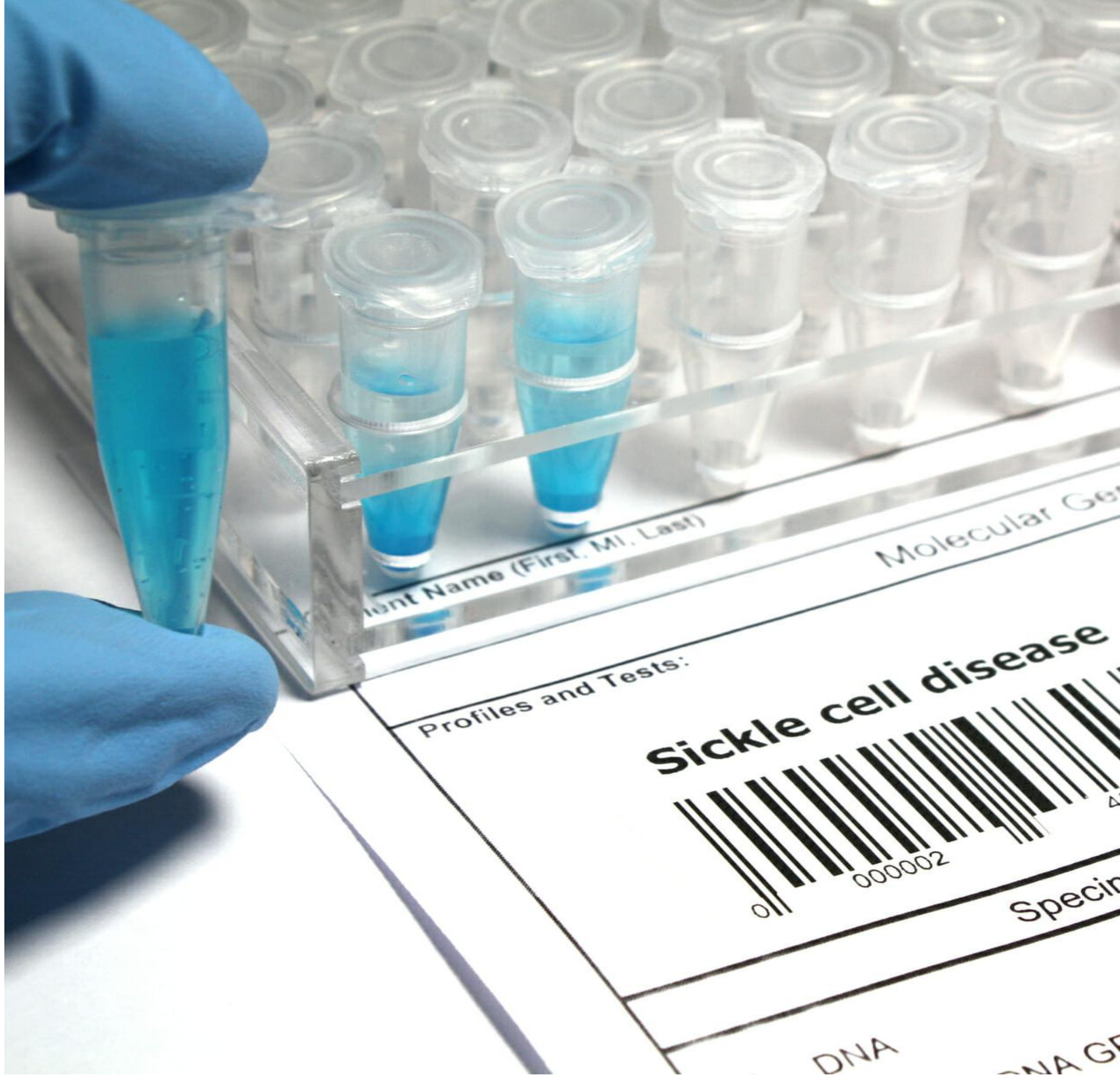

DNA tests

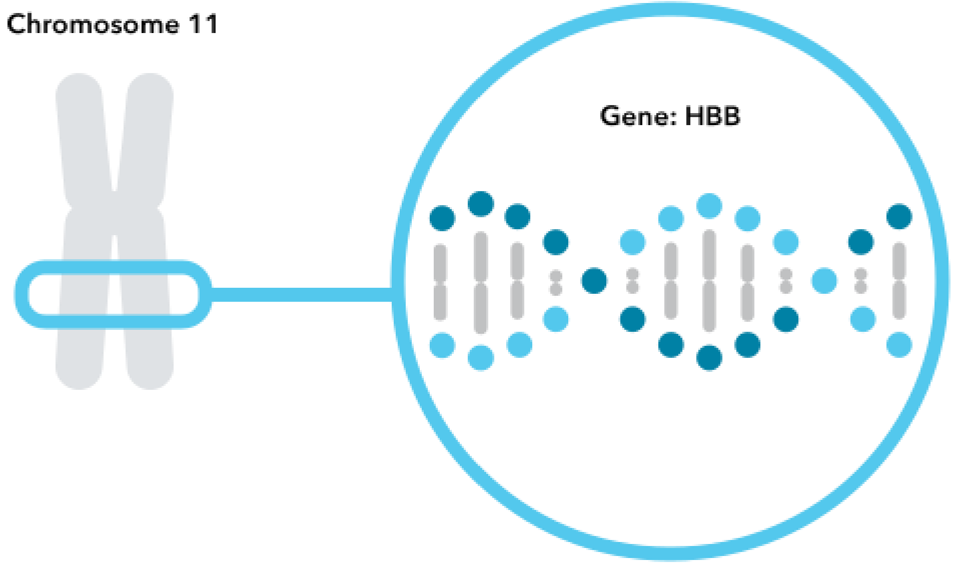
Chromosome 11
Gene: HBB

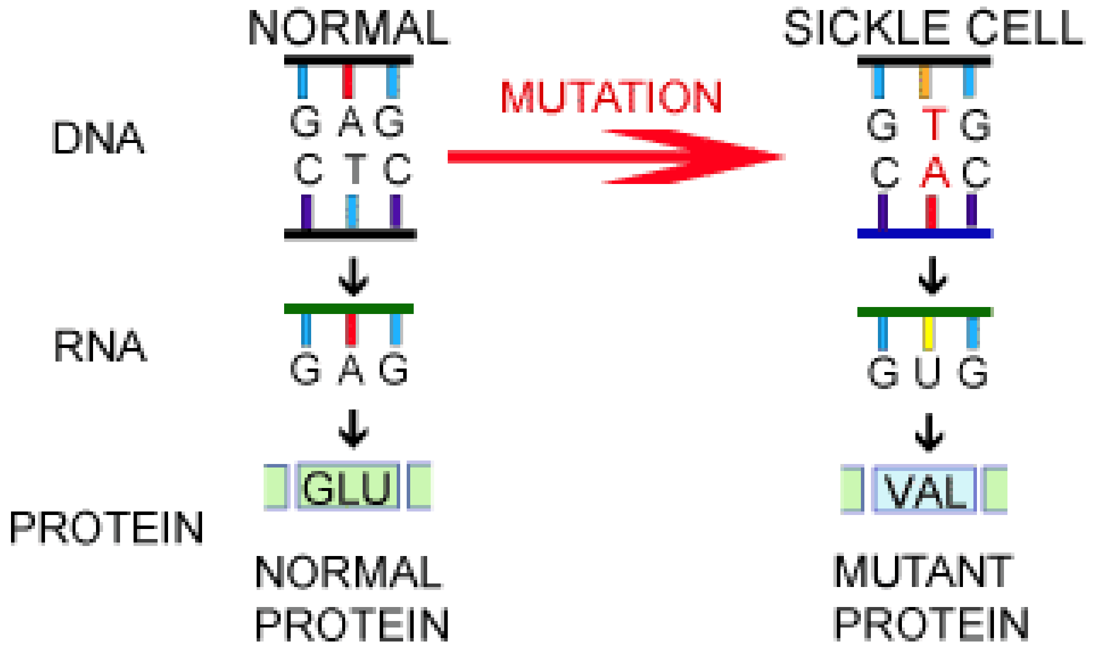
NORMAL
SICKLE CELL
MUTATION
DNA
G A G
C T C
G T G
C A C
RNA
G A G
G U G
PROTEIN
GLU
VAL
NORMAL PROTEIN
MUTANT PROTEIN

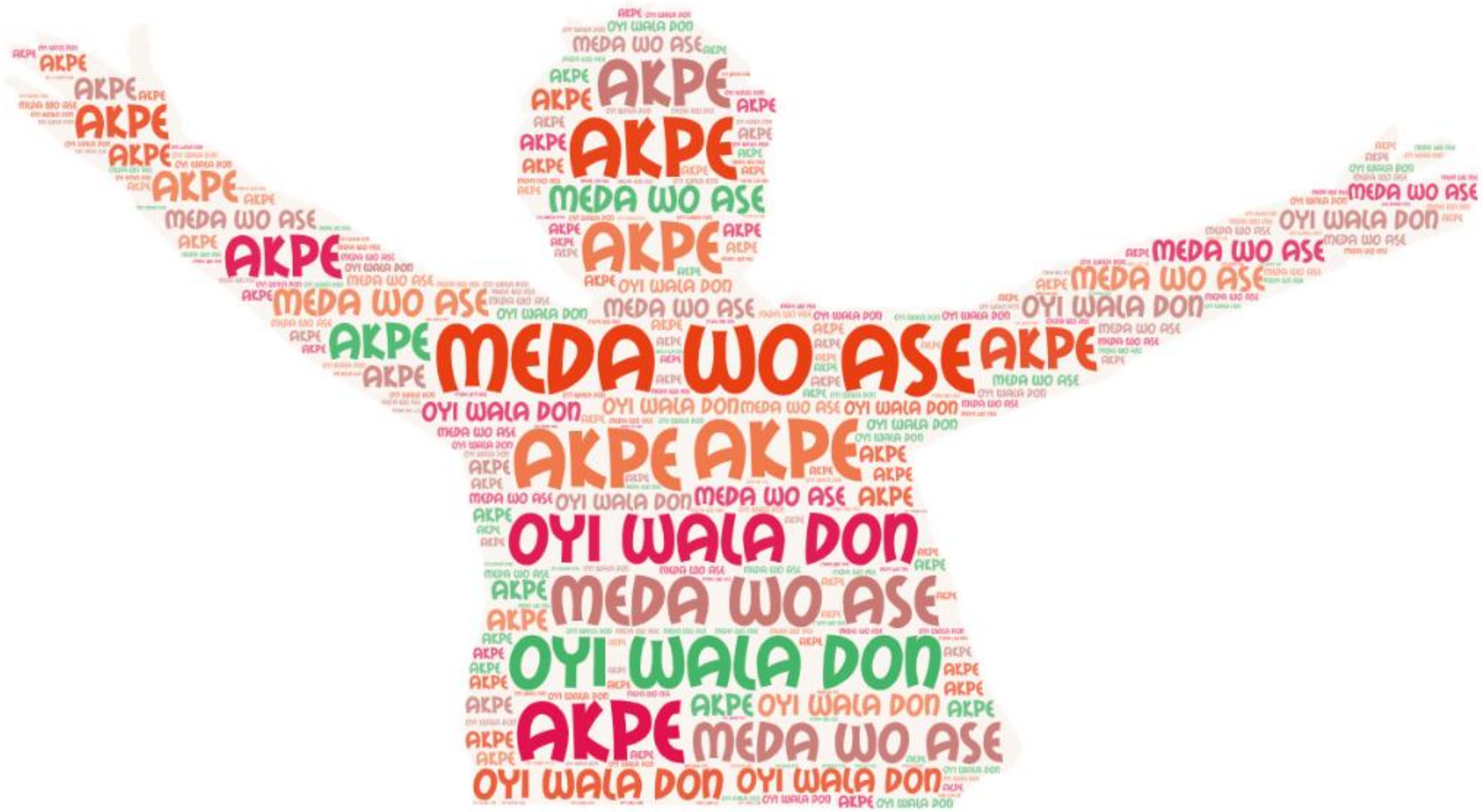
MEDA WO ASE
AKPE
OYI WALA DON

Sickle In Africa

Health maintenance in Sickle Cell Disease

Dr Vivian Paintsil

Outline

- Introduction
- Health maintenance strategies
- Screening for organ damage
- Mental health issues
- Homecare for patients

WHO DEFINITION OF HEALTH

- Health is a state of complete physical, mental and social well-being and not merely the absence of disease or infirmity

Approach to management of Chronic diseases

Promote a healthy lifestyle

Prevent premature deaths and avoid unnecessary disability

Treat effectively using latest knowledge

Provide appropriate care equitably

Introduction

- Individuals with SCD are at high risk for developing multisystem acute and chronic conditions associated with significant morbidity and mortality
- Undetected signs and symptoms can begin in early childhood
- Targeted ongoing interventions for children and adults with SCD
- Good nutrition and infection control can improve the overall outcomes for patients

Introduction

- Encourage patients to embrace the importance of health maintenance through well visits.
- Evaluate patients periodically to:
  - Establish a normal baseline
  - Identify impending problems.
  - Update immunizations
  - Maintain nutritional support
  - Provide patient / caregiver education and support

Hydration

- Due to increased risk of dehydration, children should be encouraged to drink plenty of fluids and have access to clean drinking water all the times.
- Dehydration can lead to sickling of RBCs leading to a crisis
- Encouraging adequate fluid intake is very important especially in hot climates and when undertaking strenuous exercise

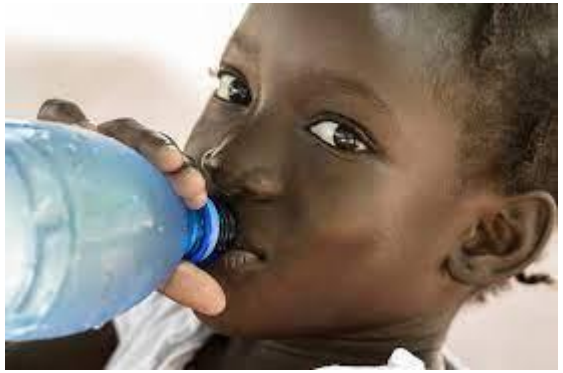

Infection prevention

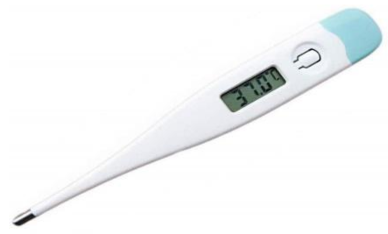

- Increased susceptibility to infections and more severe clinical course means infection prevention is crucial in maintaining well being
- Teach caregivers how to recognise fever in a child with SCD due to increased risk of overwhelming and rapidly fatal infection
- Fever > 37.5^{0}C or if thermometer not available and child feels hot, he/she should be seen at the nearest health facility in 1 hr
- Teach caregiver signs of infection – chills, lethargy, irritability, poor feeding, excessive sleepiness, vomiting etc

Infection prevention

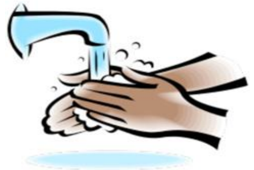

- Teach about basic hygiene and preventive measures such as hand washing with soap and use of sanitary gels, especially after use of toilet and changing of infants and toddlers, and before eating
- Enteric gram-negative organisms (salmonella, E. coli, klebsiella etc) commonly spread through faecooral contamination. (Vaccines not recommended with enteric organisms)

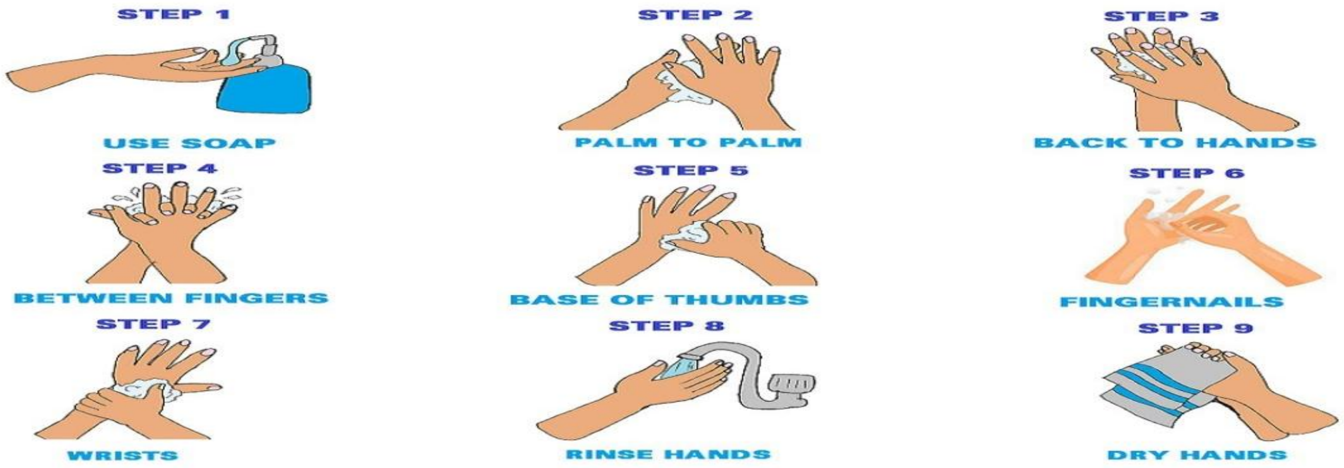

Q 1

- Which of the following is used to prevent *Strept pneumoniae* infection in SCD
  - Hand washing
  - Orelox per os
  - Amoxiclav per os
  - Penicillin per os
  - Cotrimoxazole per os

Invasive pneumococcal disease

- In the absence of preventive measures, IPD is the leading cause of death in children with SCD
- Preventative penicillin is an important intervention in routine management of children with SCD to prevent pneumococcal bacterial infection
- Start by age 2 - 3 months of age
  - 2mths – 3 yrs – 125mg bd
  - 3yrs – 5yrs - 250mg bd
- Patients allergic to penicillin - Erythromycin at equivalent dose or Clarithromycin

Q 2

- When should you stop Penicillin prophylaxis?
  - 5 years
  - After patient has gotten meningitis
  - After an episode of splenic sequestration
  - Never stop
  - None of the above

Considerations for penicillin use in patients >5yrs

- You MAY discontinue penicillin prophylaxis in those with less risky family circumstances
  - - Consider easy access to prompt and adequate medical care and family circumstances before discontinuing antibiotic prophylaxis
- Make sure child has also been fully vaccinated against pneumococcus before discontinuing Penicillin

DON'T

- Do not discontinue penicillin if;
  - Child has history of invasive pneumococcal disease (pneumonia, septicaemia, meningitis)
  - Child had surgical splenectomy
  - Immunization not up to date

Immunizations

- People with SCD should receive ALL vaccines according to schedules approved - Ghana's EPI schedule.
- Immunization of Special Interest to SCD: Vaccinate ALL people with SCD against pneumococcus, Haemophilus influenzae type b (Hib), and meningococcus.
- Ghana EPI – Pentavaccine (DPT, Hep B and HiB), Pneumovax and Meningococcal

Immunization

- Universal early childhood anti-pneumococcal vaccination has reduced further the incidence of pneumococcal bacteremia in children with SCD through herd (community) immunity.
- Anti-pneumococcal vaccination does not protect against all strains of pneumococcus.
- Penicillin prophylaxis is also not 100% protective, especially with increasing prevalence of penicillin-resistant strains in the general population.
- BOTH interventions are recommended together for each child

Malaria

- Preventing the transmission of malaria is important to reduce mortality
- Strategies include reducing contact with the vector(mosquitoes) and proper screening and treatment for suspected cases
- Advise use of Insecticide-Treated bed Net (ITN) and indoor residual spraying to help reduce malaria infection
- Parents should be advised to seek medical care **EARLY** if malaria is suspected

Folic acid supplementation

- Children with SCD have red blood cells that break down faster and also have a shorter life span
- Folic acid can help to replace red blood cells
- Daily oral dose
- Foods rich in folic acid should also be encouraged

DON’T GIVE ROUTINE IRON SUPPLEMENTATION

- Most children with SCD are likely to be iron sufficient while some may have iron overload
- Iron deficiency may occur from inadequate dietary intake or increased loss from parasitic infestations or other causes
- Iron supplementation is only used in established iron deficiency
- Caution caregivers about the use of OTC iron preparations

Q 3.

- Hydroxyurea is given to only patients with severe complications of SCD
- True
- False

Hydroxyurea

- It boost production of hemoglobin F and reduce the frequency and severity of painful episodes.
- Reduce the risk of sickle cell-related symptoms and complications.
- Treatment may be offered starting when the child is nine months old.
- Starting Dose: Children, < 18 yr: 20 mg/kg, PO, once daily;
- Adults, ≥ 18 yr: 15 mg/kg, PO.
- Patients with CKD: 5-10 mg/kg. PO.

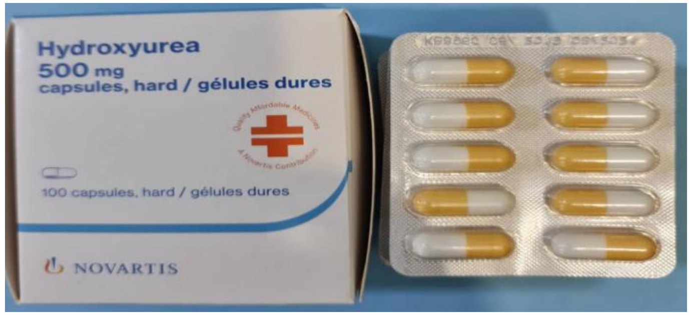

Table I. Mechanisms of action of hydroxyurea in SCD.

Increase in haemoglobin F	Interferes with HbS polymerization
Reduced red cell–endothelial interactions	Reduced adhesion under low shear flow
	Reduced VLA-4 and CD36 expression on sickle erythrocytes
	Reduced adhesion to thrombospondin and laminin
Improved red cell rheology	Improved erythrocyte hydration status
	Increased whole cell deformability
	Reduced red cell density
Myelosuppression	Reduced neutrophil counts? leads to reduced pro-inflammatory mediators
Others	Nitric oxide release, leading to vaso-dilatation and? reduced platelet activation

Patient Eligibility Criteria – Inclusion Criteria

- Hb Genotype: SCD-SS, SCD-Sb^{o} thal, SCD-SO$_{Arab}$ (On a case by case basis, a severely-affected person with SCD-SC may be offered HU therapy under a modified treatment protocol)
- Age: $\geq$ 12 months
- Clinical: None
- Social- Compliant adult patient, parent or legal guardian of the child patient

Patient Exclusion Criteria

- Ongoing chronic transfusion therapy
- Concomitant chronic illness that has the potential to increase the toxicities of HU.

Laboratory evaluation

- Samples must be collected BEFORE HU therapy is initiated and at least 12 weeks after the last RBC transfusion.
- FBC, platelet count, reticulocyte count, and WBC differential - ANC.
- Comprehensive metabolic profile, including renal and liver function tests: LDH.
- Hb F studies: Hb F%, Hb F-cell % (if available)
- Urinalysis
- Parvovirus B19 IgG (if available; and, if patient is not known to be Parvovirus B19 IgG positive)
- Serum pregnancy test for females who have achieved menarche but not menopausal.

Definition of Toxicity

- Haematologic toxicities
  - Absolute neutrophil count (ANC) < 1,000/uL
  - Absolute reticulocyte count (ARC) < 80,000/uL
  - Platelet count less than 80,000/uL
  - Fall of 20% in Hb from previous measurement or Hb<4.5g/dl
- Non-haematologic toxicities
  - Renal dysfunction – serum creatinine of 50% or more increase in serum creatinine
  - Hepatic dysfunction – ALT of more than 2 times the upper limit of normal for age

Genetic and reproductive counselling

- Counseling should be done by trained personnel.
- Adhere to the counseling procedures.
- Be friendly, empathic and ensure confidentiality.
- Provide accurate information about the disease.
- Encourage patients with SCD to have a reproductive plan (Including knowing the haemoglobinopathy status of the partner)

Female reproductive health

- Contraception – educate on the use of contraception. No contraindications for any of the usual contraceptives.
- Preconception – Stop Hydroxyurea 3 months before conception.
- Iron chelation should be stopped during pregnancy.
- Discuss with patient about the risks associated with pregnancy in SCD, both maternal and fetal risks.
- Also determine the haemoglobinopathy status of the partner.

Female reproductive health

- Pregnancy - Refer to a high risk antenatal clinic for follow up. Prescribe routine prenatal vitamins.
- Give 75mg of aspirin after 12weeks to reduce risk of pre-eclampsia.
- Delivery - Vaginal delivery is preferred, unless there is indication for caesarian section.
- Postpartum - Consider use of venous thromboembolism prophylaxis for patients with additional risk factors for VTE e.g., previous history of VTE, caesarian section or immobility.
- Assess and manage neonatal opioid dependency and withdrawal all infants with history of in utero opioid exposure.

Male reproductive health

- Encourage men with SCD to have reproductive life plan and to decide whether to have children or not and the implications.
- Counsel and educate men with SCD about heritability of SCD.
- Provide pre-marital counseling to a man with SCD together with his fiancée

Screening for organ damage

- CNS complications – TCDs from 2-16yrs, MRI/MRA subsequently when indicated
- Eye changes (Retinopathy) – Fundoscopy from age 10yrs
- Heart and lung diseases – not routinely required unless patient symptomatic
- Kidney – Urine for proteinuria and microalbuminaemia from age 10yrs. If negative, repeat annually. Otherwise do urine albumin-creatinine ratio and refer to nephrologist if abnormal

Mental health

- Require additional psychosocial and mental health supports
- Problems
  - Inappropriate coping strategies
  - Reduced quality of life
  - Anxiety and depression
  - Neurocognitive impairment

Healthy diet

- Undernutrition causes increased morbidity and mortality and contributes to poor clinical outcome and severity of disease
- Increased metabolic rate results in increased caloric requirements
- A balanced diet is needed that provides plenty of calories, protein, vitamin and minerals.
- Infants < 6mths – exclusive breastfeeding
- 6mths – 2yrs – continued breastfeeding with introduction of nutritious high calorie semi-solid to solid foods
- > 2yrs & adolescents – should have a balanced diet

Home care

- Educate the caregiver about danger signs and when to take the child to the hospital
- Educate caregivers about the importance of screening for the various organ damage and when it has to be scheduled.
- Educate about the importance of taking the prophylactic medication
- Early reporting to the health facility

Managing SCD pain at home

- Administration of analgesics preferably paracetamol regularly or use analgesics written by your HCW.
- Give extra fluids
- Warm bath
- Use warm moist towels for massage
- Quiet play and diversional therapy
- Seek medical help if these doesn’t work

Visit frequency to the SCD clinic

- Encourage regular visits to the SCD clinic. Reduce missed appointments
- More frequent visits might be required for patients with frequent complications or for therapeutic monitoring (HU)

AGE	FREQUENCY
First 24 months of life	Every 2 months
2yrs – 12 years	3 mths – 4 mths
> 12 years	4mths – 6 mths

ACTIVITIES AT CLINIC
Anthropometry/ vital signs
History of illness and wellbeing
Review of medications and immunizations
Detailed physical examination
Continued family education and support
Laboratory investigations or imaging as required

Q 4

- What psychosocial interventions can you implement to support patients with SCD?

Education and psychosocial counselling

- Educate all children from school going age with their parents/caregivers and adults routinely about SCD at each visit: utilize available materials e.g., brochures, videos, and apps.
- Prompt patients and parents to talk about stigma and their experience with how their communities and others relate to them, dispel myths.
- Educate parents/caregivers about importance of schooling, encourage their children to attend school when they are well and to keep a record of the number of school days lost.

Education and psychosocial counselling

- Encourage adults to find employment (paid or voluntary).
- Encourage patients to participate in social activities and not feel isolated.
- Encourage participation in support groups where available.
- Encourage self-management and reinforce health maintenance.
- Ensure understanding of the importance of adherence to medical advice and treatment.
- Offer psychoeducational sessions: individual and/or group or family sessions as appropriate

CONCLUSION

- Health maintenance is a major intervention when managing patients with SCD
- Diligently following the required steps improves the general well-being of the patients.
- Education of the caregivers is an important step in ensuring that patients adhere to a good health maintenance schedule

References

- SPARCo Standards of Care for Sickle Cell Disease in sub–Saharan Africa. 1st Ed. 2023
- Evidence based management of Sickle cell disease: an expert panel report 2014
- Health maintenance for sickle cell disease; https://nyulangone.org/conditions/sickle-cell-disease-in-children/treatments/health-maintenance-for-sickle-cell-disease
- Draft Ghana guidelines for management of SCD
- NHS Screening programmes. Sickle cell and thalassaemia. A parents guide to managing Sickle Cell Disease

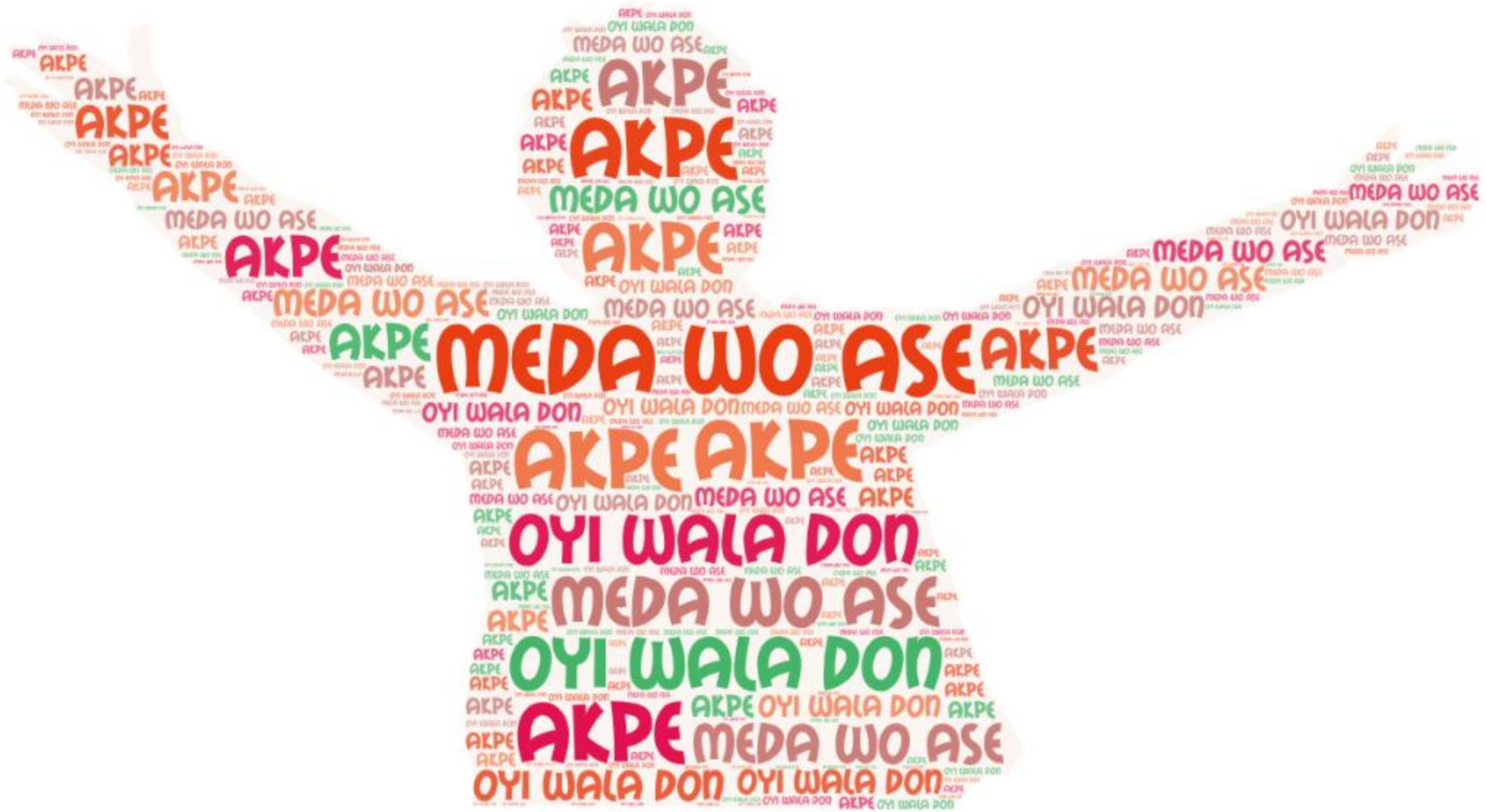
MEDA WO ASE
AKPE
OYI WALA DON

ACUTE PAIN IN SICKLE CELL DISEASE

OUTLINE

- Pretest
- Objectives
- Case presentation
- Pathophysiology of Acute Pain
- Management

Pretest

1. The following objective indicators can be reliably used to assess acute pain?

a. Temperature >/= 38.3

b. Heart rate greater than 100b/m

c. Blood oxygen saturation >92%

d. All the above

e. None of the above

Pretest Cont'd

2. 9 years old known patient with Sickle Cell disease presents to the ED with a 3-day history of pain. She was crying and holding her left knee in pain.

Discuss management?

Iceberg Theory of Pain SCD

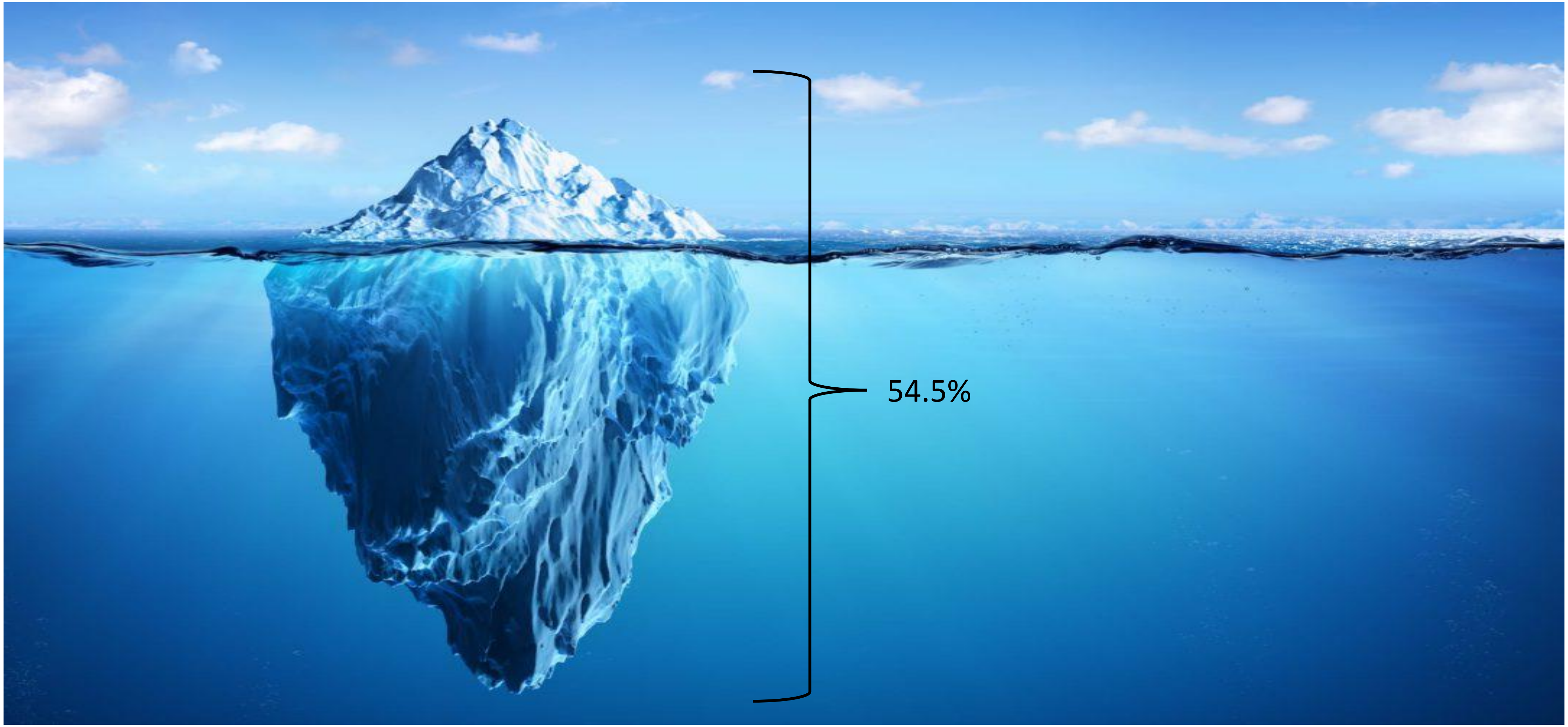

Objectives

- Different forms of pain in SCD

- Pathophysiology behind acute pain

- Approach to acute pain in SCD

Introduction

- Pain is defined as an unpleasant sensory and emotional experience, associated with, or resembling that associated with, actual or tissue damage.(IASP)

- Pain is the hallmark symptom of sickle cell disease(SCD) and driver of the majority of interactions with the healthcare system.

- At KATH, SCD admissions makes up 6.5% of the total admissions in the pediatric unit with VOPE being the leading cause of these admissions

Types of pain in SCD

- **Acute pain** (main topic for discussion)
  - Acute intermittent pain
  - Acute on chronic pain

- **Chronic pain**

SICKLE CELL PAIN

	Acute pain	Chronic Pain
Onset	Sudden/abrupt	Insidious/gradual/on-going
Mood	Anxiety	Depression
Duration	Hours –weeks (short term)	Months to years(long term)
Treatment	Per episode	Continous

Pathophysiology of Acute Pain

- Acute pain in patients with SCD is caused by Ischaemic injury resulting from occlusion of microvascular bed by sickled celled cells.

- Sickled cells are rigid, brittle, sticky and are unable to pass through smaller vessels easily.

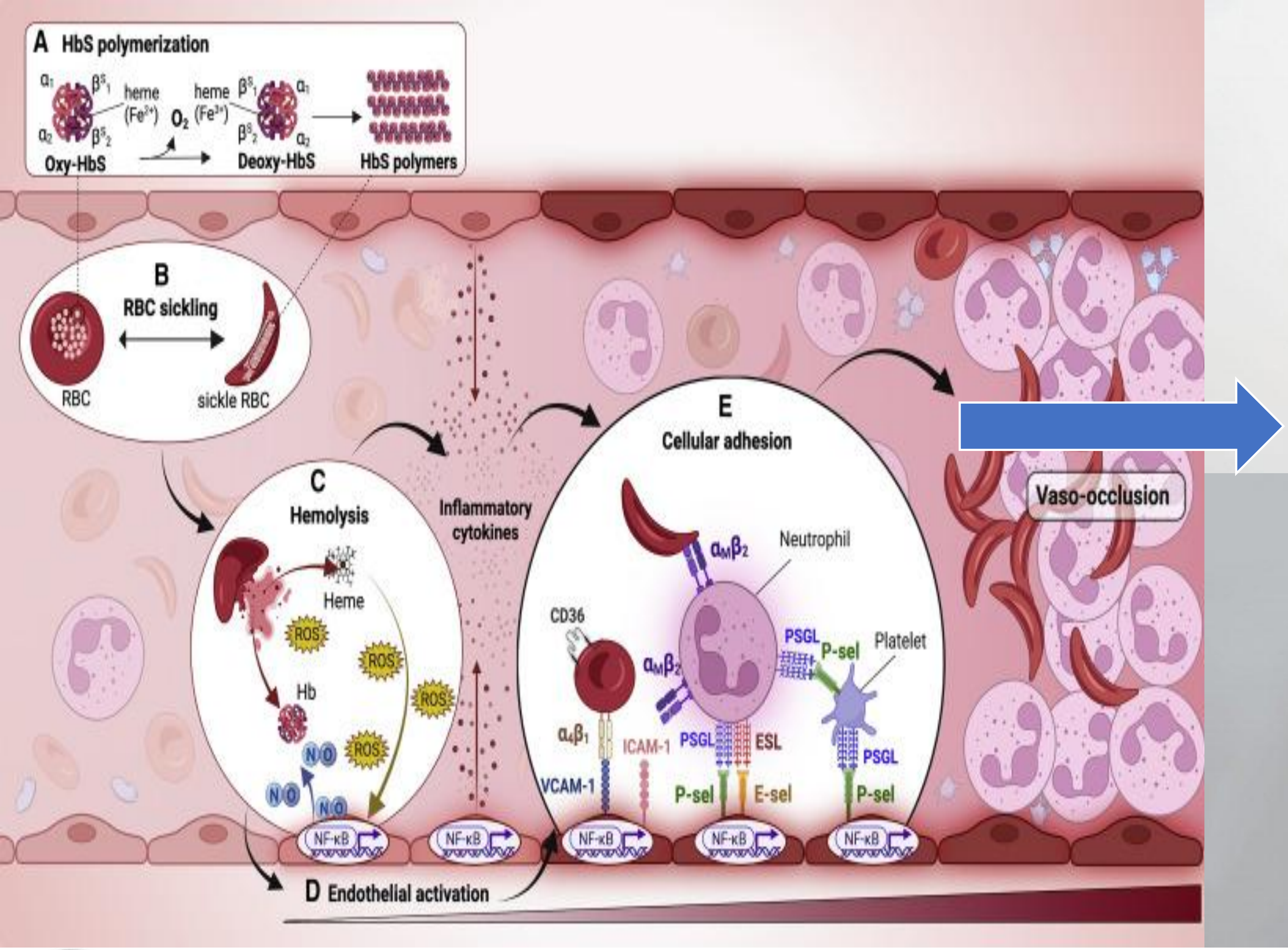
A HbS polymerization
heme (Fe2+)
O2
heme (Fe3+)
Oxy-HbS
Deoxy-HbS
HbS polymers
B RBC sickling
RBC
sickle RBC
C Hemolysis
Heme
ROS
Hb
NO
Inflammatory cytokines
E Cellular adhesion
Neutrophil
CD36
PSGL
P-sel
Platelet
ICAM-1
ESL
VCAM-1
E-sel
NF-κB
D Endothelial activation
Vaso-occlusion

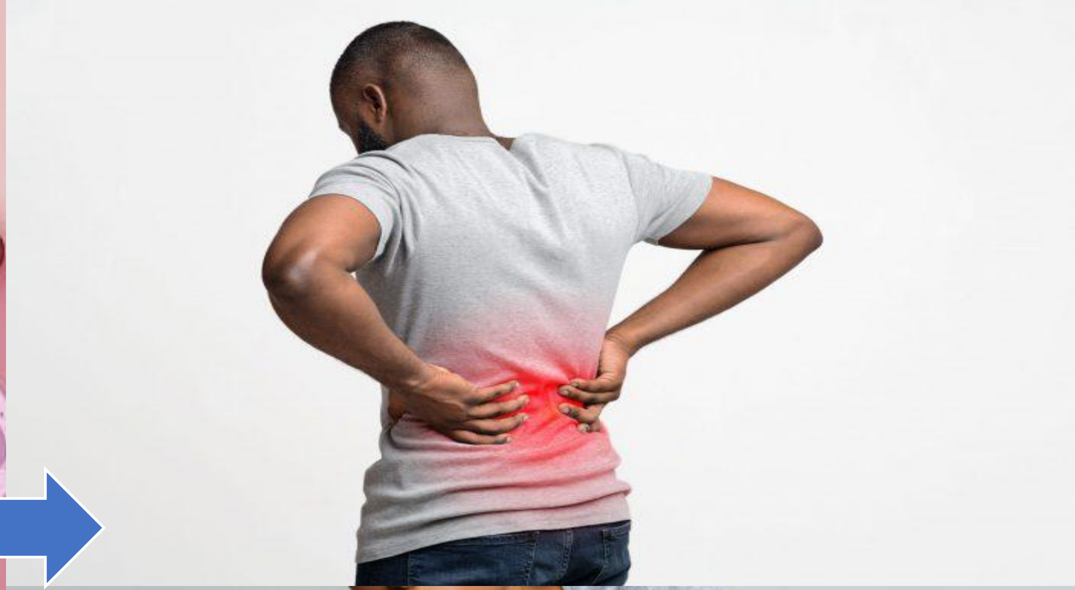

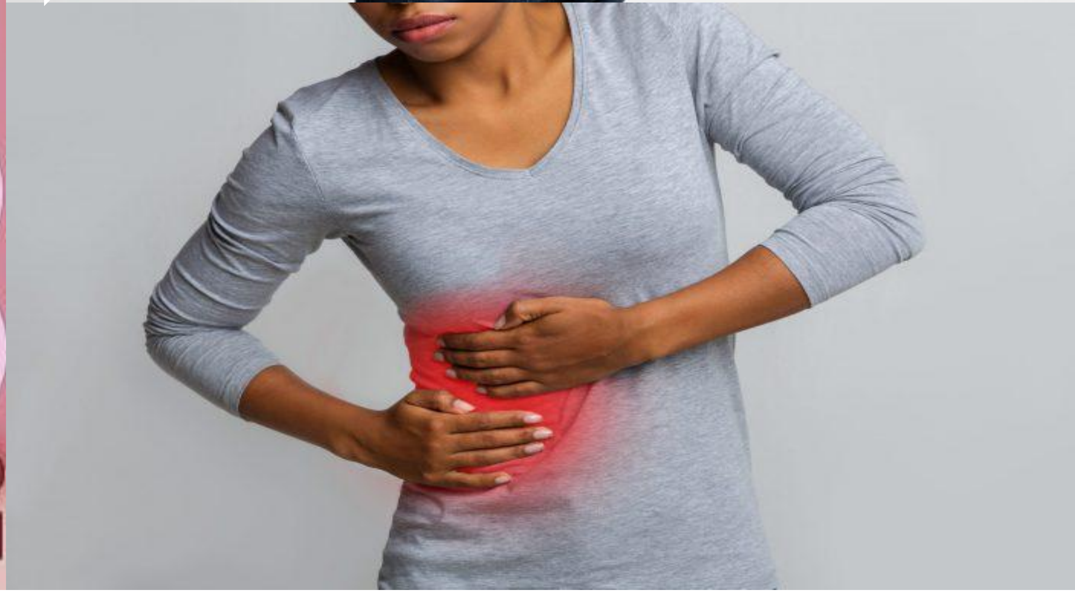

Triggers of vaso-occlusion

- Patient factors: hypoxia, infection, fever, acidosis, dehydration, pregnancy, menstruation, obstructive sleep apnea, pain, anxiety, depression, alcohol consumption, physical exhaustion

- Environmental factors: exposure to temperature extremes(cold or heat), high wind speed, and humidity

*Acute pain often develops without an apparent event and with little warning.

Presentation

- There are no objective indicators that can be used to reliably indicate the presence &/or severity of a VOPE
- Pain is subjective and the gold standard is a self report by patient.
- The following objective indicators can be reliably used in the diagnosis of acute SCD pain:
  - Lab work ✗
  - Radiographic findings ✗
  - Vital signs ✗

Presentation cont.

- VOPE's can occur in any part of the body but frequently affected areas are the extremities(leg and arms) long bones, back, head, chest, and abdomen.
- They may present with/without fever

Approach to Managing Acute SCD Pain

Steps in the Approach to Acute Pain

- Assessment of pain
- Intervention (Pharmacological and non-pharmacologcal)
- Investigate and treat underlying triggers
- Exclude other differentials
- Re-assess after intervention
- Supportive care (Nutrition and Hydration)

ASSESSMENT OF PAIN

- Site, character, duration, aggravating and relieving factors. Previous pain experience and analgesic treatment.
- Effect on activity, play and feeding
- Physical examination
- Age appropriate tool to score
- Classify pain
- Reassess 30-60 mins

PAIN RATING SCALES

Wong-Baker FACES Pain Rating Scale

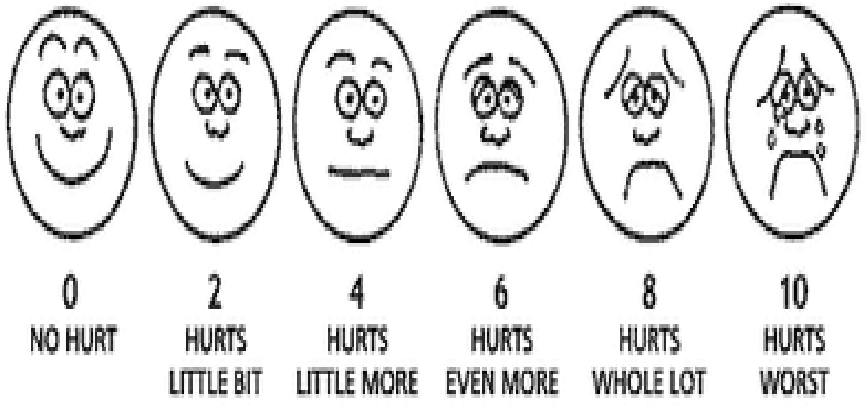

From Wong D.L., Hockenberry-Eaton M., Wilson D., Winkelstein M.L., Schwartz P.: Wong's Essentials of Pediatric Nursing, ed. 6, St. Louis, 2001, p. 1301. Copyrighted by Mosby, Inc. Reprinted by permission.

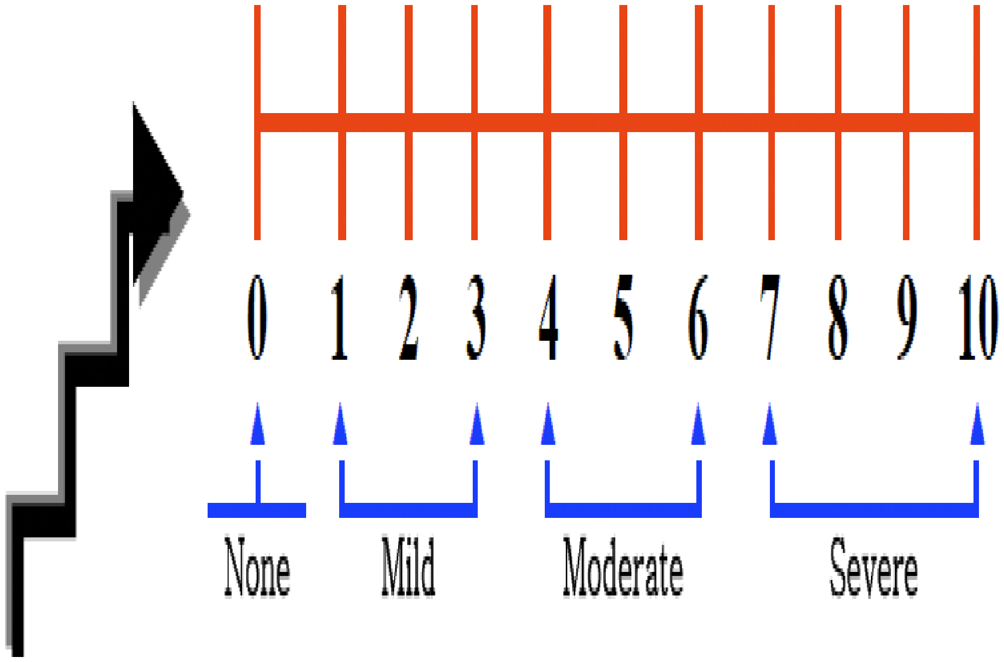

FLACC SCALE

DATE/TIME						
**Face**<br>0 - No particular expression or smile<br>1 - Occasional grimace or frown, withdrawn, disinterested<br>2 - Frequent to constant quivering chin, clenched jaw						
**Legs**<br>0 – Normal position or relaxed<br>1 – Uneasy, restless, tense<br>2 – Kicking, or legs drawn up						
**Activity**<br>**0** – Lying quietly, normal position, moves easily<br>**1** – Squirming, shifting back and forth, tense<br>**2** – Arched, rigid or jerking						
**Cry**<br>**0** – No cry (awake or asleep)<br>**1** – Moans or whimpers; occasional complaint<br>**2** - Crying steadily, screams or sobs, frequent complaints						
**Consolability**<br>**0** – Content, relaxed<br>**1** – Reassured by occasional touching, hugging or being talked to, distractible<br>**2** – Difficult to console or comfort						
**TOTAL SCORE**						

ACUTE VASO-OCCLUSIVE PAIN EPISODE MGT

- Assess the pain and classify (mild, moderate or severe)

↓

- Pharmacologic - 2 STEP STRATEGY for children and 3-step strategy for adults. (morphine is given by the clock, by mouth and by individual

- Non pharmacologic: massaging, application of heat, distraction, Virtual reality, audio-visuals

- Identify and treat underlying cause if any

2 STEP STRATEGY for Children

- Key : Treament should be individualized, employing the strongest analgesic (for that intensity of pain) as initial therapy and later gradually toning down

- Mild : Non Opoid option +/- non-Pharmacological Adjuncts

Paracetamol:10-15 mg/kg/dose Q4-6hrs, max dose 75mg/kg/day or 4 grams

Ibuprofen: 5-10mg/kg/dose Q6-8hrs, max 40mg/kg/day or 2400mg/day

- Moderate to severe: Opoid +/- pharmacological adjunct + non pharmacological adjunct

Morphine: PO: 0.2-0.4 mg/kg/dose, IV: 0.1- 0.2 mg/kg/dose

3 STEP STRATEGY for Adults

- Mild
- Moderate
- severe

Assessment and supportive care

- This should be done every 30-60 min and analgeisics optimized till control is attain
- Also assess for side effects of analgesics
- Hydration -oral and/or IV infusion at maintenance. Use D/S or N/S.
- Start physiotherapy including incentive spirometry other complications.
- Investigate if atypical features are present and manage accordingly*
- Discharge patient when pain is improving on lowest effective oral analgesia and schedule for review on OPD basis

Other painful events in SCD

- Vaso-occlusive pain episodes, including dactylitis (Hand-Foot syndrome)
- Acute abdominal pain(Mesenteric Crisis),(Acute Cholecystitis-inclusive)
- Acute chest syndrome
- Priapism
- Important differential diagnosis: Acute abdomen, osteomyelitis, septic arthritis

CASE

9 years old known patient with Sickle Cell disease presents to the ED with a 3-day history of pain. She was crying and holding her left knee in pain.

Discuss management?

Conclusion

- Acute pain management in SCDx is not a one size fit all affair, it should be individualized.
- Pain is subjective, there are currently no objective ways of assessing pain.
- Lack of proper knowledge of analgesics, underdosing, incorrect timing of medications and unwarranted fear of addiction are barriers to effective acute pain management in SCDx.
- Effective pain management is key to improving the HRQoL of patients with SCD.

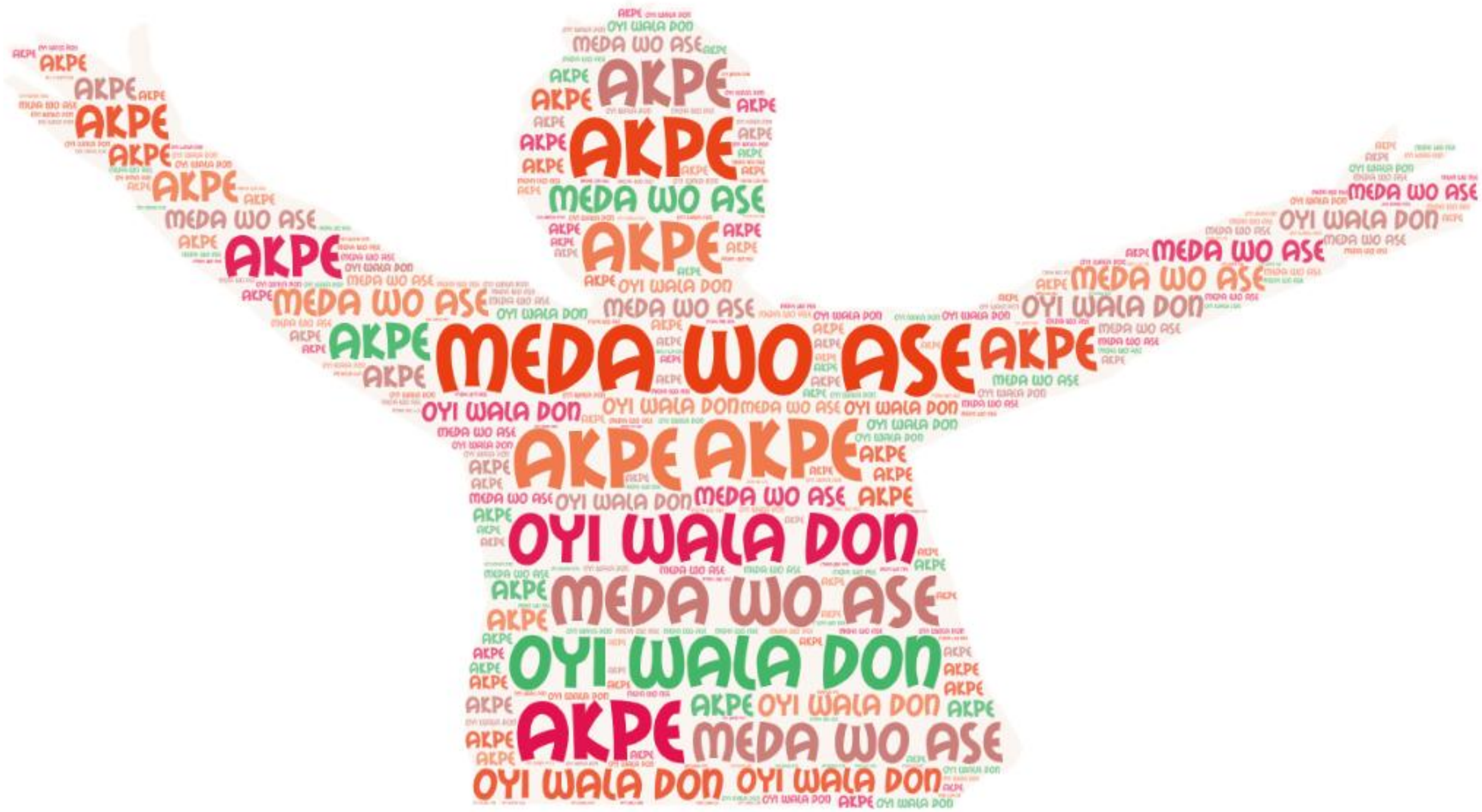
MEDA WO ASE
AKPE
OYI WALA DON

ACUTE CHEST SYNDROME(ACS)

OBJECTIVES

- Definition of Acute chest syndrome (ACS)
- To identify the causes / risk factors of ACS
- To understand the pathophysiology of ACS
- To recognize elements that are important in appropriate management
- Prevention

ACUTE CHEST SYNDROME

- ACS is defined as an acute illness characterised by fever and /or respiratory symptoms accompanied by a new pulmonary infiltrate on chest xray

ACUTE CHEST SYNDROME

- ACS occurs in all genotypes of SCD
- It occurs in ~45% of patients, and recurs in ~80%
- 2nd most common cause of hospitalization in SCD patients
- Is associated with high intensive care (ICU)admission
- Accounts for one- fourth of SCD- related deaths
- Mortality: ↑4x in adults than children
- It's a common cause of maternal mortality

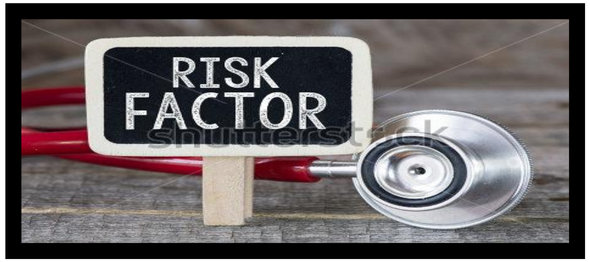

ACUTE CHEST SYNDROME

- Younger age
- HbSS + HbSB°- thal genotype
- Cold weather
- Fever
- VOC
- Use of Opioid analgesics

- Surgery (x'lly abdominal)
- Avascular necrosis of bone
- Previous pulmonary events
- High Hb levels
- High steady-state WBC
- Low HbF concentration

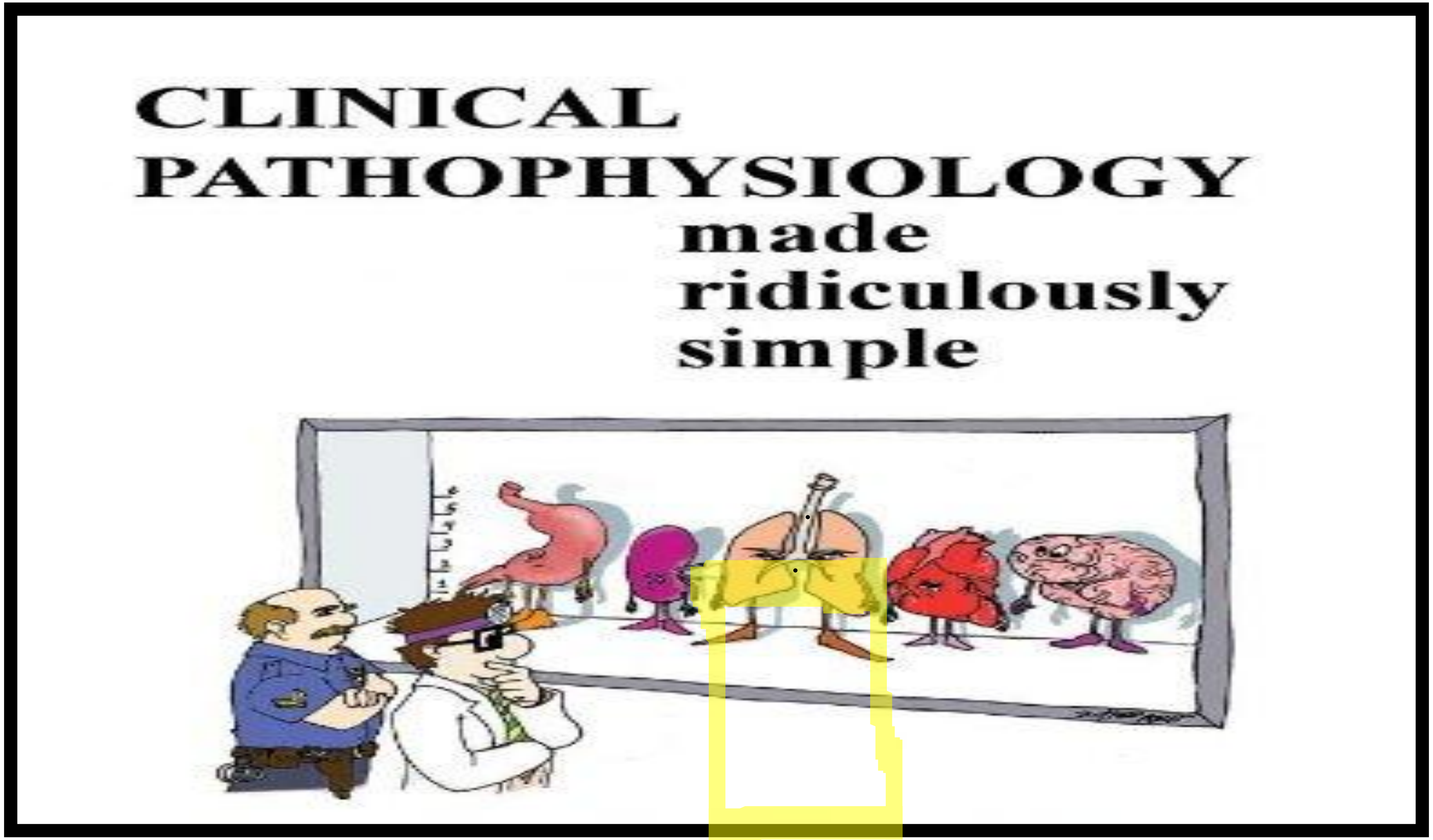
CLINICAL
PATHOPHYSIOLOGY
made
ridiculously
simple

ACS: Pathophysiology

- The aetiopathogenesis of ACS is incompletely understood.
- Not identifiable in almost 46% of cases
- May result from either one or a combination of several processes;

ACS: Pathophysiology

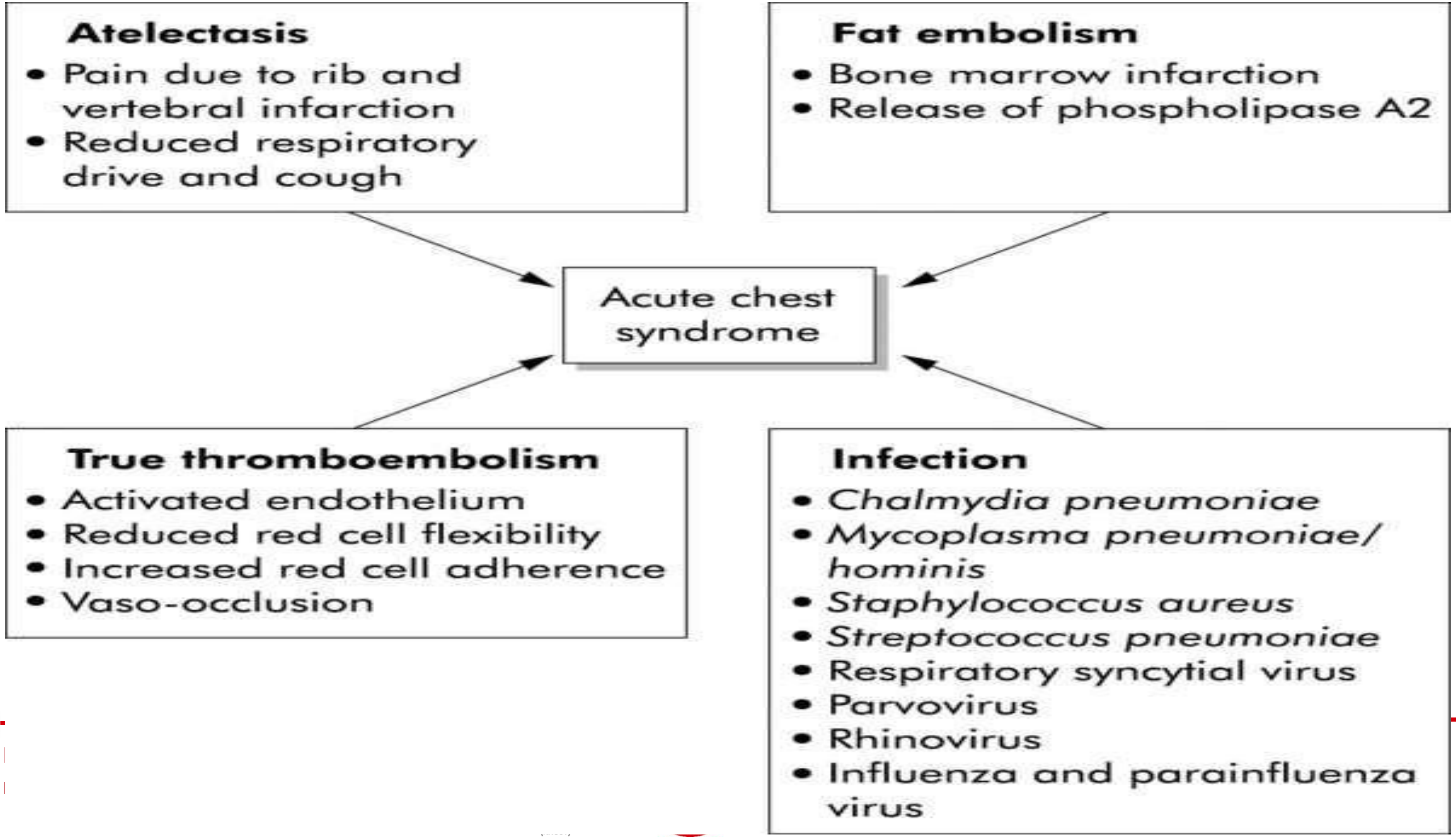

Sickle In Africa

CLINICAL FEATURES

- Clinical features of ACS may not be evident at time of admission.
- Nearly half of patients present initially with a painful VOC → ACS
  - ACS will often develop 24 to 72 hrs after the onset of severe pain.
  - All patients admitted with painful crisis should be considered to be potentially in the prodromal phase of ACS.
- Additionally, ACS can develop post-operatively, especially following abdominal surgery

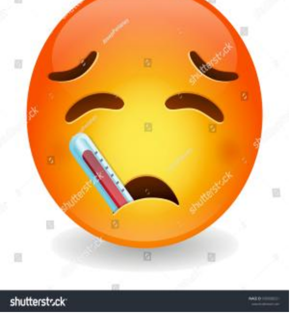

Symptoms & signs

- Hypoxia
- Fever
- Tachypnoea
- Tachycardia
- Wheeze
- Chest signs include dullness to percussion, reduced air entry, crepitations, bronchial breath sounds, rhonchi and pleural rubs
- Pleural effusions

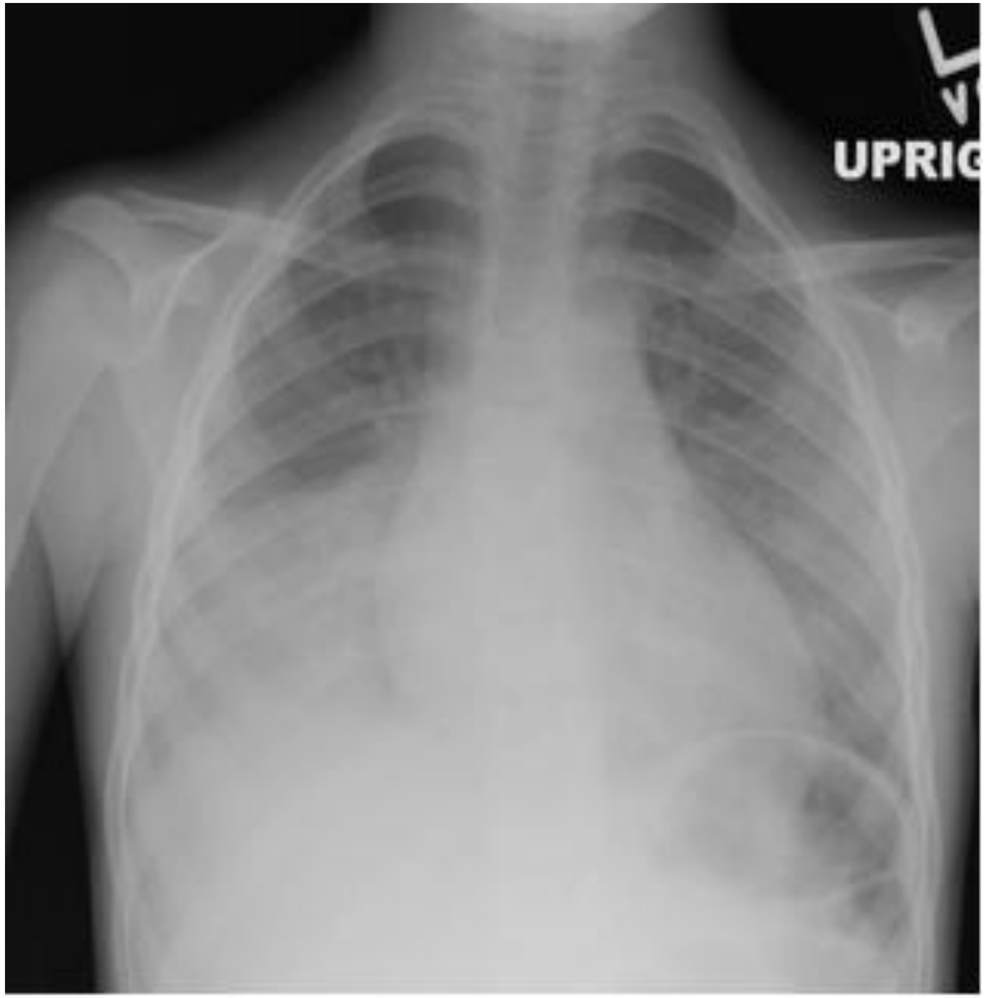

In early stages chest examination may be normal

	Children	Adults
Fever	+++	++
Cough	++	++
Chest pain	+	++
Dyspnoea	+	++
Tachypnoea	+	+
Wheezing	+	+/—
Intercostal recession/nasal flaring	+	+/—
Skeletal pain	+	++
Hypoxia	++	+++
Haemoptysis	+/—	+

+++ frequent (>80%), ++ common (50–79), +less common (10–49%), +/— infrequent (<10%).

INVESTIGATIONS

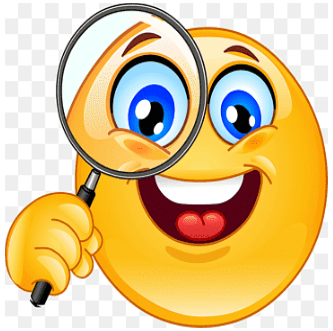

- Haematology labs:
  - FBC + Film comment
  - Reticulocyte count
  - blood for grouping & crossmatch.
- Biochemistry tests:
  - BUE & Cr
  - LFTs
  - LDH
  - Arterial blood gases analysis should be performed in adults with $SpO_2 \leq 94\%$ on room air.
- Microbiology labs:
  - Blood cultures
  - sputum for microscopy and culture
  - Urine RE & C/S
- Radiology
  - Plain chest xray
  - CT pulmonary angiogram (CTPA) is recommended if there is a high clinical suspicion of pulmonary embolism .

Chest xray showing rapid progression of ACS

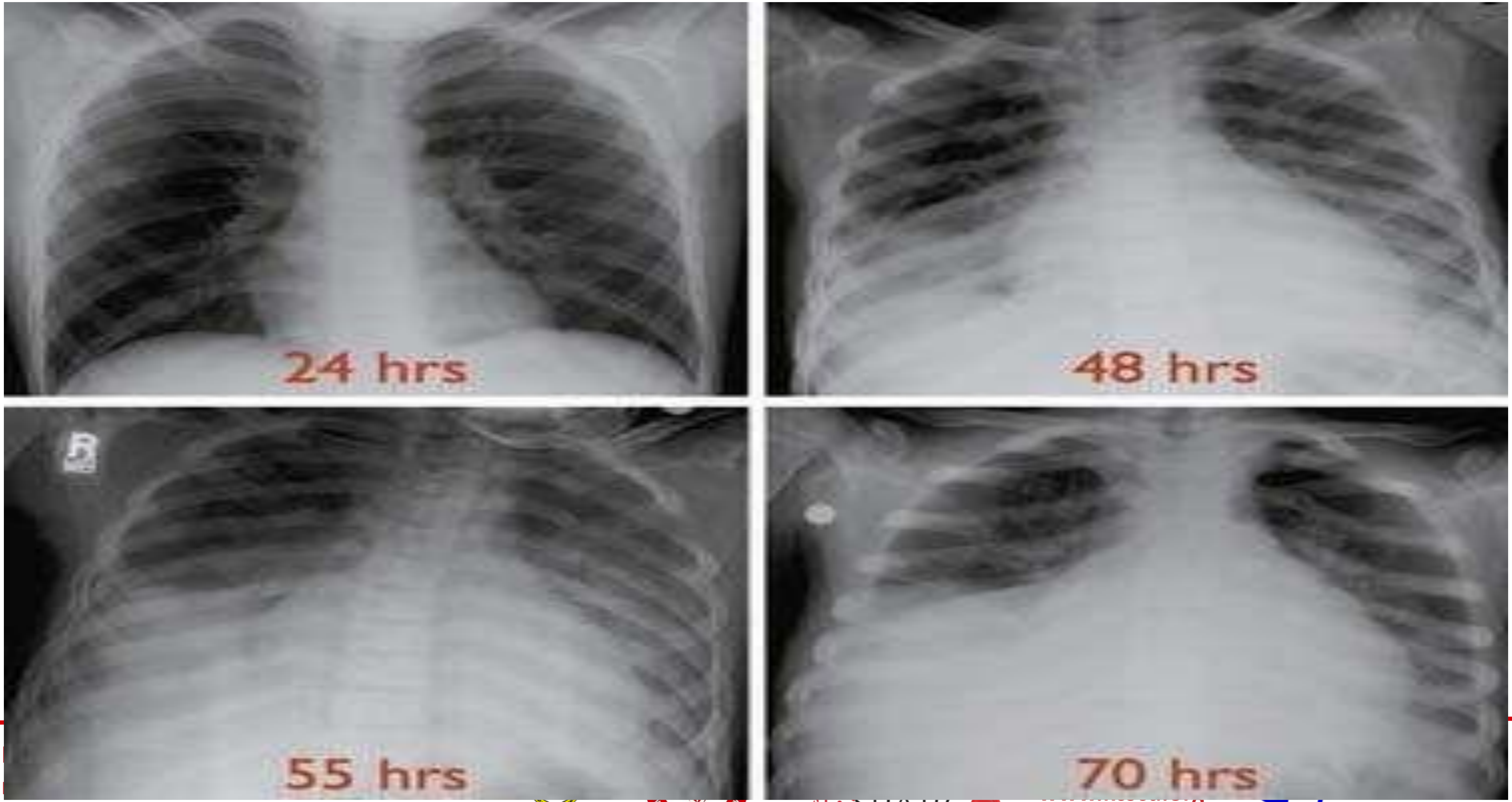

Sickle In Africa

Kwame Nkrumah University of Science and Technology (KNUST)

SPARCO Sickle Pan-African Research Consortium

ACS MANAGEMENT

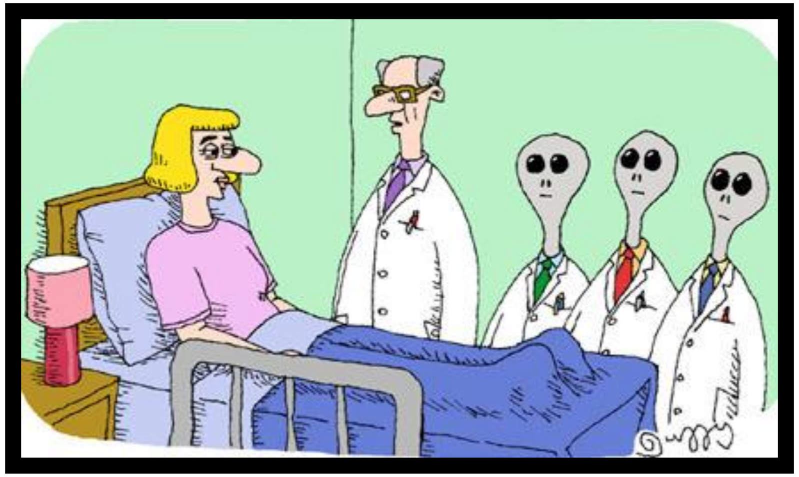

1. Oxygen
2. Fluid management
3. Pain relief
4. Blood transfusion
5. Antibiotics
6. Incentive spirometer or chest physiotherapy
7. Thromboprophylaxis

Oxygen therapy

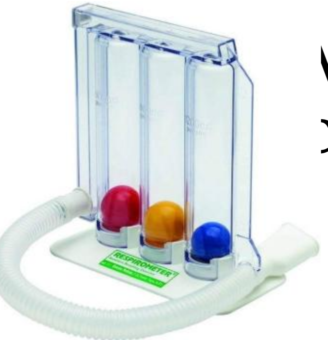

Maintain adequate oxygenation, improve oxygen-carrying capacity, and improve tissue oxygen delivery

- Administer supplemental oxygen to maintain oxygen saturation above 95%

- Prevent further alveolar collapse by using <u>INCENTIVE SPIROMETRY</u>
  - 10 maximum inspirations every 2 h during the day and while the patient is awake during the night.

- Balloon therapy

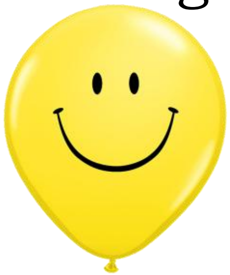

- Patient in clear respiratory distress whose oxygen falls to <85% when O2 is removed need escalation of therapy.

FLUID THERAPY

- Dehydration → ↑ plasma osmolarity + intracellular dehydration of RBCs ↓ sickled RBCs

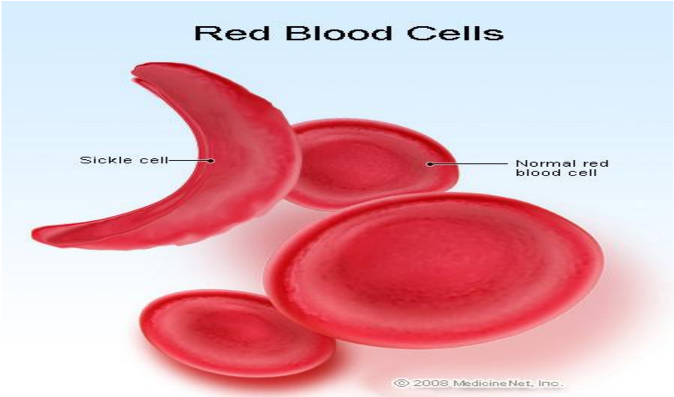

- Previous standard practise was : 1 – 1.5x maintenance fluids (Normal saline) ✗

FLUID THERAPY

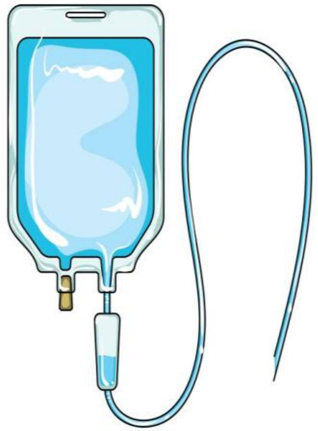

- Fluid requirements should be individualized and be guided by the patient's fluid balance and cardiopulmonary status.

- Patient's usually too ill to consume fluids orally
  - 5% dextrose or Dextrose Saline

- Monitor intake and output to prevent fluid overload
- For children weigh them daily.

PAIN CONTROL

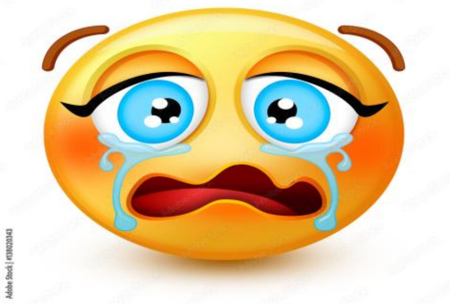

- Rapidly initiate analgesic therapy within 30minutes
- Analgesics should be based on pain assessment, patient's OPD use, patient's knowledge of effective agents and doses and past experience with side effects.
- WHO ladder for pain relief

BLOOD TRANSFUSION

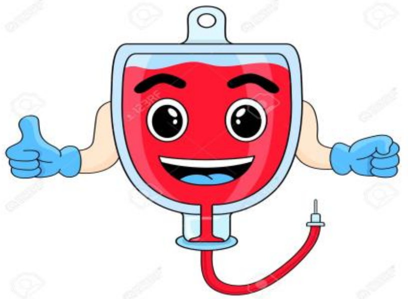

- Guidelines suggest giving simple transfusion to improve oxygen carrying capacity for patients with symptomatic ACS whose haemoglobin concentration falls 1g/dL or more below the baseline.

- The goal is a haemoglobin value of 10/dL. If the baseline Hb is 9g/dL or greater, simple blood transfusion may not be required.

Blood transfusion

- Exchange transfusions should be reserved for
  - Patients with a higher Hb
  - Rapid progression of ACS as manifested by oxygen saturation below 90% despite supplemental oxygen
    - Increasing respiratory distress
    - Progressive pulmonary infiltrates
    - And /or decline in haemoglobin concentration despite simple transfusion

  - It is important to decrease the hemoglobin S concentration rapidly.
  - Exchange transfusions avoid the problems related to increased blood volume and viscosity.

ANTIBIOTICS

- Broad spectrum antibiotics including cover for atypical organisms e.g. iv augmentin/ceftriaxone and azithromycin

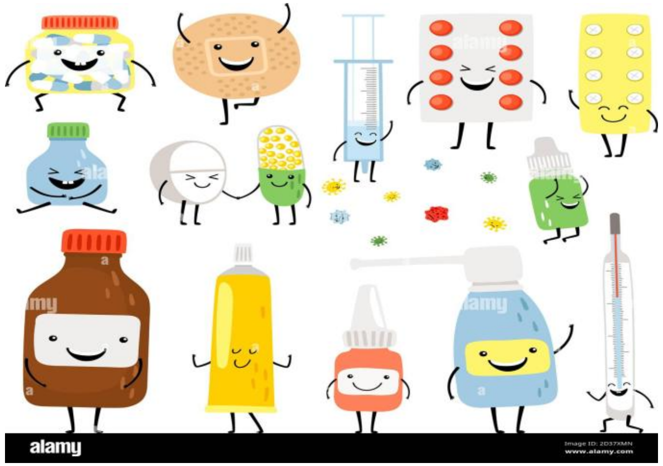

THROMBOPROPHYLAXIS

- Low molecular weight heparin(e.g. enoxaparin) prophylactic doses is recommended in the absence of any contraindications

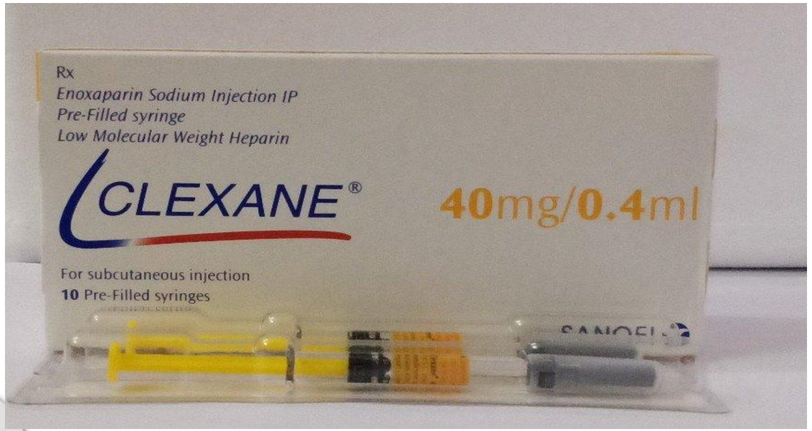

Differences between ACS in children and adults

Children	*Adults*
Seasonal predilection (winter > summer)	High rates of morbidity and mortality
Commonly present with fever, cough and wheeze	Present with chest pain, dyspnoea anc painful crisis
Radiographs show more frequent upper and middle lobe changes	Radiographs show lower and multilobe changes
Bacteraemia and viraemia more common	Organisms rarely isolated
Fewer transfusions necessary	High rate of transfusion

To do list on the ward

- Encourage Incentive Spirometry/balloon therapy

- Monitor vitals
  - Continuous SpO_2 monitoring
    - Oxygen saturation (SpO2) ≤94% (on air) on pulse oximetry or a fall in SpO2 of ≥3% from baseline steady state values should prompt further action
  - Temp, PR, BP, RR

- Daily pain score

- Daily chest examination

PREVENTION

- Hydroxyurea decreases the incidence of ACS in patients with recurrent severe pain and should be considered in any patient who has one or more episodes of acute chest syndrome

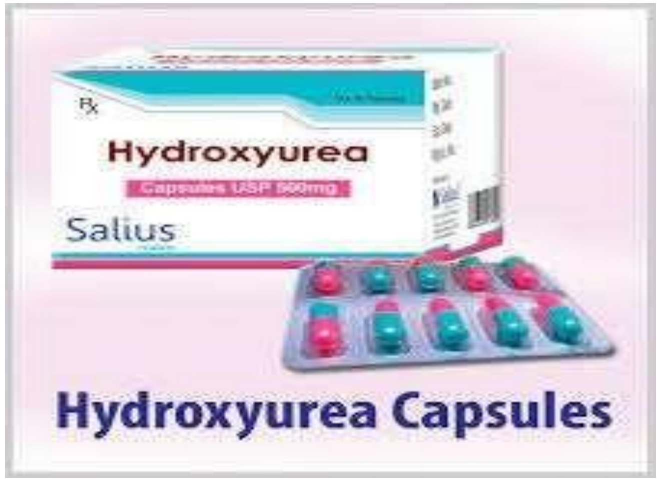

Pointers:

- Patients with SCD in general, can present with ACS, or it may develop sometime after onset of severe pain. Therefore, **vigilance** should be maintained throughout hospital admission.
- Clinicians should maintain a high index of suspicion of ACS in patients who have chest symptoms and signs, especially if hypoxic, even in the presence of a normal chest X-ray.

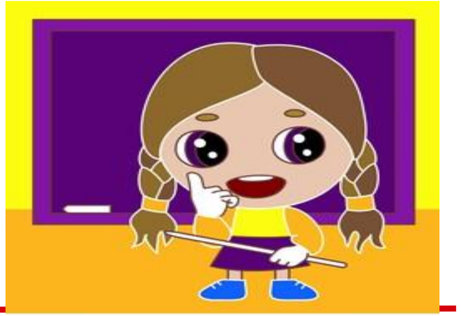

Pointers:

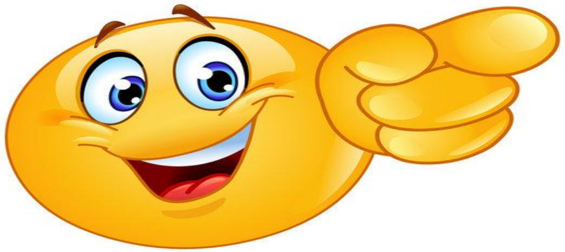

- Pulmonary embolism, fluid overload, opiate narcosis and hypoventilation may cause or trigger ACS and should be considered when a diagnosis of ACS is made as they may require additional treatment.

- Management of ACS is MULTI- DISCIPLINARY!!!!!!!!!!!!!!!

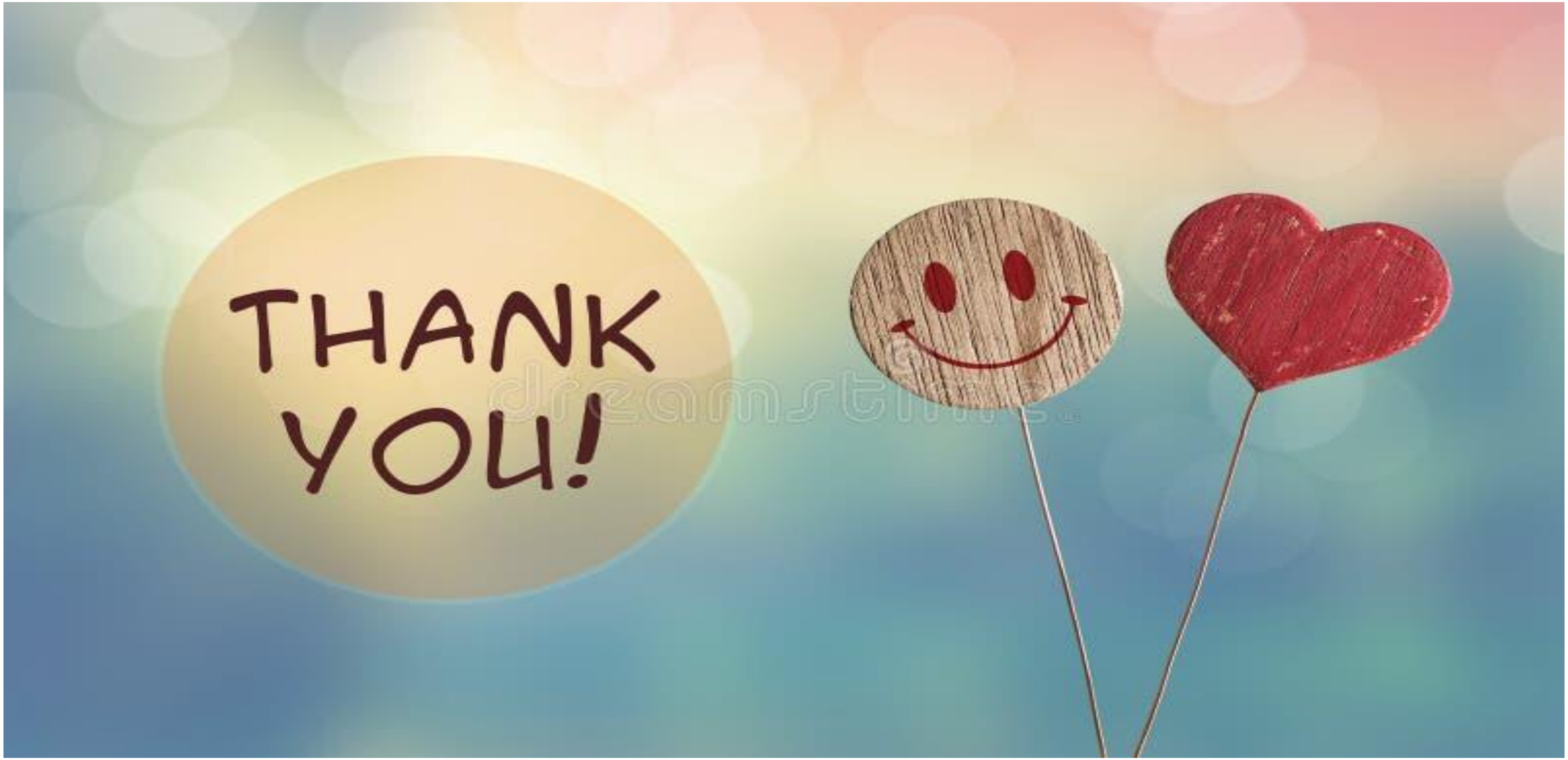
THANK YOU!

Sickle In Africa

ACUTE ANAEMIA IN SCD

OBJECTIVES

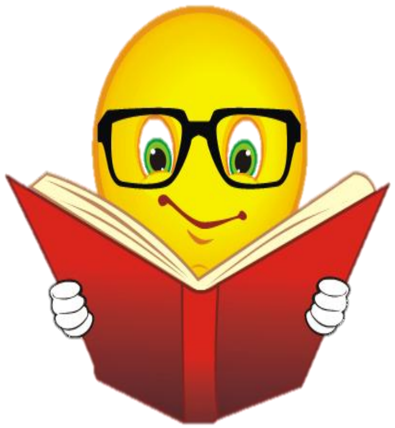

- Definition
- Causes
- Breakdown of each of the causes, investigations and management
- Conclusion

DEFINITION

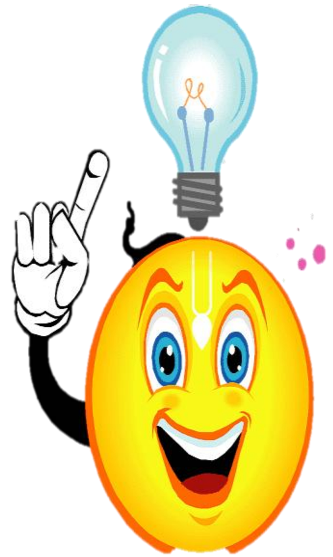

- Defined as the rapid significant fall in Hb of at least 2g/dl from baseline levels.

CAUSES

- Aplastic crisis
- Acute splenic sequestration crisis (ASS)
- Hyper-hemolytic crisis (HS)

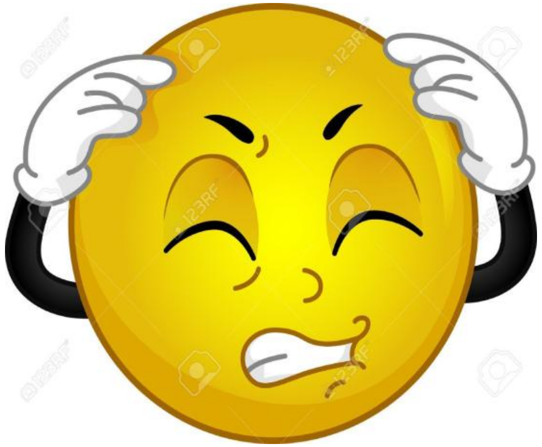

APLASTIC CRISIS

- Bone marrow of someone with SCD suddenly stops producing new red blood cells.
- This causes sudden and severe anemia.
- Caused by an infection with Parvovirus B19
- Aplastic crises typically affect more children than adults.

INVESTIGATING APLASTIC CRISIS

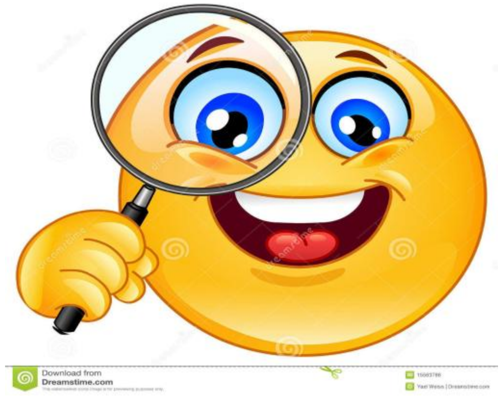

- Full blood count
- Retic count
- Serum IgM for Parvovirus B19 or presence of Parvovirus DNA to confirm the diagnosis of aplastic crisis

TREATMENT OF APLASTIC CRISIS

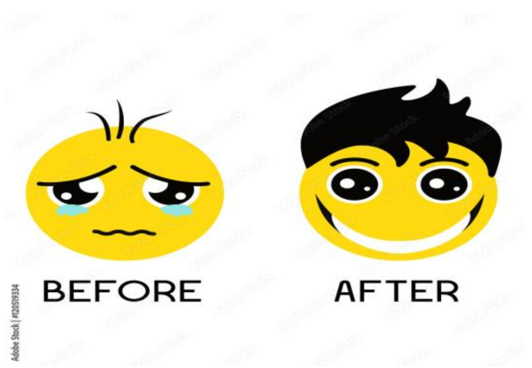

- Mainly supportive
  - IV fluids, blood transfusions

- Most people recover from aplastic crises within 10 to 14 days of starting treatment.

- Lifetime immunity, which reduces the risk of additional aplastic crises.

ACUTE SPLENIC SEQUESTRATION CRISIS

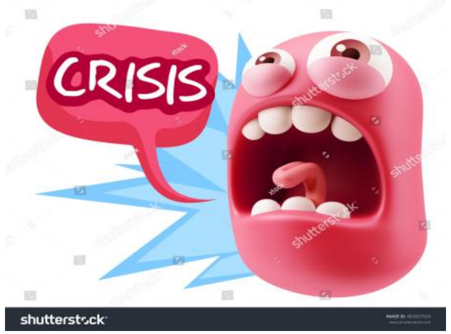

- Life-threatening complication.

- ASSC is defined as acute splenic enlargement with a fall in the haemoglobin (Hb) level of at least 2g/dl and a normal basal reticulocyte count.

- To date, no factors predicting ASSC have been identified.

ASSC

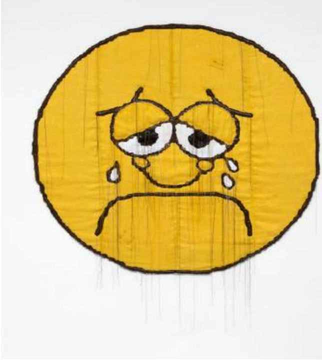

- Acute intrasplenic sickling traps blood in the spleen, leading to a decrease in the circulating blood volume.

- Hypovolaemic shock may ensue unless a sufficient circulating volume is restored promptly, usually by blood transfusion.

INVESTIGATING ASSC

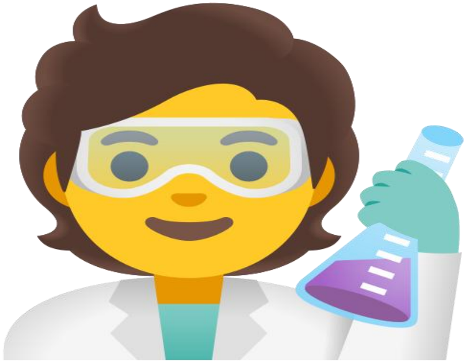

- Mainly a clinical diagnosis

LABs

- FBC
- Reticulocytes count
- Abdominal Ultrasound

MANAGEMENT-ASSC

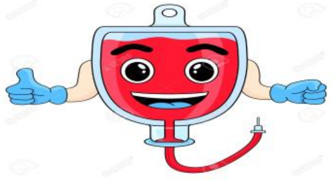

- Transfuse **carefully** in small quantities starting with the number of ml /kg of red cells equal to the HB number of grams/dL
  - E.g. for patients with HB 3g/dL, give 3ml/kg as initial transfusion.
- Wait 3-4 hours for equilibration and give subsequent small-volume transfusions as needed.

MANAGEMENT OF ASSC

- NB: Do not aim for baseline or normal Hb level in initial transfusions or Hb>10g/dL,
- The sequestered red cells can reenter circulation hours or days after RBC transfusion and may increase the risk of acute cardiac failure, hypertension and stroke.
- Monitor splenic size daily

MANAGEMENT OF ASSC

- Splenectomy (recurrent sequestration or a single life-threatening sequestration episode.
- Vaccinations indicated after splenectomy
  - Pneumococcal, meningococcal and Haemophilus influenzae(Hib)
- Penicillin prophylaxis post-splenectomy

HYPERHEMOLYTIC CRISIS

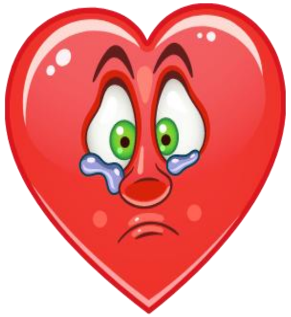

- Sudden exacerbation of hemolysis with worsening anaemia despite ongoing reticulocyte production

- May be associated with acute vaso-occlusive events including ACS and VOPE

- Infections and/or drug exposure may be the trigger in some cases

CLINICAL PRESENTATION OF HHC

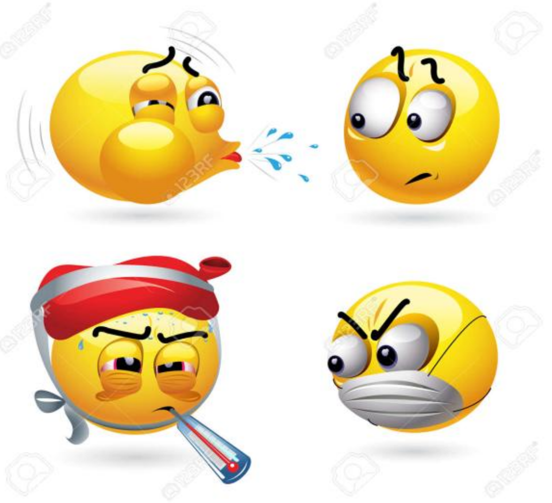

- Jaundice
- Symptoms/signs of anaemia
- Haemoglobinuria
- AKI
- +/-Fever

INVESTIGATIONS HHC

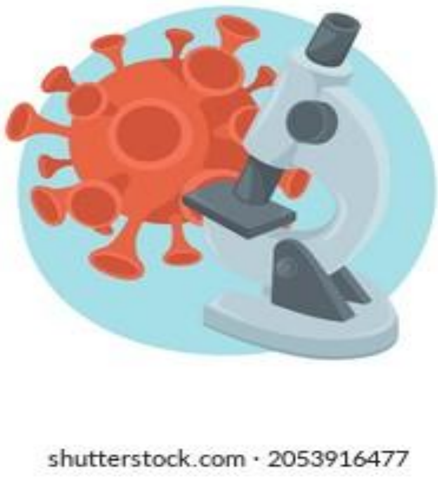

- FBC
- Retic count.
- LDH
- Indirect bilirubin
- Blood film

TREATMENT

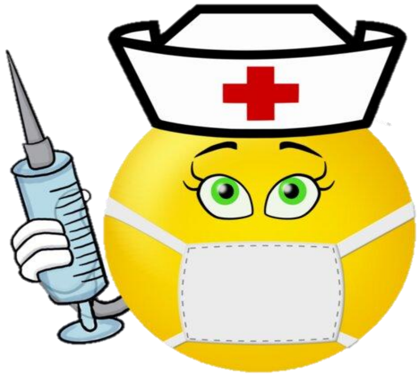

- The management is mainly supportive.
- IV fluids
- Transfusion
- Treat underlying cause e.g. infection
- Monitor FBC, serial urine collection, RFT

CONCLUSION

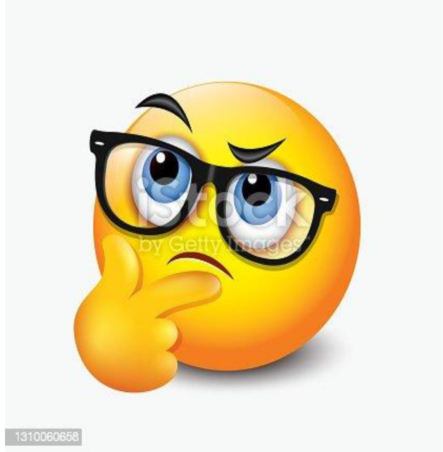

- Acute anaemia in SCD is potentially life threatening

- There should be a high index of suspicion

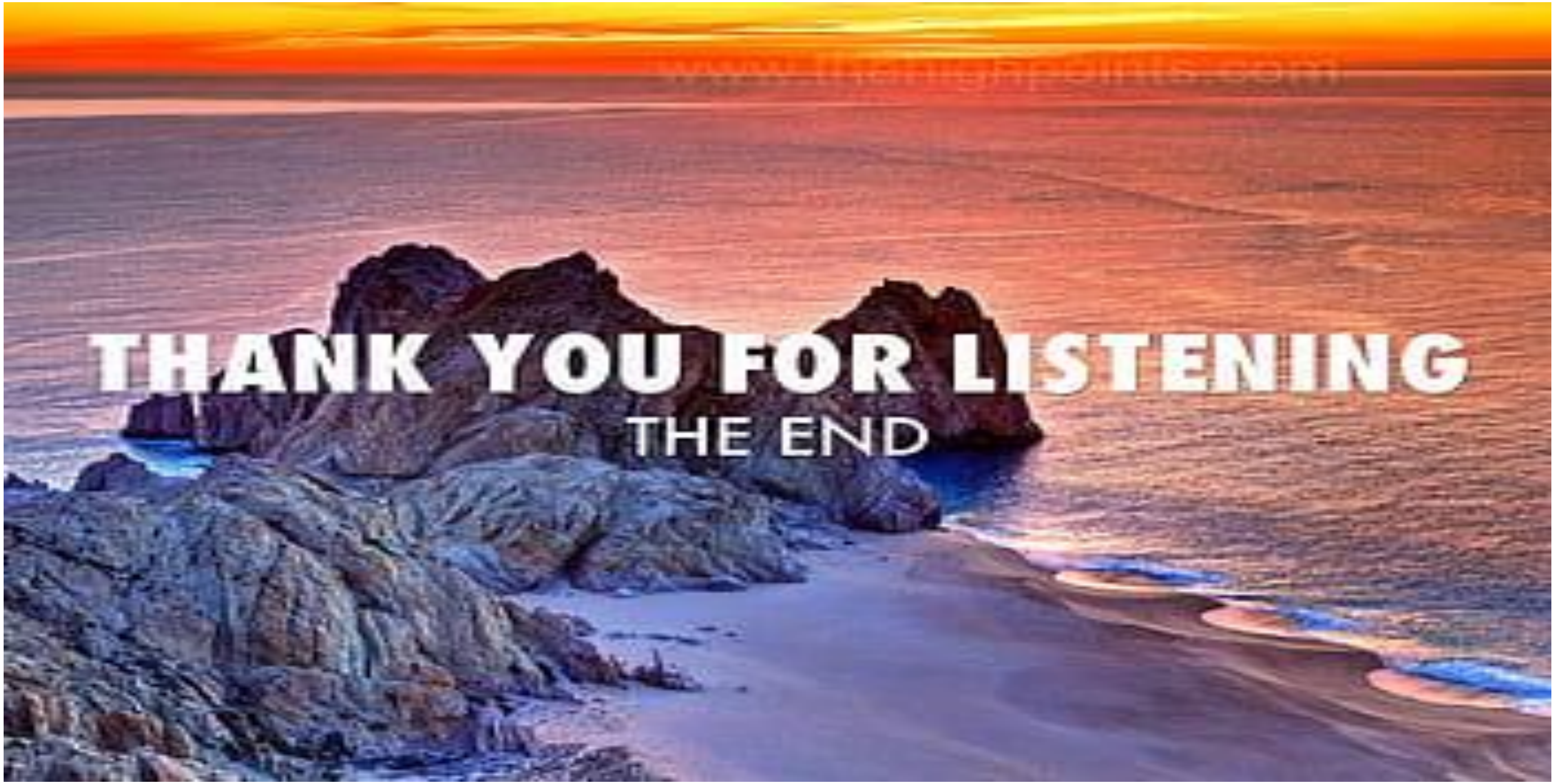
THANK YOU FOR LISTENING
THE END

STROKE IN SCD

Pretest (T/F)

- 4 yrs old male with SCD(SS) presented to the PEU with chest pain and fever. His BP was 130/65mmHg. Hb was 6.5g/dl
- He was admitted and managed for Acute chest syndrome and was hemotransfused. Post transfusion Hb was 13g/dl
- He was on Pen V and folic acid
- He started having seizures and imaging of the head showed he had developed an cerebral infarct.

Q1

What are the risk factors for stroke in this patient.

1. Hypertension
2. Baseline Hb of 6.2g/dl
3. Post transfusion hb of 13g/dl
4. Sickle cell disease
5. Gender

Outcomes of Presentation

- Know the types of stroke in SCD
- Explain the Mechanism of injury in CNS complications
- Management of stroke
- Levels of stroke prevention
- How to manage stroke in patients with SCD

OUTLINE

- Introduction
- Types
- Pathophysiology of CNS injury
- Approach to management
- Levels of stroke Prevention in SCD

Introduction

- Stroke is the most Disabling of all the complications of SCD
- A child with SCD is 300 times more likely to have a stroke
- 11% of patients with SCA will develop a stroke by their 18th birthday if they receive no intervention
- The peak age for stroke in SCD is 2-5 yrs.
- Over the last 40 years, significant clinical effort has been focused on preventing initial and subsequent CNS injuries.

Types of Strokes in SCD

- Ischaemic/Infarctive stroke (87%)
  - Transient ischaemic attack*
  - Silent cerebral infarct (39% by their 18th birthday)
- Hemorrhagic stroke (13%)

Risk factors for Ischaemic stroke

- Low baseline hemoglobin
- High Hemoglobin S (>30%)
- Cerebral Vasculopathy
- Low Hemoglobin oxygen saturation or hIgh O2 demand
- Frequent painful events
- Prior TIA
- Co-morbidities

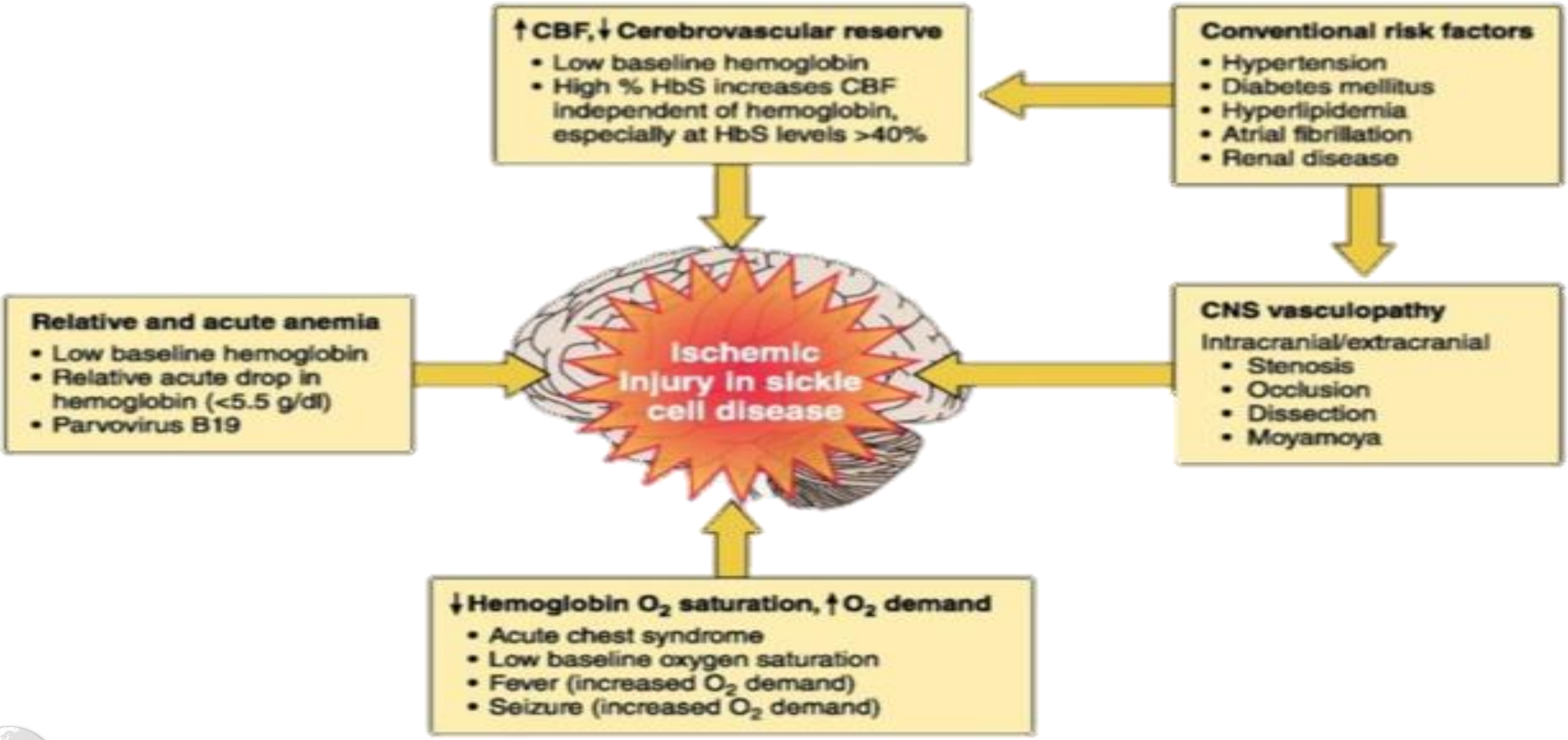
↑CBF, ↓Cerebrovascular reserve
• Low baseline hemoglobin
• High % HbS increases CBF independent of hemoglobin, especially at HbS levels >40%
Conventional risk factors
• Hypertension
• Diabetes mellitus
• Hyperlipidemia
• Atrial fibrillation
• Renal disease
Relative and acute anemia
• Low baseline hemoglobin
• Relative acute drop in hemoglobin (<5.5 g/dl)
• Parvovirus B19
Ischemic injury in sickle cell disease
CNS vasculopathy
Intracranial/extracranial
• Stenosis
• Occlusion
• Dissection
• Moyamoya
↓Hemoglobin O_2 saturation, ↑O_2 demand
• Acute chest syndrome
• Low baseline oxygen saturation
• Fever (increased O_2 demand)
• Seizure (increased O_2 demand)

Presentation

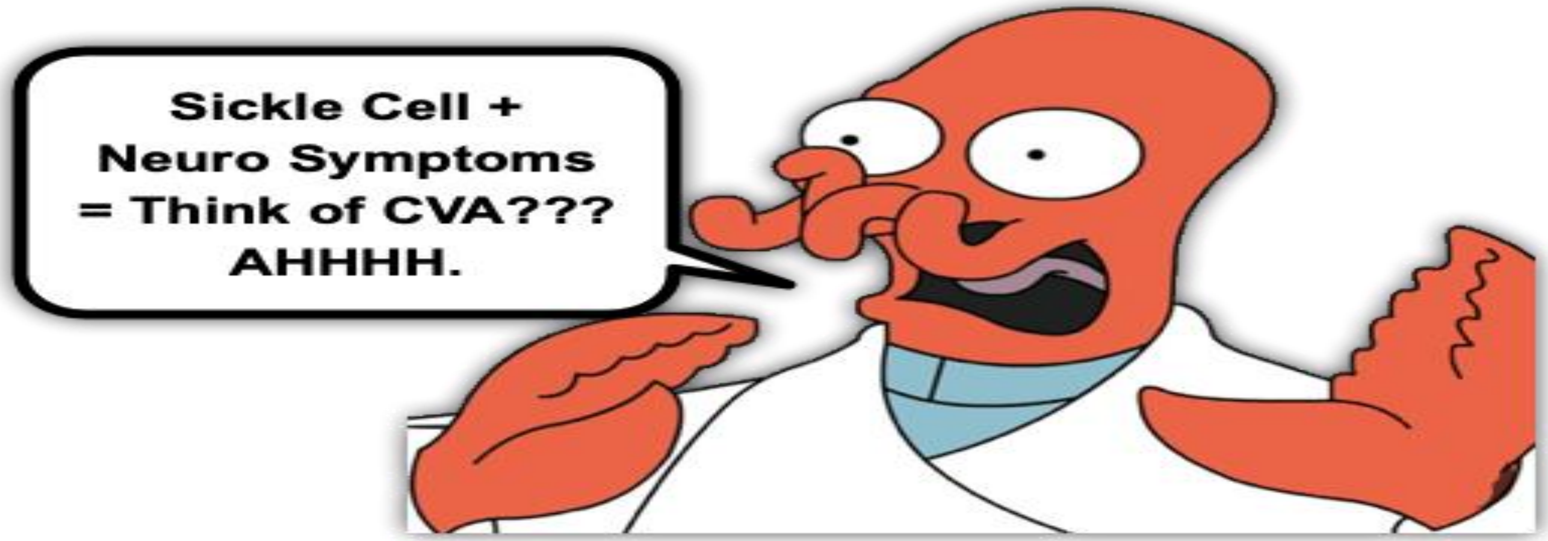

Presentation of Stroke

- If it looks, sounds or feels like a stroke……. It is a stroke
  - Face drooping
  - Arm or leg weakness
  - Speech difficulties
  - Seizures
  - Coma
  - Headache
  - Sight problems
  - Confusion
  - Time to come to the emergency unit

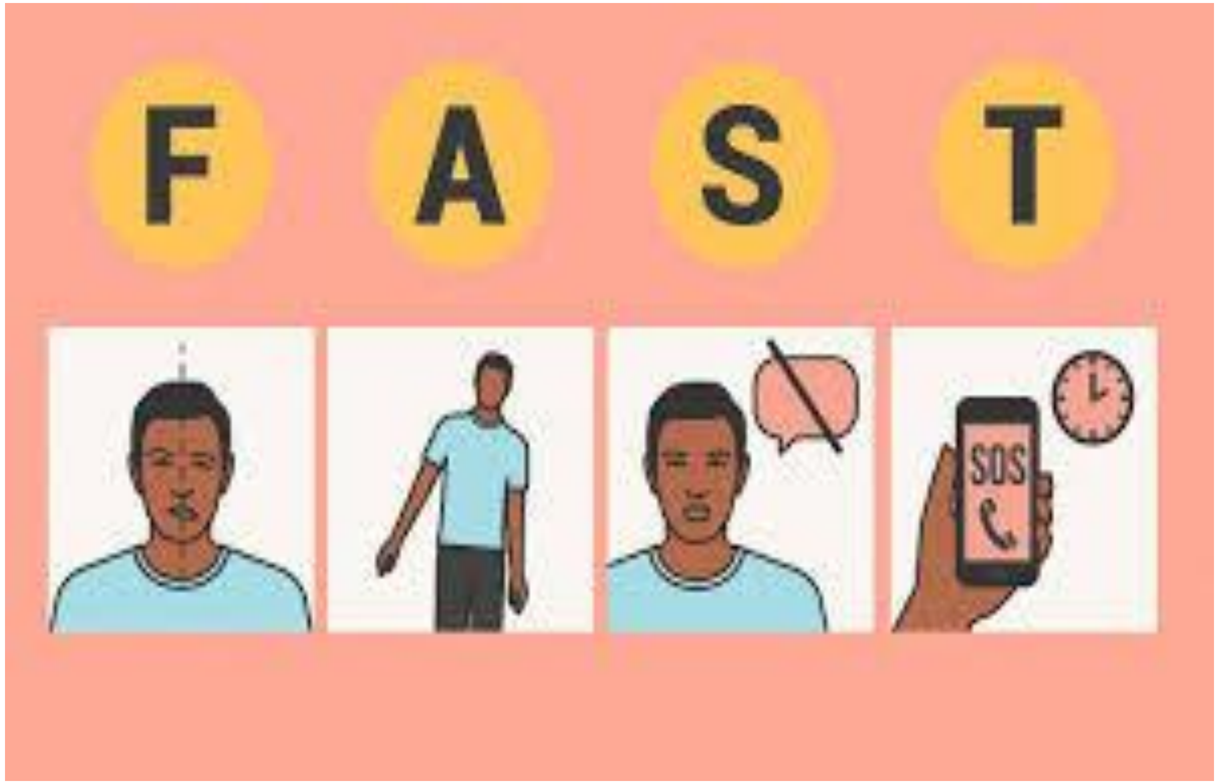

GOALS OF MANAGEMENT

- Stabilize patient
- Evaluation/assessment
- Identification and Correction of risk factors
- Prevent further brain injury

Steps in Acute Stroke Management

❖Acute Management

- Airway , Breathing and circulation

❖Assessment(History and physical examination)

- If it looks, sounds or feels like a stroke……. It is a stroke

❖Investigations

❖Transfusion :

- Simple transfusion can be used (goal Hgb of 10)
- Exchange transfusion is treatment of choice < reduce HbS <30%

Investigations

❖Diagnostic:

- **Head CT**
- **CT angiography of the head and perfusion**
- **MRI/MRA is preferred imaging of choice** for ischemic infarcts but needs to be performed quickly

❖Supportive

- CBC with differentials & reticulocyte count,
- PT/INR, GXM(pRBC)
- Blood culture if infection suspected,
- Hemoglobin S quantification

Supportive care

- Avoidance of hypotension(attention to blood pressure control)
- Avoid hyperthermia, hyperglycaemia and hypocarbia.
- Hydration with N/S at maintenance rate. (avoid hypotonic saline)
- Treatment of underlying infection and antipyretics
- Management of seizure and raised ICP

Hemorrhagic Stroke

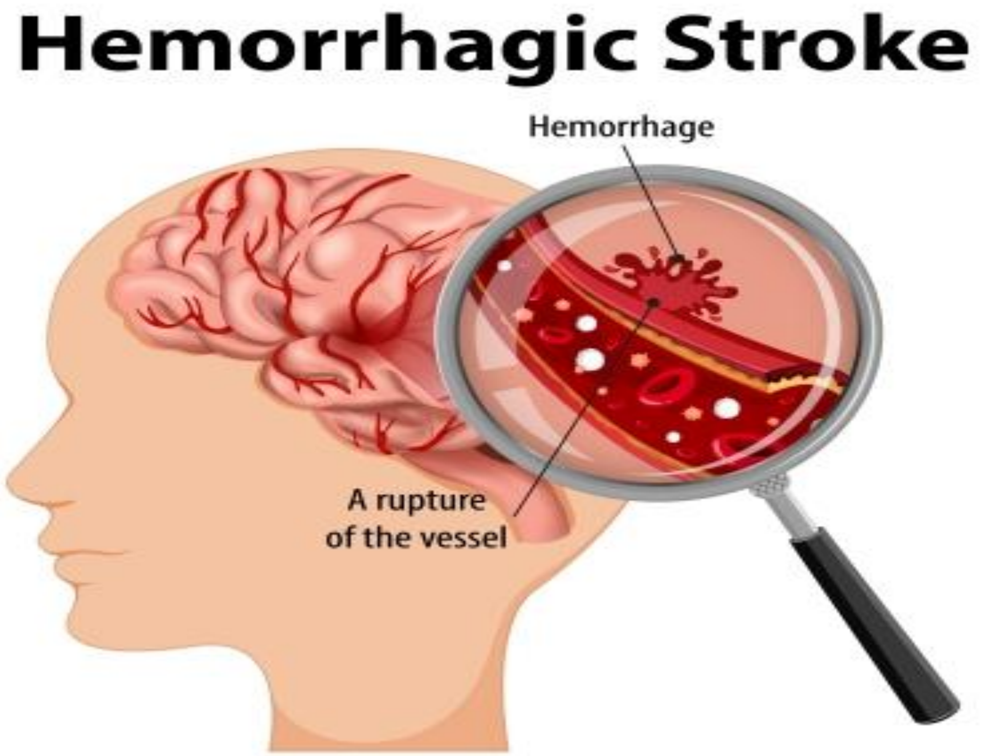

- Urgent CT scan
- Manage Raised ICP
- Neurosurgery consult

Transcranial Doppler

- TCD is a screening tool used to identify children with SCD who are at high risk for stroke by documenting abnormally high blood flow velocity in the large arteries of the circle of Willis—the middle cerebral or internal carotid arteries.
- Higher velocities indicate either elevated cerebral blood flow, in which case the TCD shows widespread increases in velocity, or an area of reduced arterial diameter, or stenosis, in which case there is a focal increase in velocity

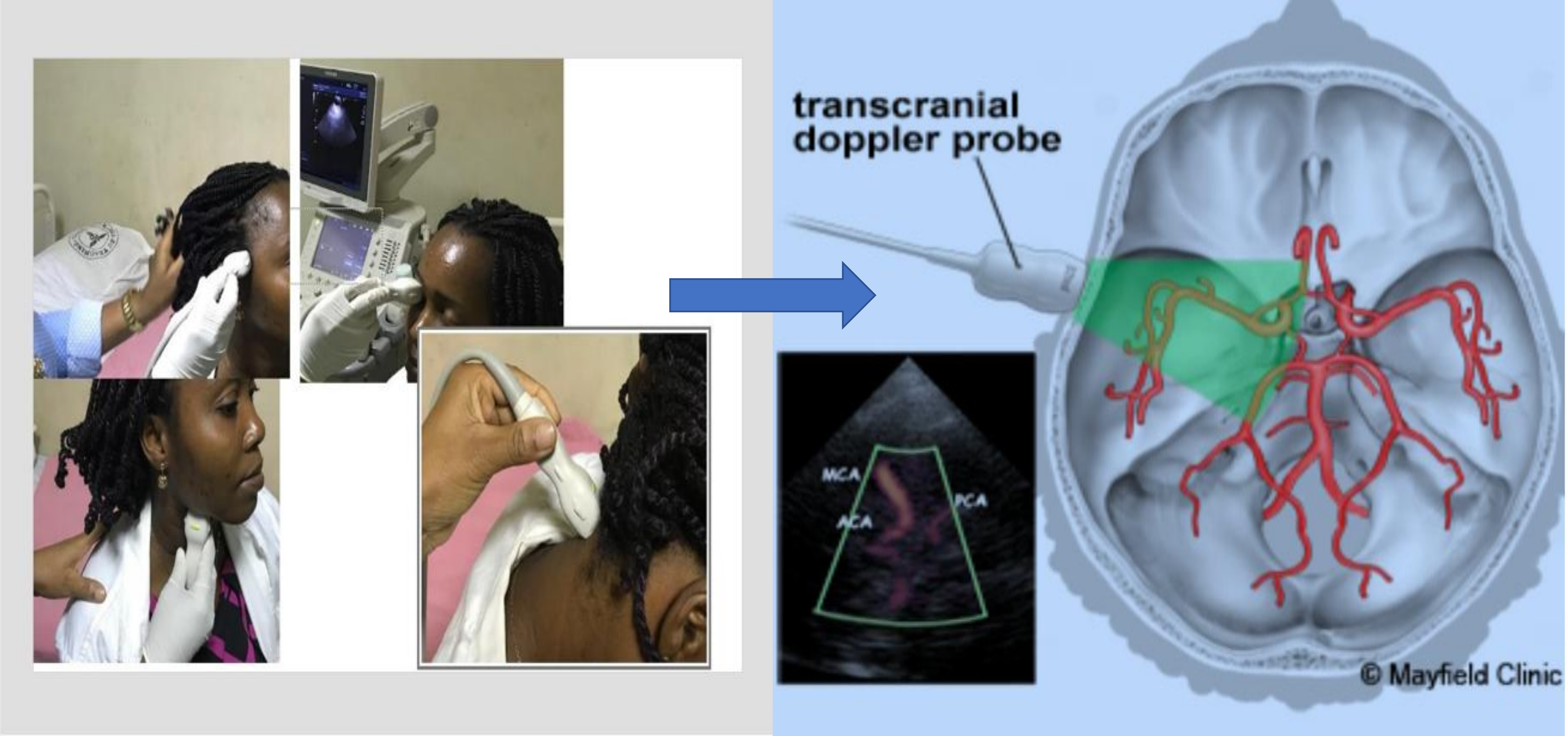
transcranial
doppler probe
MCA
ACA
PCA
© Mayfield Clinic

TCD Readings and Interpretation

TCD READING	VALUES (cm/s)	Action	Frequency of TCD
NORMAL	<170		Annually
Conditional	170-200	Continue monitoring	Repeat 4-6 monthly
Abnormal	> 200	CTT or HU	Repeat 4 weekly

Primary prevention of stroke

- Annual TCD for children with HbSS and SBo thalassemia (2-16yrs)

- Those with abnormal TCD (>200cm/sec) should go for CTT

- Children (ages2-16years) with HbSS,HbS beta^{0} thalassemia, or compound heterozygous SCD who have abnormal TCD screening and live in LMICs hydroxyurea therapy is recommended

Prevention of stroke recurrence

- Chronic transfusion therapy with between 10-11g/dl and HbS <30%

- For adults and children with SCD moyamoya syndrome, and a history of stroke or TIA, evaluation for revascularization surgery in addition to regular blood transfusion is recommended.

CONCLUSION

- Stroke which is the most debilitating complication of SCD is preventable.
- TCD surveillance must be the norm and appropriate treatment will significantly decrease occurrence of primary strokes in children with SCD.
- Children can hide neurologic deficits if you don't perform a careful, age centered and thorough neurologic exam.
- Remember to take a picture ie neuroimaging when stroke is suspected.

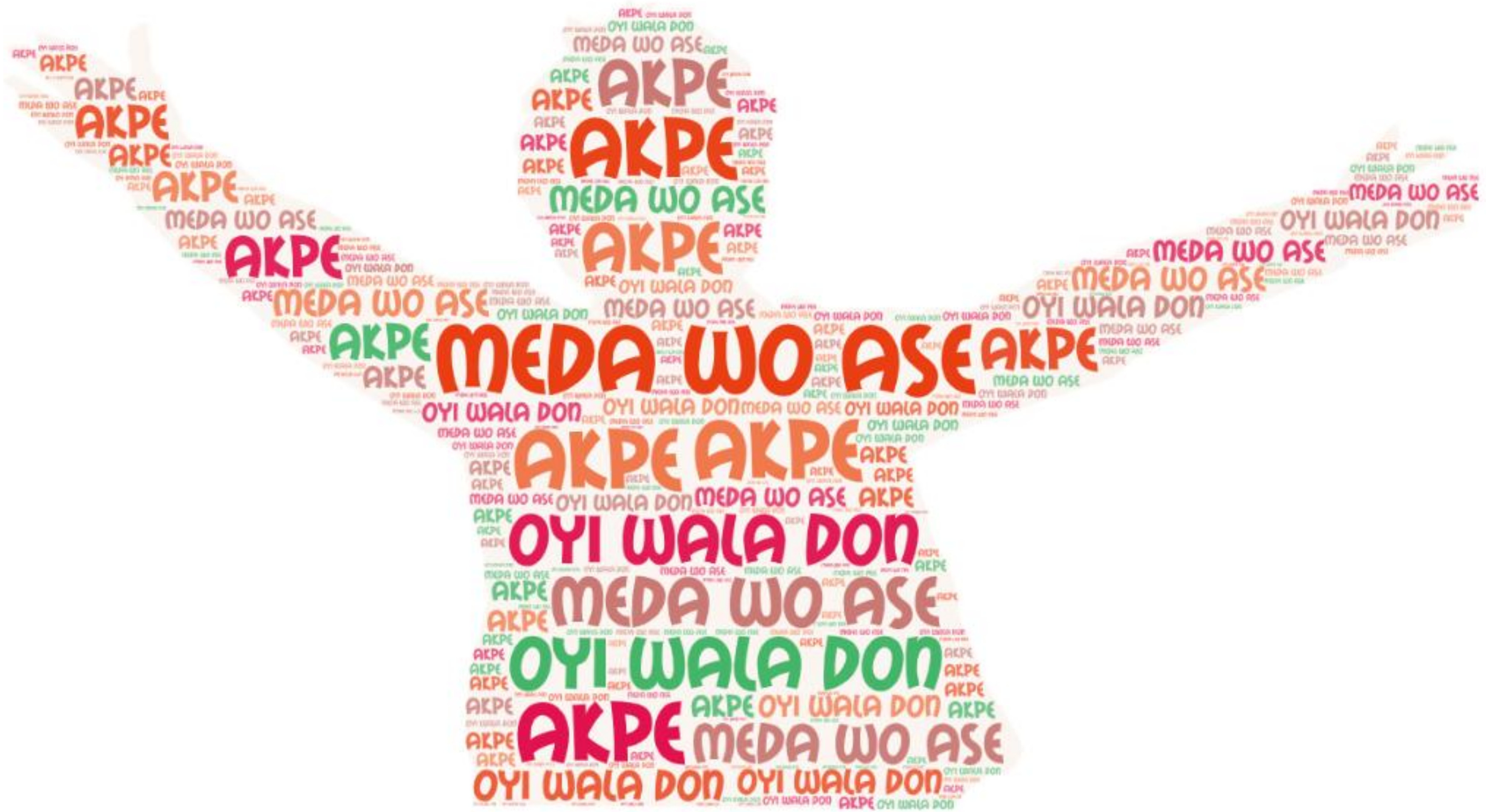
MEDA WO ASE
OYI WALA DON
AKPE

SCD CHRONIC COMPLICATIONS

Dr Eunice A. Ahmed
Haematologist-KATH

OUTLINE

- INTRODUCTION
- CHRONIC COMPLICATIONS
- NEPHROPATHY
- RETINOPATHY
- LEG ULCERS

CHRONIC SYMPTOMS & COMPLICATIONS

- Repeated vasoocclusion
- Chronic inflammation
- Blood vessel damage

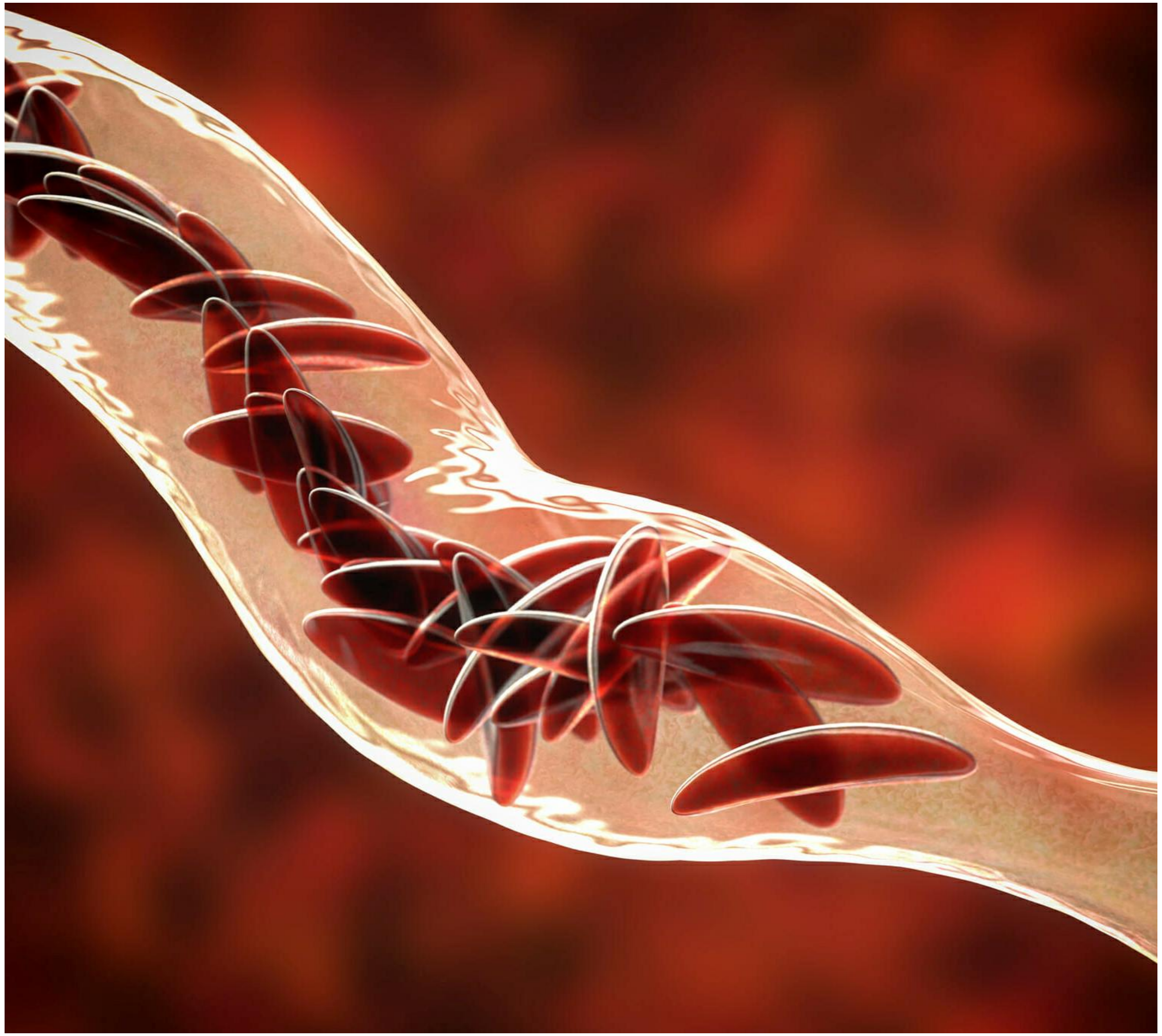

MULTISYSTEMIC DISEASE

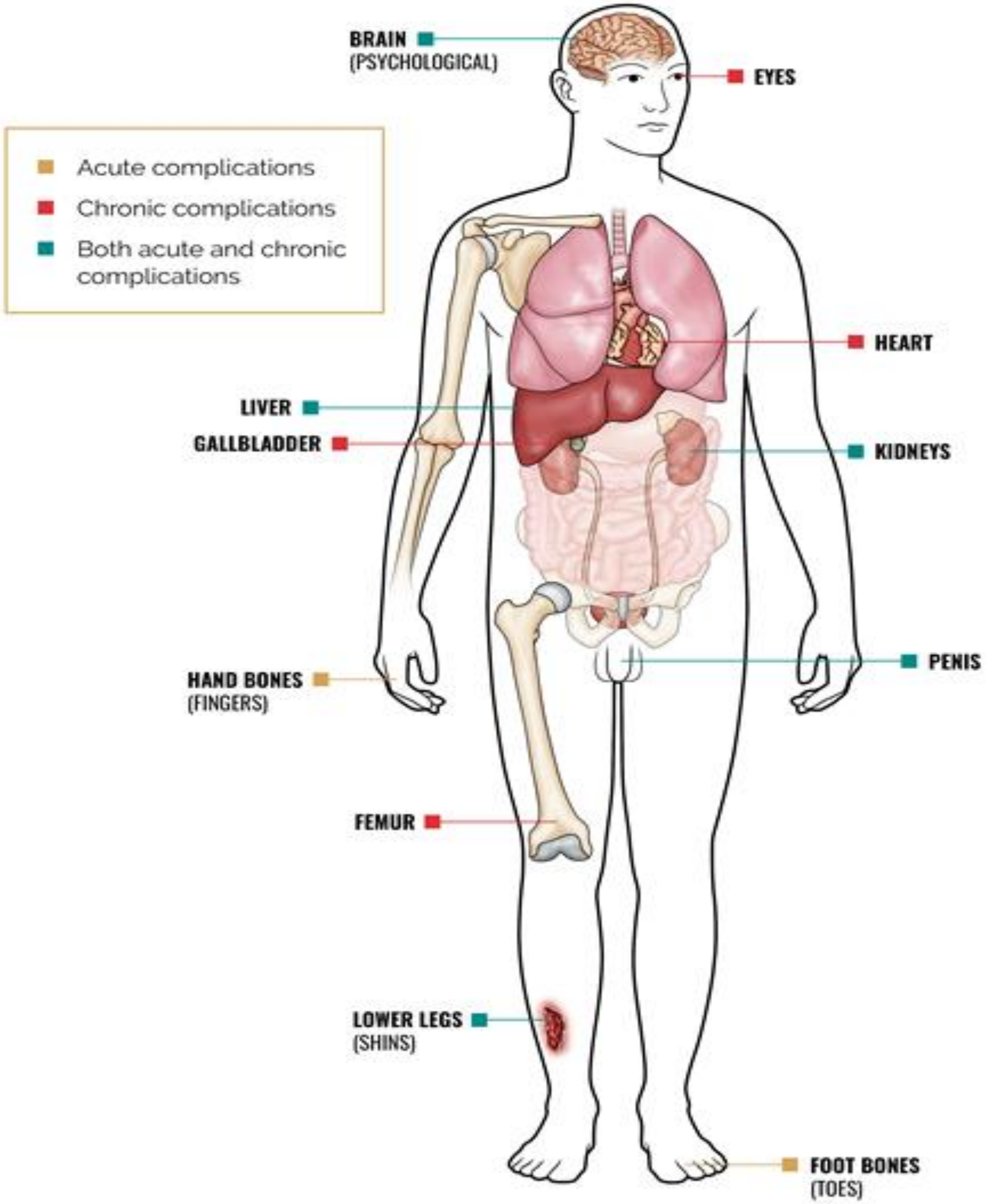

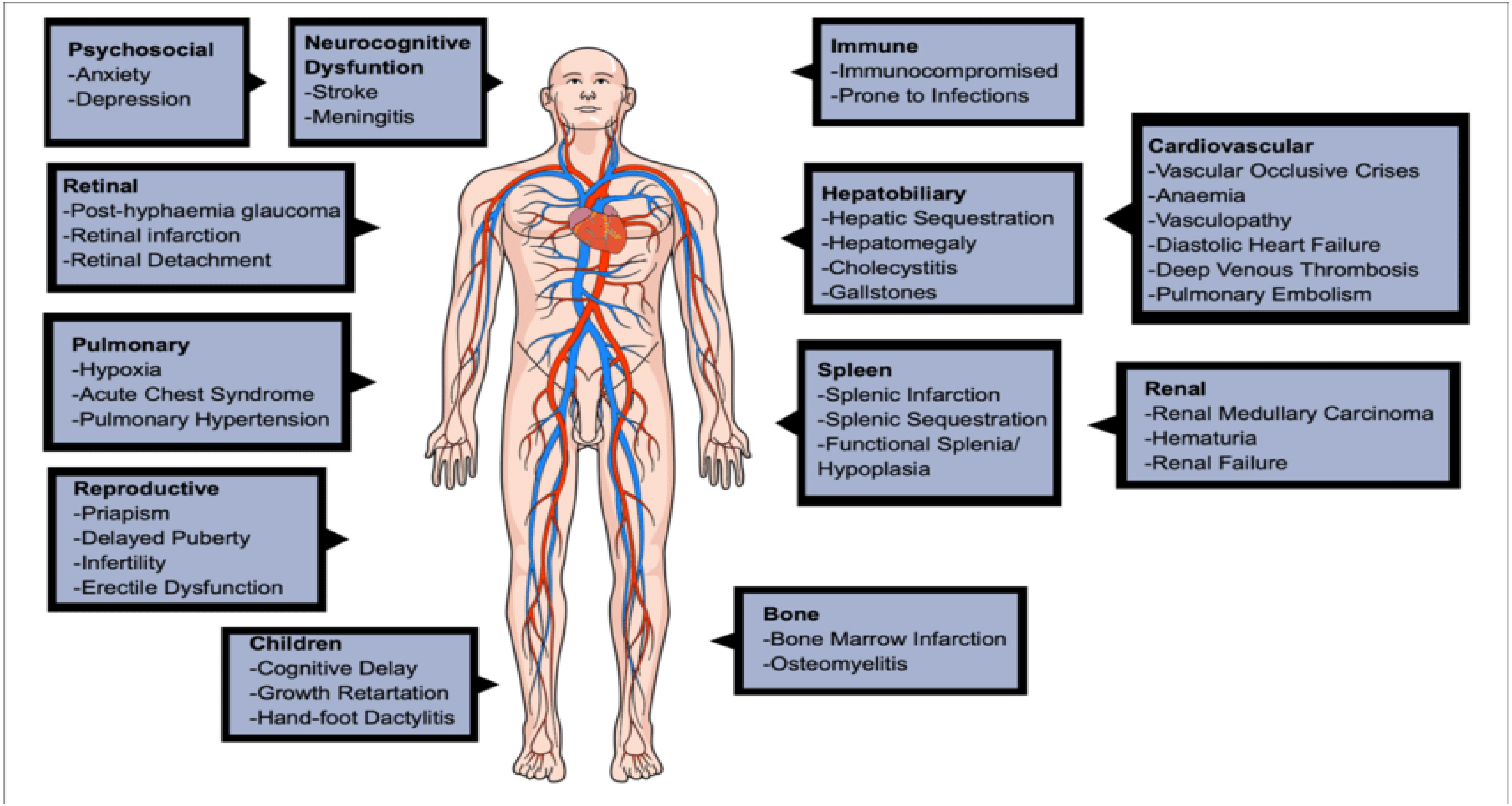
Psychosocial
-Anxiety
-Depression
Neurocognitive Dysfuntion
-Stroke
-Meningitis
Retinal
-Post-hyphaemia glaucoma
-Retinal infarction
-Retinal Detachment
Pulmonary
-Hypoxia
-Acute Chest Syndrome
-Pulmonary Hypertension
Reproductive
-Priapism
-Delayed Puberty
-Infertility
-Erectile Dysfunction
Children
-Cognitive Delay
-Growth Retartation
-Hand-foot Dactylitis
Immune
-Immunocompromised
-Prone to Infections
Hepatobiliary
-Hepatic Sequestration
-Hepatomegaly
-Cholecystitis
-Gallstones
Cardiovascular
-Vascular Occlusive Crises
-Anaemia
-Vasculopathy
-Diastolic Heart Failure
-Deep Venous Thrombosis
-Pulmonary Embolism
Spleen
-Splenic Infarction
-Splenic Sequestration
-Functional Splenia/ Hypoplasia
Renal
-Renal Medullary Carcinoma
-Hematuria
-Renal Failure
Bone
-Bone Marrow Infarction
-Osteomyelitis

NEPHROPATHY

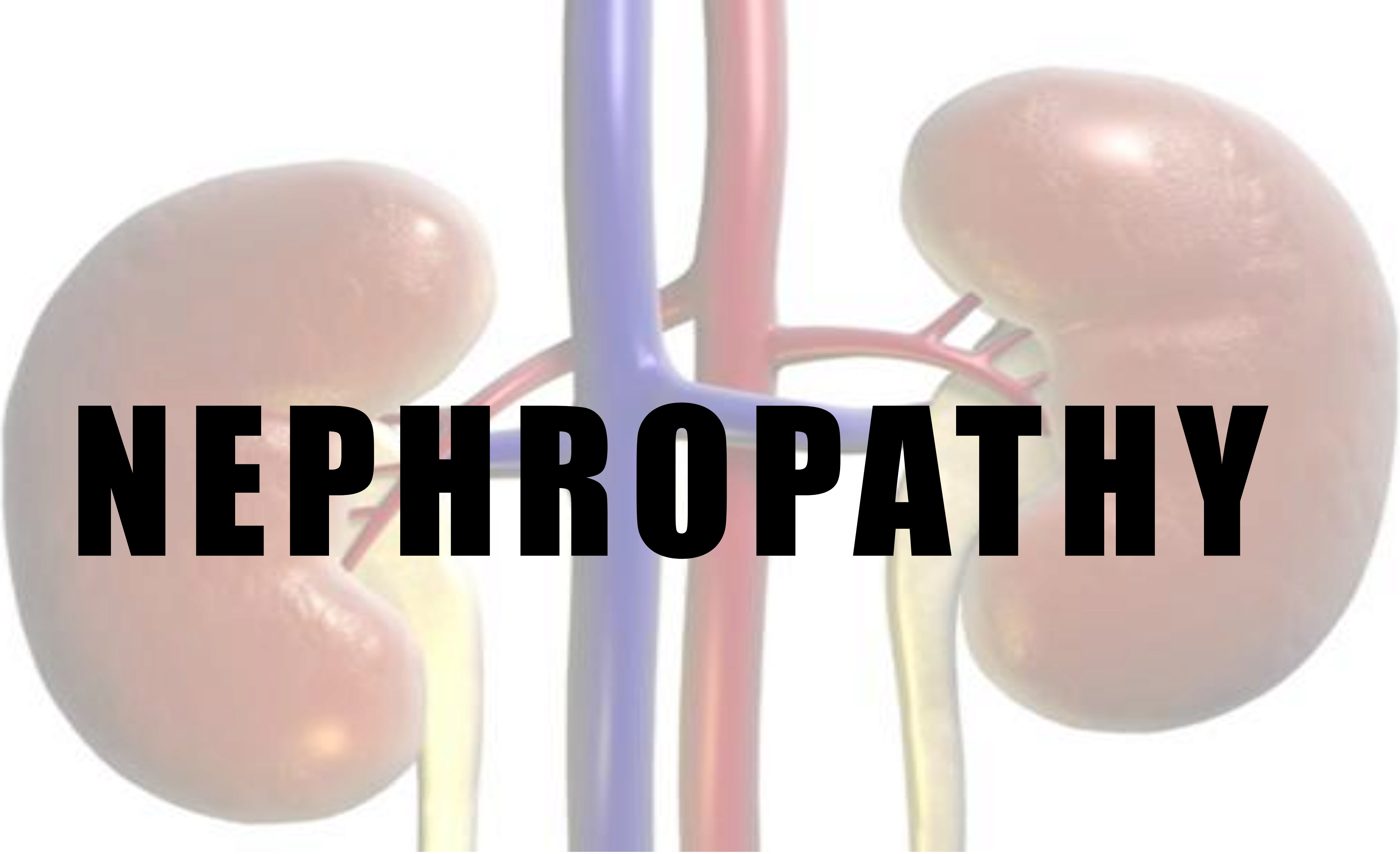

SICKLE CELL NEPHROPATHY

- SCD has several effects on the kidney, collectively referred to as "sickle cell nephropathy" (SCN)
- Kidney damage is multifactorial
- The renal medulla is the major site of kidney injury. (Vasa recta capillaries)
- Sickling affects the renal vasculature leading to renal medullary ischemia and infarction, with gradual loss of glomerular and tubular function

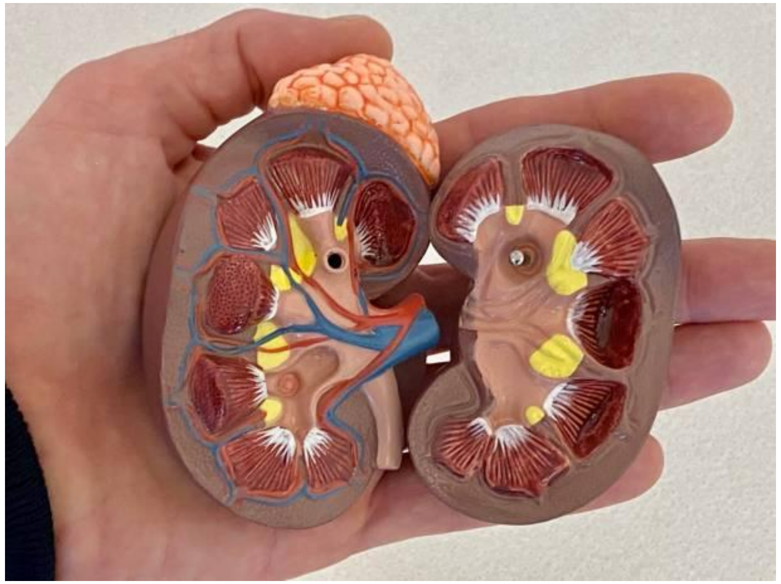

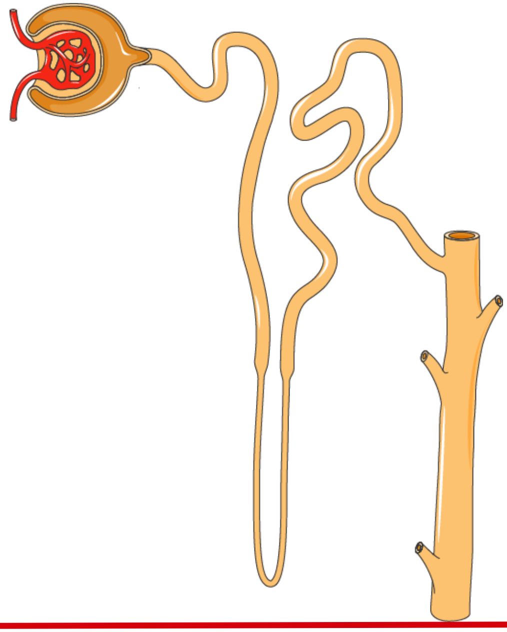

SCN

- Severity and age of onset of SCN is variable
- SCN is more severe with(Hb SS and s-b^0 thal)
- The diagnosis of CKD in patients with SCD generally occurs between 30 and 40 years of age
- 5%-18% of patients with SCD develop ESRD.
- Overall mortality in patients with SCD, 16%-18% is ascribed to kidney disease.

MANIFESTATIONS OF SCN

- Glomerular hyperfiltration
- Hyposthenuria
- Enuresis
- Proteinuria, microalbuminuria
- Haematuria (papillary necrosis)
- AKI, rapid increase in serum creatinine; associated with hospitalization)
- FSGS that can lead to ESKD
- Hypertension
- Medication toxicities (NSAIDS)

APPROACH TO MANAGEMENT

- Routinely ask parents about nocturnal enuresis in the child with SCD aged 6 years and older
- Document the presence of enuresis and give information to parents and other caregivers.
- Assure parents that nocturnal enuresis is a known and common complication of SCD.
- Refer the child to an enuresis management program, if one exists, for training in the use of enuresis alarms, and provide family counselling to avoid punitive measures that further lower the child's self-esteem.
- Try oral nasal desmopressin if other methods fail.
  Do not withhold fluid intake in the management of enuresis in children with SCD

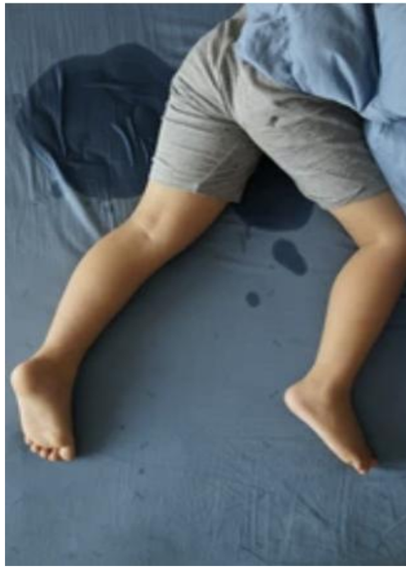

APPROACH TOMANAGEMENT

- Screen all individuals with SCD, beginning by age 10, for proteinuria.
- If the result is negative, repeat screening annually.
- If the result is positive, perform first-morning void urine albumin-creatinine ratio and if abnormal, consult with or refer to a renal specialist
- Patients with hematuria should be referred to a secondary or tertiary health facility for exclusion of other causes
- Encourage high fluid intake.

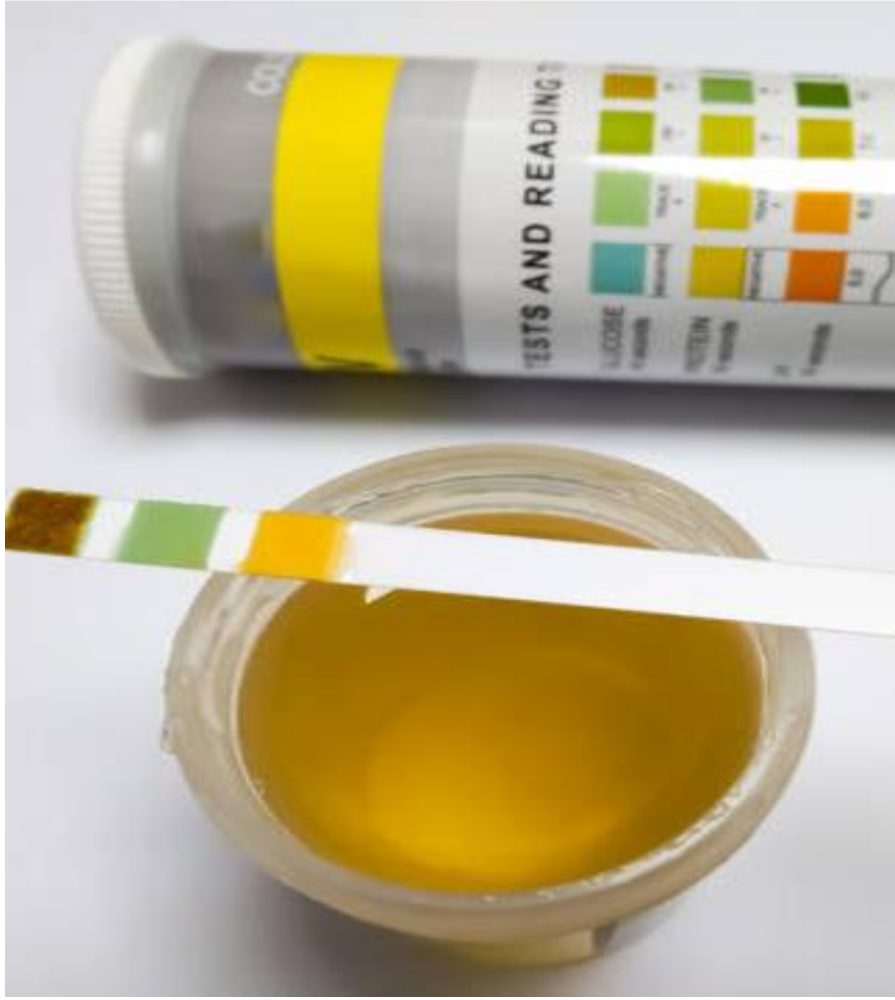

APPROACH TO MANAGEMENT

If microalbuminuria or macro-albuminuria is identified, order a 24-hour urine test for protein.

•

- Refer people with proteinuria (>300 mg/24 hours) to a nephrologist for further evaluation.
- For adults with proteinuria without other apparent cause, initiate ACE inhibitor therapy.
- For children with microalbuminuria or proteinuria, consult a nephrologist
- Consider patients with SCD with modest elevations of serum creatinine (>0.7 mg/dL in children, >1.0 mg/dL in adults) to have renal impairment and refer to a nephrologist for further evaluation.
- Give ACE inhibitor therapy for renal complications when indicated even in the presence of normal blood pressure
- Renal replacement therapy (e.g., hemodialysis, peritoneal dialysis, and renal transplantation) should be used in people with SCD if needed

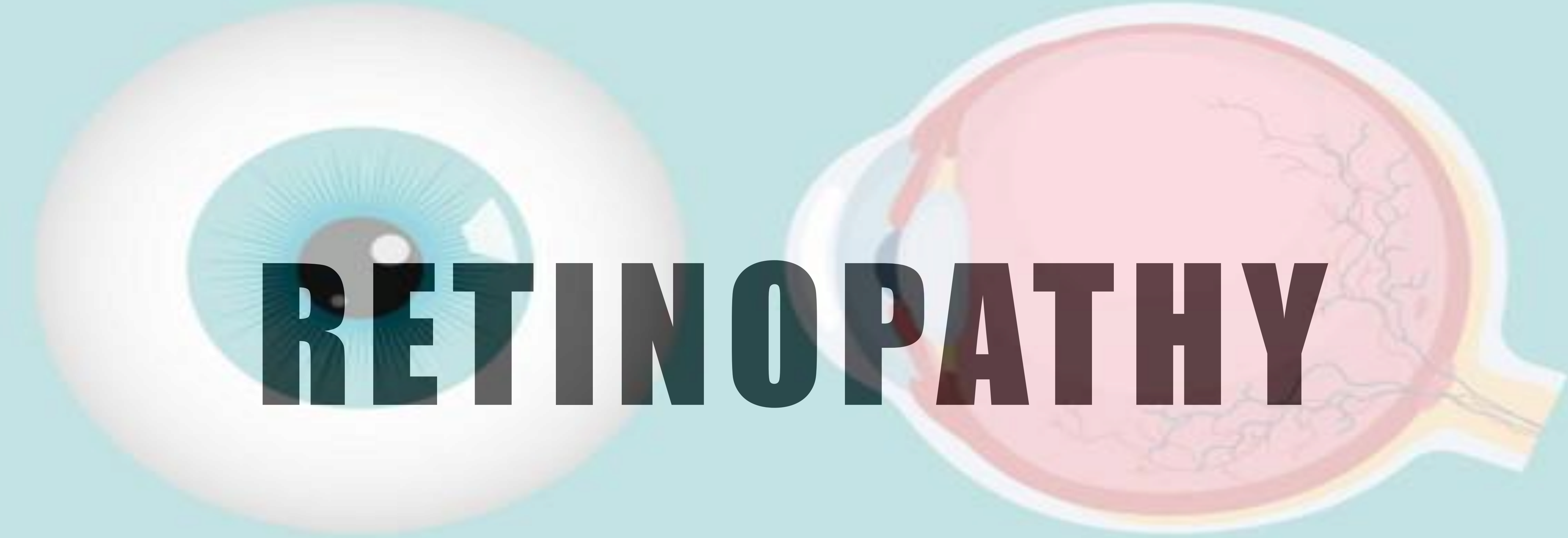

RETINOPATHY

RETINOPATHY

- Occurs from retinal artery occlusion and ischemia, with associated proliferative retinopathy, vitreal haemorrhage, severe vision impairment, and retinal detachment.
- Observed in older children and adolescents and tend to progress throughout adulthood.
- Unlike other complications, which tend to occur with greater frequency in Hb SS, proliferative retinopathy is more common in Hb SC disease than in other SCD genotypes

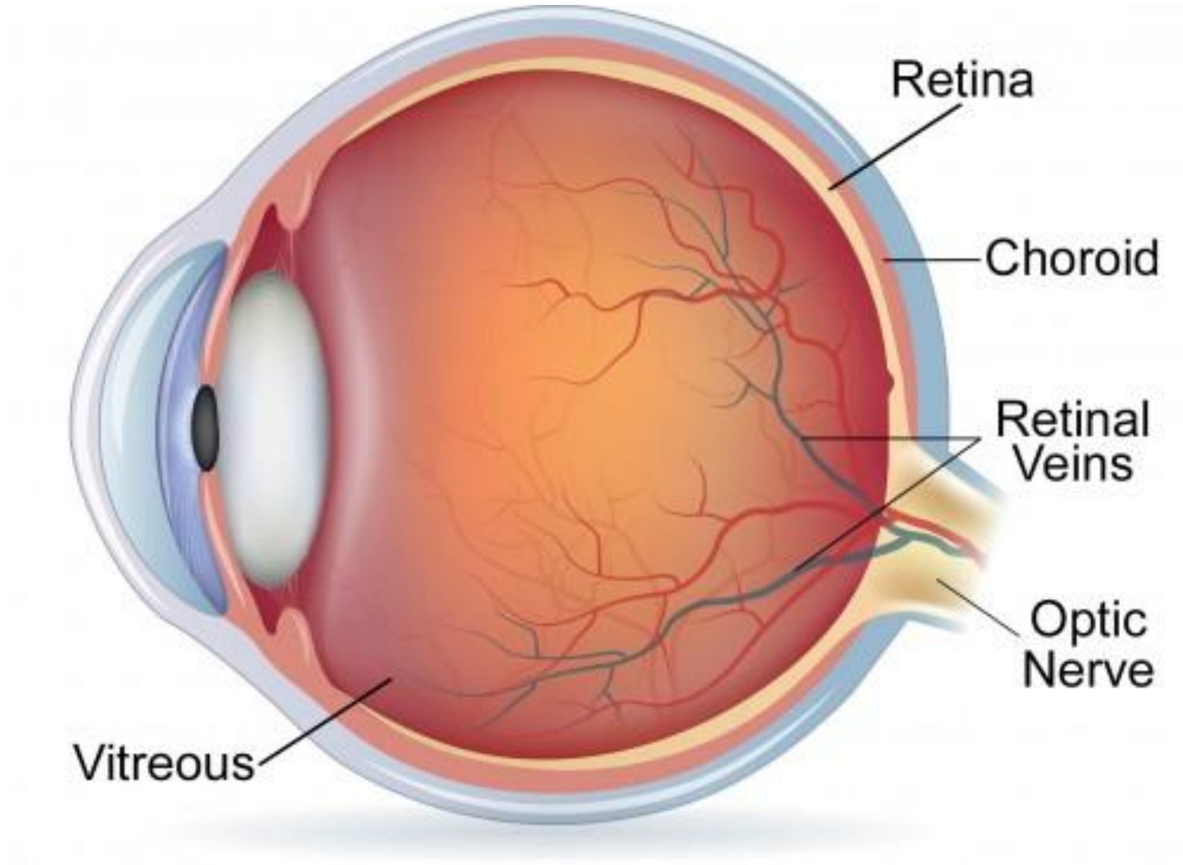

RETINOPATHY

- Infarction and ischemia typically begin in the peripheral retina, followed by neovascularization, which may be facilitated by the production of angiogenic factors such FGF and VEGF.
- Nonproliferative or background sickle retinopathy includes the following manifestations:
  - Venous tortuosity
  - Salmon-patch haemorrhage
  - Schisis cavity
  - The black sunburst

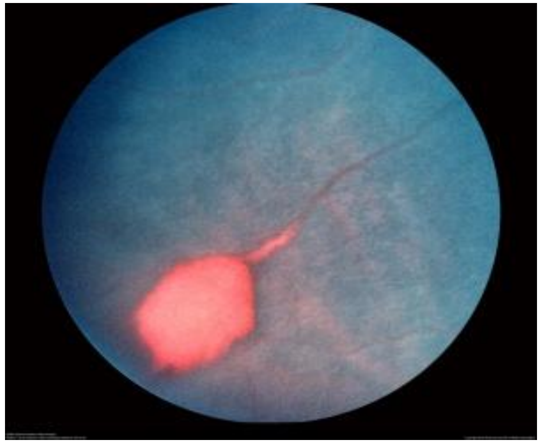

RETINOPATHY

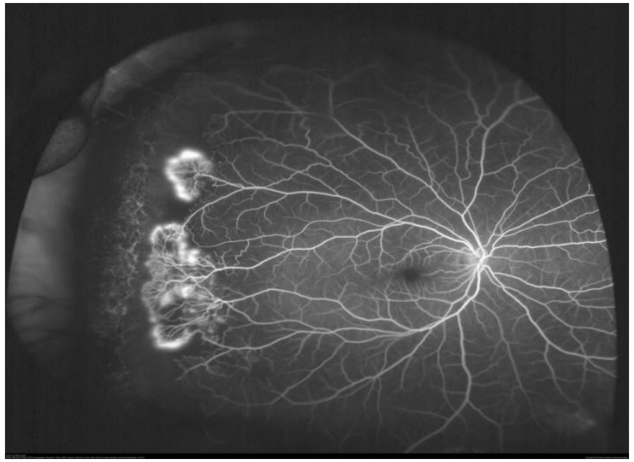

- Proliferative sickle retinopathy (PSR) is the most severe ocular change in SCD
- PSR is progressive. A desirable objective is to treat the neovascular tissue before a vitreous haemorrhage occurs.
- Goldberg classified PSR into the following 5 stages:
  1. Peripheral arteriolar occlusions
  2. Arteriolar-venular anastomosis
  3. Neovascular proliferation
  4. Vitreous haemorrhage
  5. Retinal detachment

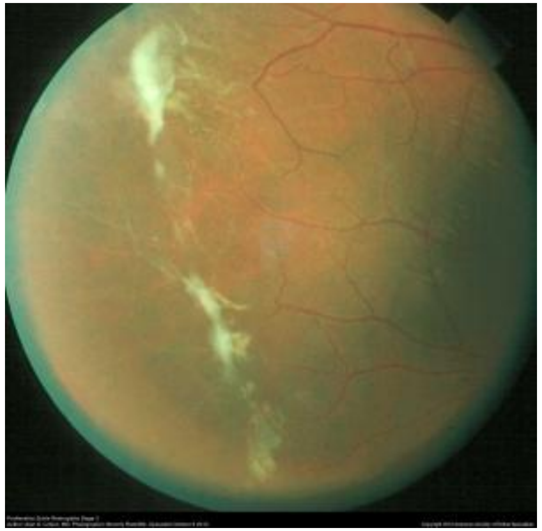

APPROACH TO MANAGEMENT

- Refer a person with SCD to an ophthalmologist for a dilated eye examination to evaluate for retinopathy beginning at age 10 years.
- Re-screen for retinopathy at 1–2year intervals, if the results remain normal.
- Refer people with suspected retinopathy to a retinal specialist ophthalmologist for management.
- Refer persons of all ages with proliferative sickle retinopathy (PSR) to an ophthalmologist (retina specialist) for evaluation and possible laser photocoagulation therapy.
- Refer children and adults with vitreoretinal complications of PSR refractory to medical treatment for evaluation and possible vitrectomy.

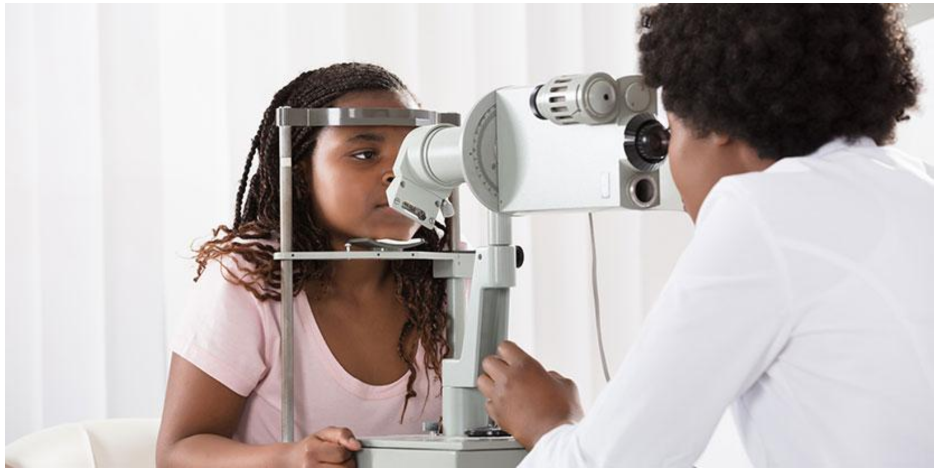

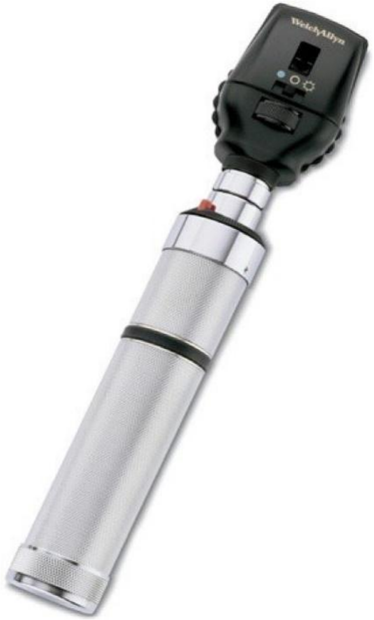

CHRONIC LEG ULCER

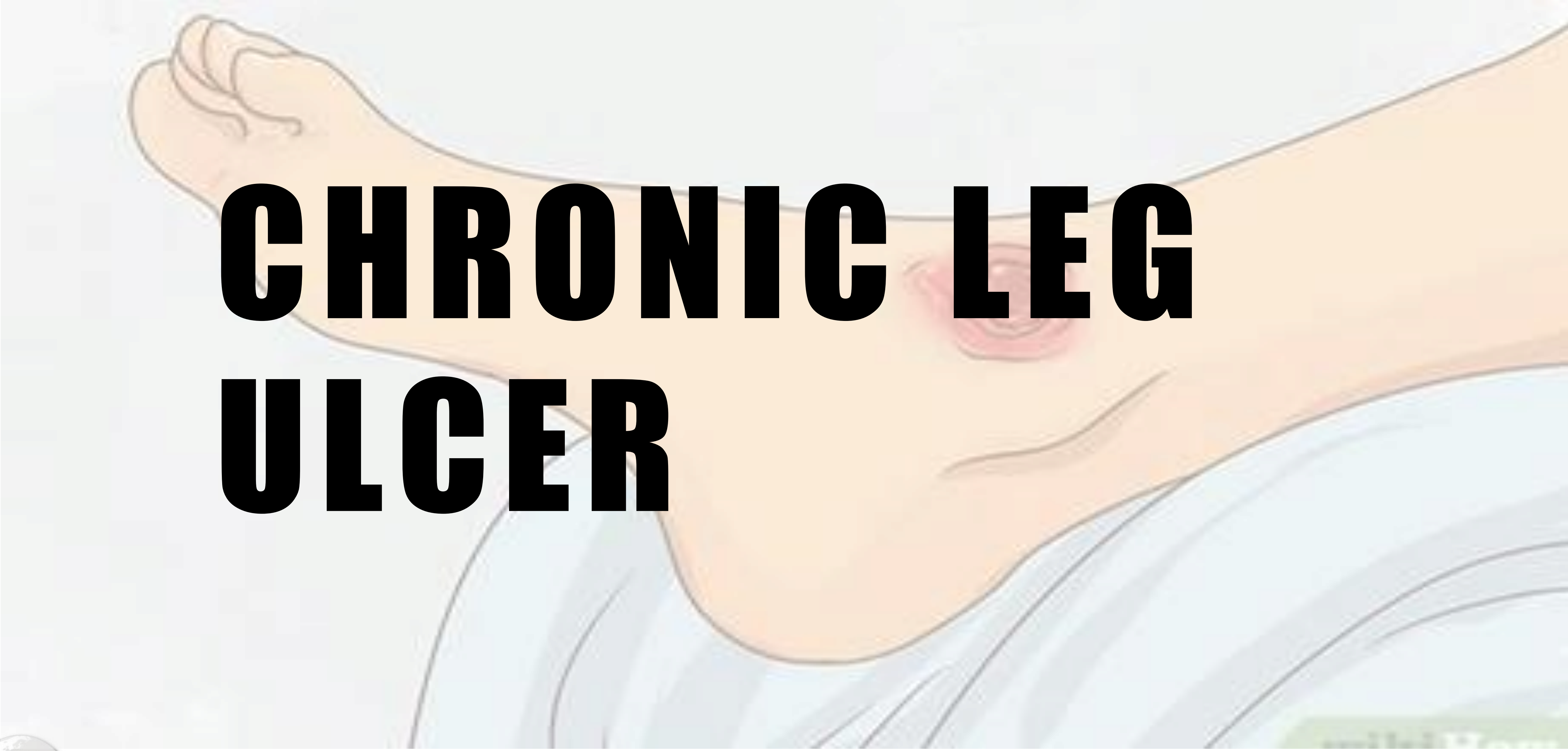

LEG ULCER

- Vaso-occlusion in the skin can produce leg ulcers in patients with SCD.
- Can cause significant pain, physical disability, and negative psychologic and social impacts.
- Usually present after the age of 10 years and are more common in males than females.
- Ulcers may develop spontaneously or after trauma.
- Typical sites include the medial and lateral malleolus. Bilateral involvement is common.
- Ulcers may become superinfected.

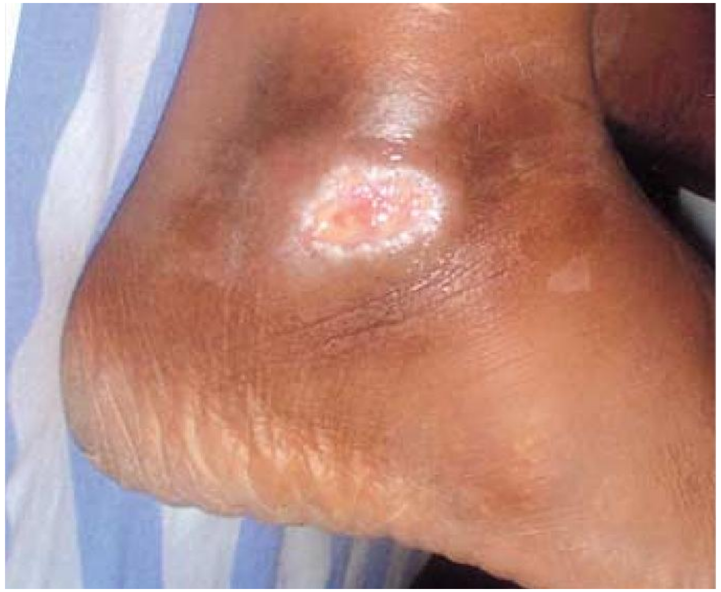

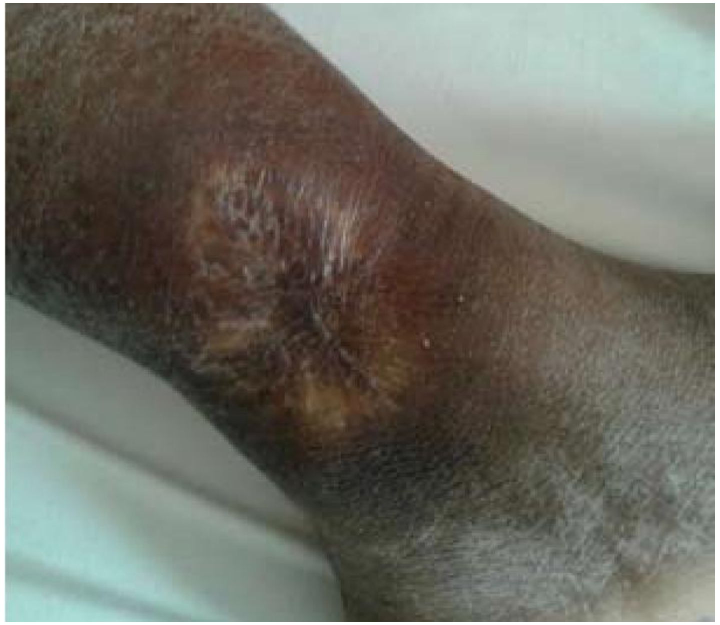

APPROACH TO MANAGEMENT OF LEG ULCERS

- Assess the wound thoroughly. Document the size, appearance,
- status of the surrounding skin, presence of tenderness, edema and femoral lymph nodes enlargement.
- Do CBC and blood culture and sensitivity if associated with cellulitis or abscess formation.
- Do imaging studies e.g., X-rays, MRI to exclude osteomyelitis if there is fever and bone tenderness
- Apply wet-to-dry dressings twice a day
- Consult the surgical team for surgical debridement and skin grafting when indicated
- Give zinc supplementation
  Apply graduated compression bandages to reduce lymphoedema
  Elevate feet when sitting to improve blood circulation to the ulcer

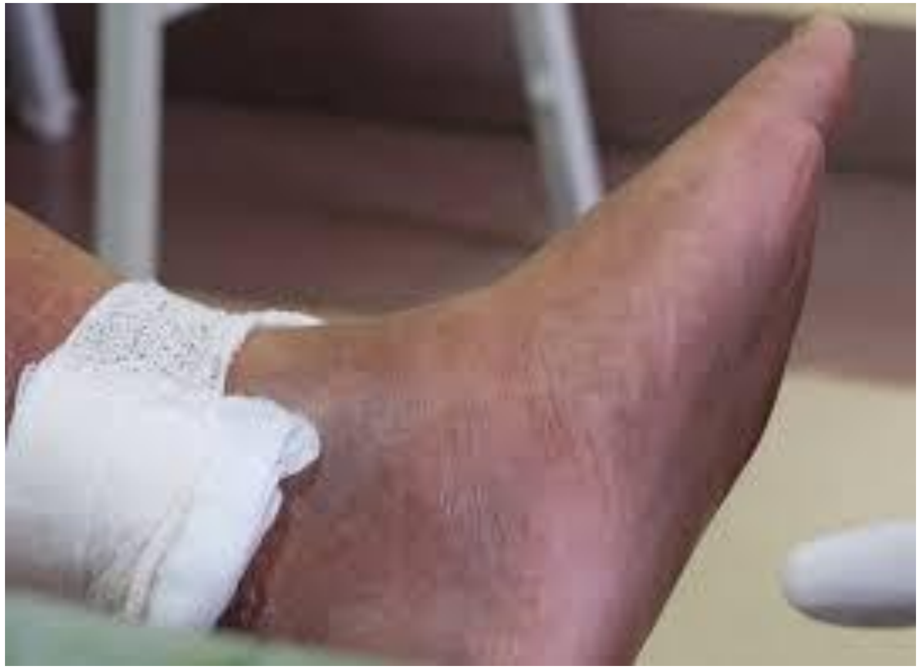

APPROACH TO MANAGEMENT OF LEG ULCERS

- Treat the pain with adequate and appropriate analgesia. Usually, the pain is neuropathic in nature and it is treated with amitriptyline or newer antiepileptic drugs. (See pain management in SCD guidelines)
- Take biopsy for histopathology when malignancy is suspected. Consult vascular surgeons if features of arterial insufficiency present.
- Consider regular blood transfusion until the wound heals if the above measures fail.
- Consider discontinuation of Hydroxyurea in patients with non-healing or slowly healing ulcer.
  Explain to the patient that ulcer may take long time to heal. Also, enforce the prevention measures to reduce the risk of recurrence.

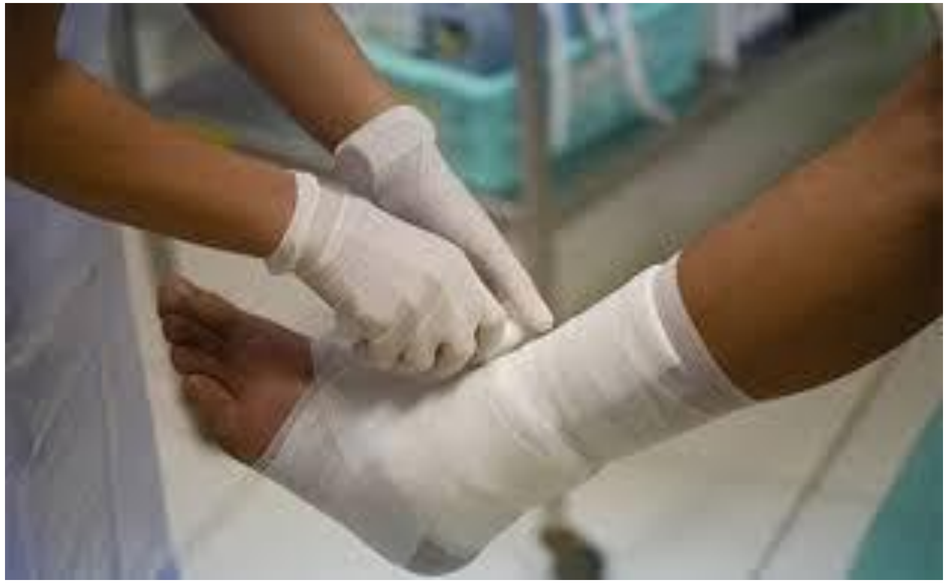

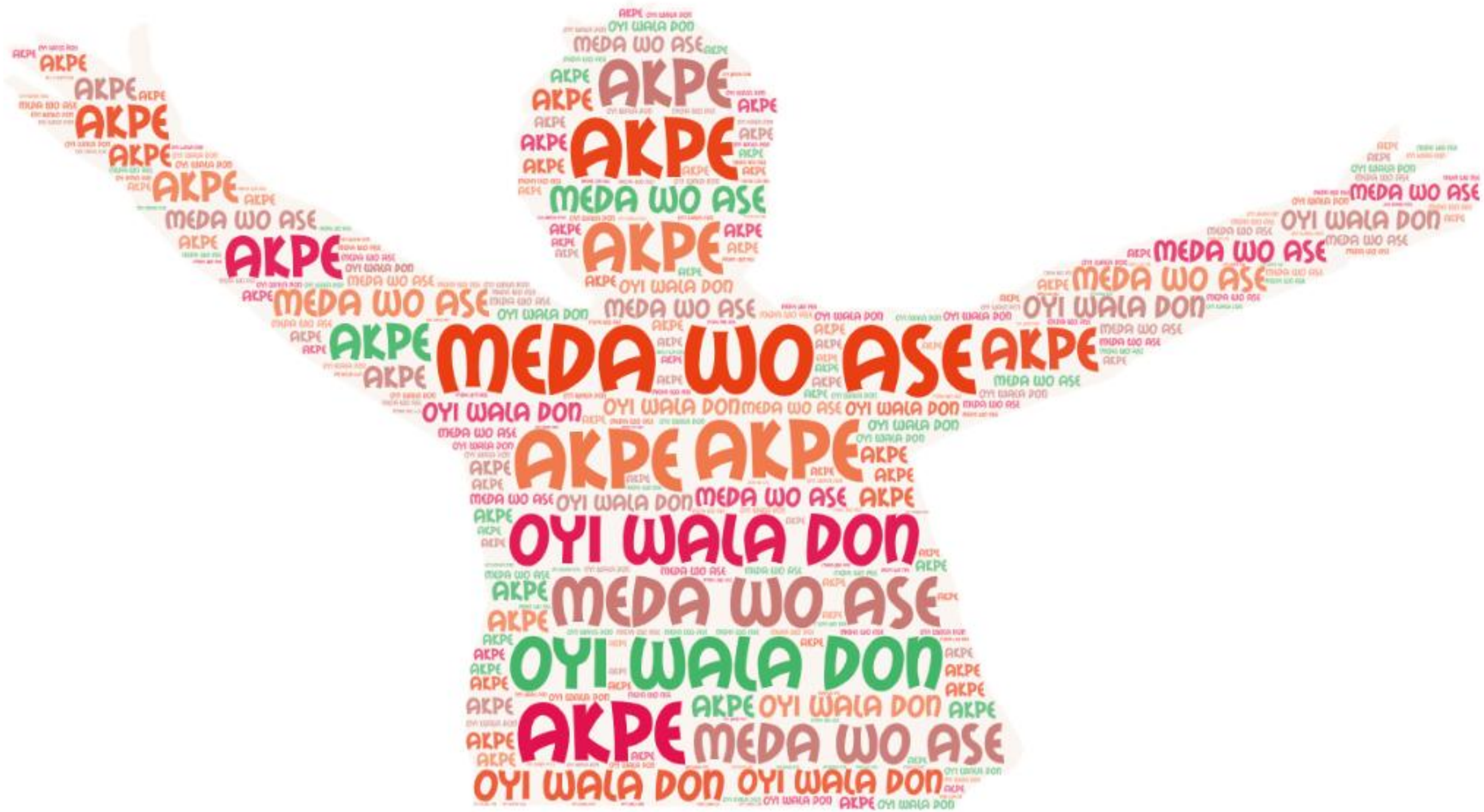
MEDA WO ASE
AKPE
OYI WALA DON
