# Supplementary material for: Healthcare providers’ knowledge on sickle cell disease and its management: A pre- and post-training test evaluation outcome
Source: PLoS One. 2025 Sep 8;20(9):e0332069. doi: 10.1371/journal.pone.0332069 (PMC12416636; doi:10.1371/journal.pone.0332069)
Supplement: Attachment — Submitted filename: Response to reviewers letter.docx [file pone.0332069.s008.docx]

21^st^ August, 2025

**The Editor**

**PLOS ONE**

Dear Editor,

Below is the point to point response to reviewers’ comments

**Response to Reviewer comments on Manuscript PONE-D-25-17578R2**

| **Comments** | **Response** |
| --- | --- |
| Adding the slides as a supplemental file would be helpful. | The slides have been added on as supplementary file |
| There was no description of the validation of the knowledge test in the submission. For example, how was content validity determined? The lack of a validated knowledge test should be included in the limitations. | This has been added to the limitations and inserted in lines 289-290.  Also, a recommendation has also been inserted in lines 293-295 for validating the knowledge test |
| Adding that the following would be done in future studies in the manuscript as part of this reviewer’s original comments would be helpful: “. The manuscript could also mention the importance of measuring patient-care outcomes after this intervention” | A discussion on this has been inserted in lines 272-275. And it has also been added on in lines 291-293 as a recommendation for future studies. |

Thank you

Dr Vivian Paintsil
