# Supplementary material for: Healthcare providers’ knowledge on sickle cell disease and its management: A pre- and post-training test evaluation outcome
Source: PLoS One. 2025 Sep 8;20(9):e0332069. doi: 10.1371/journal.pone.0332069 (PMC12416636; doi:10.1371/journal.pone.0332069)
Supplement: Attachment — Submitted filename: PLOS reviewer comments.docx [file pone.0332069.s005.docx]

**Reviewer Comments on Manuscript PONE-D-25-17578**

This manuscript addresses an important gap in the care of individuals with sickle cell disease (SCD) by evaluating the impact of a standardized training program on healthcare providers' knowledge in Ghana. This is a well-executed and relevant study. With a few clarifications and refinements, it will make a valuable contribution to literature on capacity building for SCD care in low-resource settings.

**Strengths:**

- Comprehensive training content based on the SPARCO guidelines.
- Large sample size (n=543) with representation from multiple facility levels.
- Clear demonstration of knowledge improvement post-training, with useful subgroup analysis by cadre and facility type.

**Additional considerations:**

1. **Lack of Long-Term Follow-Up**: The post-training assessment was immediate. A follow-up at 3–6 months would help assess retention and sustained impact.
2. **Validation of Assessment Tool**: The manuscript does not clarify whether the pre/post-test questions were piloted or validated. Including psychometric properties (e.g., internal consistency) would strengthen the evaluation.
3. **Cutoff for Knowledge Scores**: Comprehensive work. That said, I wonder why the cutoffs were 0–9 (poor) and 10–20 (good). A three-tier system (e.g., poor, fair, good) may give a more nuanced view of the distribution and changes across knowledge levels.
4. **Selection Bias**: Since participation was based on invitations, there may be a bias toward more engaged or motivated providers. This should be briefly acknowledged in the limitations.
5. **Participant feedback:** Consider including a brief participant feedback component in future work to guide content refinement.
6. **Barriers to implementation:** Expand on the discussion of barriers to guideline uptake, especially since only 43% reported using one.
